# A systematic survey shows that reporting and handling of missing outcome data in networks of interventions is poor

**DOI:** 10.1186/s12874-018-0576-9

**Published:** 2018-10-24

**Authors:** Loukia M. Spineli, Juan J. Yepes-Nuñez, Holger J. Schünemann

**Affiliations:** 1 Institut für Biometrie (OE 8410), Medizinische Hochschule Hannover, Carl-Neuberg-Straße 1, 30625 Hannover, Germany; 2 Department of Health Research Methods, Evidence and Impact, McMaster University, 1280 Main St. West, Hamilton, ON L8S 4K1 Canada; 3 School of Medicine, University of Antioquia, Calle 70 No. 52 – 21, Medellín, Colombia; 4 Department of Medicine, McMaster University, 1280 Main St. West, Hamilton, ON L8S 4K1 Canada

**Keywords:** Missing outcome data, Systematic review, Network meta-analysis, Intention-to-treat analysis, Empirical research

## Abstract

**Background:**

To provide empirical evidence about prevalence, reporting and handling of missing outcome data in systematic reviews with network meta-analysis and acknowledgement of their impact on the conclusions.

**Methods:**

We conducted a systematic survey including all published systematic reviews of randomized controlled trials comparing at least three interventions from January 1, 2009 until March 31, 2017.

**Results:**

We retrieved 387 systematic reviews with network meta-analysis. Description of missing outcome data was available in 63 reviews. Intention-to-treat analysis was the most prevalent method (71%), followed by missing outcome data investigated as secondary outcome (e.g., acceptability) (40%). Bias due to missing outcome data was evaluated in half the reviews with explicit judgments in 18 (10%) reviews. Only 88 reviews interpreted their results acknowledging the implications of missing outcome data and mostly using the network meta-analysis results on missing outcome data as secondary outcome. We were unable to judge the actual strategy applied to deal with missing outcome data in 65% of the reviews due to insufficient information. Six percent of network meta-analyses were re-analyzed in sensitivity analysis considering missing outcome data, while 4% explicitly justified the strategy for dealing with missing outcome data.

**Conclusions:**

The description and handling of missing outcome data as well as the acknowledgment of their implications for the conclusions from network meta-analysis are deemed underreported.

**Electronic supplementary material:**

The online version of this article (10.1186/s12874-018-0576-9) contains supplementary material, which is available to authorized users.

## Background

Missing outcome data (MOD) are a distinct type of missing information attributed to multifaceted causes that prematurely terminate the participation in a research study, including a clinical trial. These causes may relate to the design and conduct of the clinical trial or be completely irrelevant to the clinical trial structure. Besides sample size losses, discontinuation may distort the balance of the baseline characteristics between the compared groups in a trial leading to confounding and selection bias [1–6]. Furthermore, participants remaining in the trial might not be representative of the population originally recruited and reduce directness of the findings [1–3, 7].

Missing outcome data are an integral part of a systematic review as they emerge inevitably through the inclusion of clinical trials with reported participant losses. Since the risk of bias due to MOD at the trial level is likely to translate into a similar risk at the meta-analysis level, integration of trials with MOD may lead to meta-analysis results that do not reflect the impact of the interventions in practice [8–11]. Consequently, strategic management of MOD in a systematic review is particularly necessary [4, 5].

There is guidance for systematic reviewers, meta-analysts, journal editors and reviewers in order to improve reporting and handling of MOD at meta-analysis level [9, 12–14]. In addition, statistical methods, software and tutorials for dealing with MOD of varying complexity have been developed over time [8, 11, 14–23]. Nevertheless, MOD in systematic reviews with network meta-analysis have received very little attention. Network meta-analysis (NMA) is an extension of pairwise meta-analysis that allows direct (i.e., trials investigating the same comparison) and indirect (i.e., different sets of trials that compare each intervention with a common comparator) evidence to be synthesized simultaneously in a single analysis in order to yield relative intervention effects for all possible comparisons and by extent, to rank the multiple competing interventions [24]. While methods for handling MOD in pairwise meta-analysis may be applicable to NMA, addressing MOD in the context of NMA holds an additional degree of complexity. This complexity inherently stems from the additional assumptions required to integrate different pieces of evidence from multiple trials in a single analysis and obtain internally coherent relative treatment effects for all pairwise comparisons [25]. Therefore, the presence of MOD in a network of interventions might materially impact on additional parameters beyond the standard meta-analytic ones (i.e., mean effect and between-study variance) including incoherence of the estimates between direct and indirect evidence for a particular comparison, probabilistic statements on the comparability of all competing interventions and intervention ranking.

Currently, publications on MOD in the NMA context are restricted to a post hoc evaluation that uses the informative missingness odds ratio (IMOR) parameter [26] to assess the impact of missing binary outcome data on NMA results; an extension of the IMOR parameter for missing *continuou*s outcome data [18] and a meta-epidemiological study that investigates the association between specific trial characteristics and the likelihood of premature discontinuation in antipsychotic trials for schizophrenia [27].

While the recommendations on the reporting and handling of MOD in conventional systematic reviews are of great relevance and importance also for systematic reviews with NMA, they can only partially assist the interested audience since they do not reflect upon the additional NMA components, namely, consistency assumption and intervention ranking. Properly established guidelines cannot exist without empirical evidence and to our knowledge there is no empirical evidence on the reporting and handling of MOD in networks with multiple interventions (e.g., similar to [12]). The aim of this study was to bridge this particular knowledge gap by providing evidence about the impact of MOD on the credibility of a systematic review with NMA, including the reporting and handling of MOD as well as the acknowledgment of their implications.

## Methods

### Eligible systematic reviews

We used several databases to identify NMAs of at least three interventions. First, we based our sample on the published database of Zarin et al. [28]. This database includes 456 systematic reviews with NMA of randomized controlled trials (RCTs) in all languages with *at least four* different interventions from inception until April 14, 2015. The authors excluded systematic reviews (i) of diagnostic test accuracy studies or genetic studies or observational studies or mixture of RCTs and observational studies; (ii) those that included a smaller number of trials than interventions; and (iii) had implemented naïve indirect comparison methods.

Second, we considered also the databases by Tan et al. and Bafeta et al. as they included *at least three* interventions and we added these additional potential NMAs in our database [29, 30]. These databases covered a period from 1997 [29] to July 2012 [30]. Third, we searched in addition the database of Nikolakopoulou et al. [31] (they searched from inception to December 2012) to locate possible systematic reviews that might have been missed by Zarin et al. [28].

Finally, to make the sample of systematic reviews current, we conducted our own search using the eligibility criteria (i) – (iii) considered in Zarin et al. [28] for eligible systematic reviews published from August 2012 to March 2017 that assessed at least 3 interventions. For that search, we applied the strategies developed by Petropoulou et al. [32] (and used in Zarin et al. [28] as well) for MEDLINE, EMBASE and the Cochrane Database of Systematic Reviews.

We included reviews published from 2009 and onwards, because the new Cochrane risk of bias tool was published during 2009 and hence, we expected the reviewers to have routinely assessed the included studies also in terms of bias due to MOD [12]. We considered whether RCTs with ‘non-standard’ design, such as quasi, crossover, factorial, cluster, split-mouth, contralateral and split-face/body RCTs, were among the eligible trials. Since the methodology to handle MOD has been developed primarily in the context of standard RCTs, we decided to exclude systematic reviews that included RCTs with ‘non-standard’ design. Furthermore, we excluded commentaries, letters, editorials, case-series, protocols, methodological articles, overviews of systematic reviews (MOD have been addressed already within the systematic review), cost-effectiveness reviews of multiple interventions that did not perform de novo systematic review with NMA (but used results from published NMAs, published meta-analyses and selected trials) and systematic reviews that investigated MOD as a single primary outcome usually termed as ‘acceptability’ or ‘withdrawal due to adverse events or any reason’.

### Eligible network meta-analyses

From each eligible systematic review, we selected only one primary outcome. When the authors described more than one primary outcomes, we gave priority to binary outcomes, since established methods to handle MOD in systematic reviews address mainly binary outcomes. In the presence of many *binary* primary outcomes, we selected a patient-important outcome following the hierarchy of outcomes as defined by Akl et al. [33].

### Screening

Two reviewers (LMS, JJYN) double-screened in a standardized approach the titles and abstracts of the systematic reviews already included in Bafeta et al. [30], Tan et al. [29] and Nikolakopoulou et al. [31] as well as those retrieved from the updated literature search. Reasons for exclusion were documented. Then, potentially relevant systematic reviews were screened in full-text. In case of conflicts, a third reviewer (HJS) was consulted.

### Data collection process and data items

We developed a data extraction form and piloted on randomly-selected eligible systematic reviews in order to determine the finalized content of the extraction form. One reviewer (LMS) extracted all necessary information from the eligible systematic reviews. A second reviewer (JJYN) randomly selected and checked 10% of the extracted systematic reviews for potential errors. Disagreements were resolved by discussion between the reviewers; in case of no concordance, the authors consulted a third reviewer (HJS).

### Systematic review level

We extracted items that referred to specific domains defined separately for the systematic review as a whole and the selected NMA. Specifically, at the systematic review level, we extracted information on general characteristics of the review, such as year of publication, disease condition, protocol availability and incorporation of the GRADE (Grading of Recommendations, Assessment, Development and Evaluation) approach. Items referring to reporting and handling of MOD in the protocol and published report of the systematic review included the definition of MOD, MOD strategy and adherence to the pre-specified strategy (if a protocol exists), possible explanation provided by the authors to support their strategy, use of the last-observation-carried-forward (LOCF) approach (i.e., for each participant who withdrew during the course of a trial with longitudinal follow-up, the last observed continuous outcome is forwarded to the remaining pre-specified outcome assessments in order to fill-in the subsequent missing responses [1, 2]) and assessment of bias due to MOD. To locate these items, we also searched the PROSPERO registry (if a registration number was provided) or available Web addresses and published additional file.

Lastly, we extracted information on whether and how authors dealt with the implications of MOD on NMA components (i.e. treatment effects, consistency parameter, intervention ranking, heterogeneity and possible model fit parameters); for instance, we noted authors’ judgments when they used the risk of bias assessment for single studies or the GRADE approach for a body of evidence, remarks on the impact of dropout prevalence and reasons, and discussion of the results of any moderator analyses in the context of MOD, such as subgroup analysis where studies were split into subgroups of low and high risk of bias due to MOD, meta-regression analysis using dropout prevalence as covariate, and sensitivity analysis by excluding trials without intention-to-treat analysis (namely, analysis of all participants in the group they were originally randomized irrespectively protocol compliance or withdrawal [1–3]). This information was sought in the abstract, results, discussion and conclusion sections of the publication.

### Selected network meta-analysis level

For the selected NMAs, we extracted information on network topology (number of interventions and included studies and network shape), type of intervention-comparison and outcome definition (as defined by Turner et al. [34]) and effect measure. For the selected primary outcome, we recorded the reported choice of analysis set (i.e., (modified) intention-to-treat and per-protocol) and then, we judged the actual method of analysis rather than the reported (e.g., available case analysis or imputation with or without LOCF). Furthermore, information was extracted on the accountability of the uncertainty induced by missingness and specific scenarios considered when imputing MOD.

We evaluated whether the authors applied a sensitivity analysis and what strategies they considered to inspect further the impact of MOD for the selected primary outcome. We documented whether the authors reported any changes in the inferences after sensitivity analysis. Lastly, we determined which systematic reviews provided extractable data information for the selected primary outcome and MOD.

## Results

### Systematic reviews and network meta-analysis selection

**Fig. 1:**
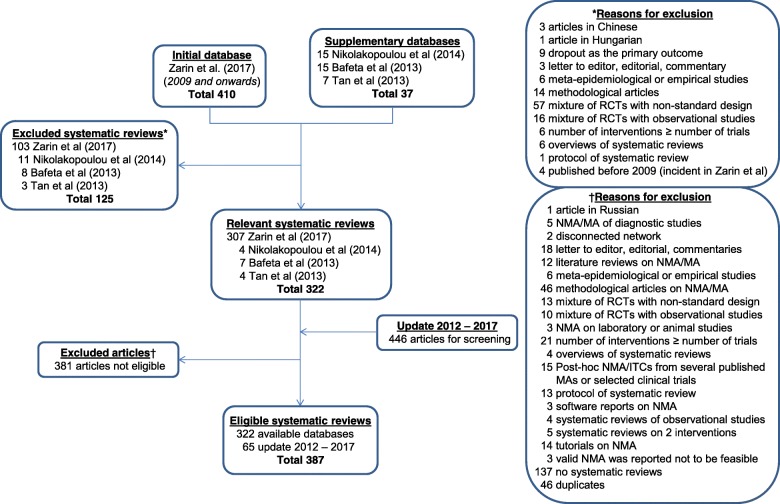
Flow diagram of systematic reviews with network meta-analysis. ITC, indirect treatment comparison; MA, meta-analysis; NMA, network meta-analysis; RCT, randomized controlled trial

A total of 410 studies located in Zarin et al. [28] were relevant for screening. After supplementing with studies identified in Nikolakopoulou et al. [31], Bafeta et al. [30] and Tan et al. [29], a total of 447 reviews were considered for full-text screening. Of those, 125 were excluded for several reasons (Fig. 1).

The bibliographic search from August 2012 to March 2017 yielded a total of 446 possibly relevant articles for title and abstract screening. Of those, 46 were duplicate records and 137 had no relevance to systematic review and NMA (e.g., studies and methodology of genetic, laboratory and animal content, cohort studies, cross-sectional studies, case-control studies and case reports). Then, a total of 263 articles were considered for full-text screening. Of those, 198 were excluded for various reasons (Fig. 1). A list with the excluded articles and their reasons can be found in Additional file 1. In total, 387 systematic reviews were considered eligible for data extraction (see, Additional file 2).

### Characteristics of selected systematic reviews

**Fig. 2:**
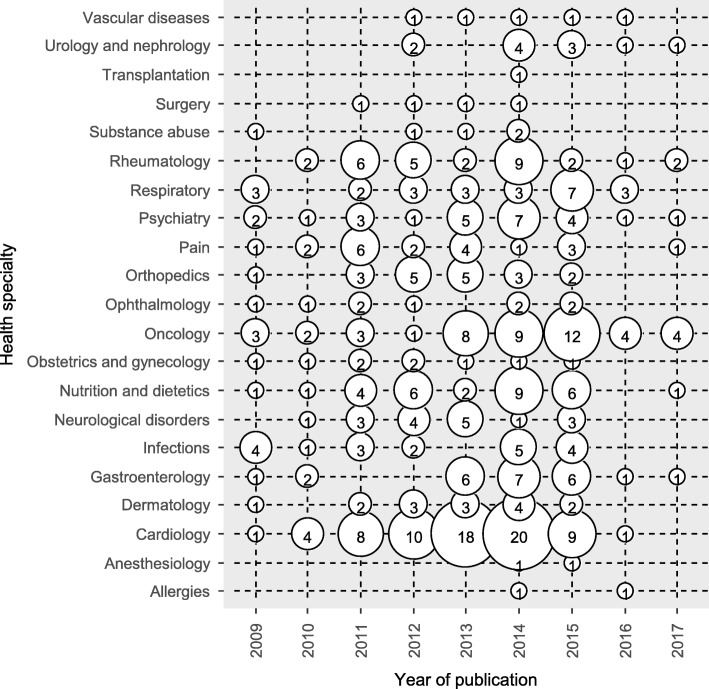
Bubble chart of 387 systematic reviews with network meta-analysis by year of publication and health specialty

**Fig. 3:**
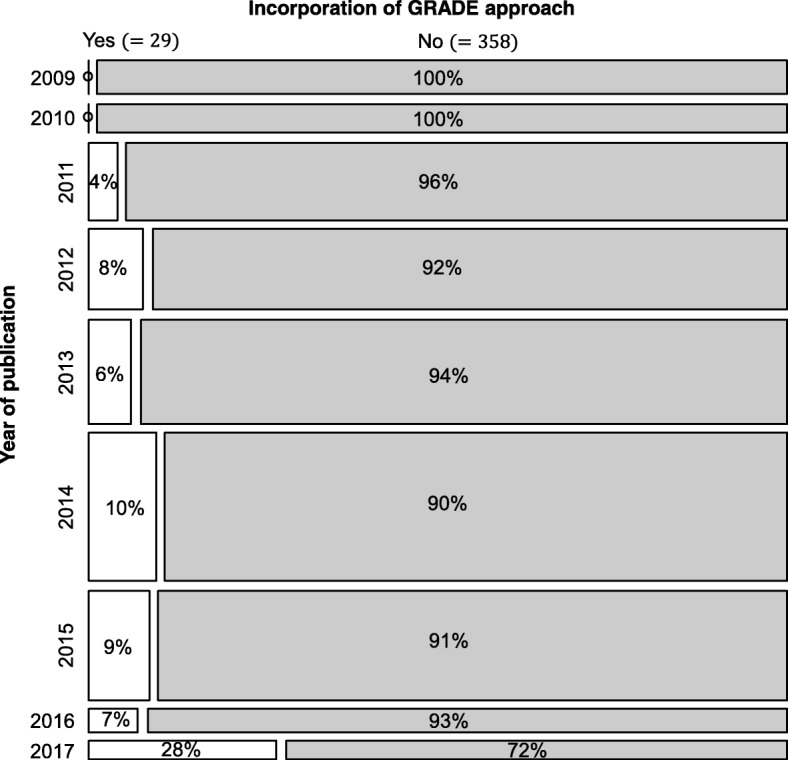
Mosaic plot of 387 systematic reviews with network meta-analysis to visualize the frequency of incorporation of GRADE approach by year of publication. GRADE, Grading of Recommendations, Assessment, Development and Evaluation

The majority were published during 2014 (*n* = 92, 24%), specialized on cardiology (*n* = 71, 18%) and oncology (*n* = 46, 12%) (see Fig. 2 for further details), were non-Cochrane reviews (*n* = 380, 98%), did not mention in their publication whether a protocol was available (*n* = 279, 72%) and did not incorporate the GRADE approach in their results (*n* = 358, 92%, Fig. 3). Sixty-six reviews (17%) mentioned that a protocol was developed but they did not make the protocol available (Table 1). Only 38 (10%) reviews registered or published a protocol with the majority being found between 2013 and 2015.

**Table 1:** Characteristics of 387 systematics reviews in the context of missing outcome data

Characteristic	Levels		Total		2009 (*n* = 21)		2010 (*n* = 18)		2011 (*n* = 48)		2012 (*n* = 50)		2013 (*n* = 65)		2014 (*n* = 92)		2015 (*n* = 68)		2016 (*n* = 14)		2017 (*n* = 11)	
			n	%																		
*Addressing and handling missing outcome data*																						
Study protocol	Registered		24	6.2	0	0.0	0	0.0	1	4.2	1	4.2	6	25.0	8	33.3	8	33.3	0	0.0	0	0.0
	Not registered but published		14^a^	3.6	1	7.1	0	0.0	2	14.3	2	14.3	6	42.9	1	7.1	2	14.3	0	0.0	0	0.0
	Mentioned but not available		66	17.1	4	6.1	3	4.5	12	18.2	6	9.1	10	15.2	17	25.8	9	13.6	2	3.0	3	4.5
	Explicitly mentioned that there is no protocol		4	1.0	0	0.0	0	0.0	0	0.0	1	25.0	0	0.0	3	75.0	0	0.0	0	0.0	0	0.0
	Protocol not mentioned		279	72.1	16	5.7	15	5.4	33	11.8	40	14.3	43	15.4	63	22.6	49	17.6	12	4.3	8	2.9
If protocol is available (*37 SRs*), the MOD strategy was determined already in the protocol^b^	Yes, using MOD as secondary outcome		5	13.5	1	0.2	0	0.0	0	0.0	0	0.0	3	0.6	1	0.2	0	0.0	0	0.0	0	0.0
	Yes, in primary analysis using ITT with clarifications		7	18.9	1	14.3	0	0.0	1	14.3	1	14.3	2	28.5	1	14.3	1	14.3	0	0.0	0	0.0
	Yes, in primary analysis using ITT without clarifications		2	5.4	0	0.0	0	0.0	1	50.0	0	0.0	1	50.0	0	0.0	0	0.0	0	0.0	0	0.0
	Yes, in primary analysis by excluding trials with MOD		1	2.7	0	0.0	0	0.0	0	0.0	0	0.0	0	0.0	0	0.0	1	100	0	0.0	0	0.0
	Yes, in primary analysis by excluding participants with MOD		1	2.7	0	0.0	0	0.0	0	0.0	0	0.0	1	100	0	0.0	0	0.0	0	0.0	0	0.0
	No MOD strategy was determined		23	62.2	0	0.0	0	0.0	1	4.3	1	4.3	7	30.4	7	30.4	7	30.4	0	0.0	0	0.0
If protocol is available with a MOD strategy (*14 SRs*), the strategy defined in the protocol differed from that applied in the review	No, the authors adhered to the protocol		12	85.7	0	0.0	0	0.0	2	16.7	1	8.3	1	8.3	4	33.3	4	33.3	0	0.0	0	0.0
	MOD were not addressed eventually		2	14.3	0	0.0	0	0.0	0	0.0	0	0.0	0	0.0	1	50.0	1	50.0	0	0.0	0	0.0
The authors explained (in the protocol or review) what they considered as MOD	Yes, an explicit definition is provided		63	16.3	4	6.3	7	11.1	13	20.6	4	6.3	12	19.0	11	17.5	8	12.7	3	4.8	1	1.6
	No explanation is provided		324	83.7	17	5.2	11	3.4	35	10.8	46	14.2	53	16.4	81	25.0	60	18.5	11	3.4	10	3.1
The review explicitly reported whether LOCF was employed or not in the included trials	Yes, and they distinguished between LOCF and completely MOD		5	1.3	0	0.0	1	20.0	1	20.0	0	0.0	2	40.0	0	0.0	0	0.0	0	0.0	1	20.0
	Yes, but they didn’t distinguish between LOCF and completely MOD		18	4.6	0	0.0	1	5.6	4	22.2	2	11.1	3	16.7	6	33.3	1	5.6	0	0.0	1	5.6
	No		364	94.1	21	5.8	16	4.4	43	11.8	48	13.2	60	16.5	86	23.6	67	18.4	14	3.8	9	2.5
There is no evidence that MOD exist in the included trials for the primary outcomes	MOD are present in the network		273	70.5	10	3.7	14	5.1	35	12.8	29	10.6	44	16.1	76	27.8	44	16.1	13	4.8	8	2.9
	Yes		1	0.3	0	0.0	0	0.0	0	0.0	0	0.0	0	0.0	0	0.0	1	100	0	0.0	0	0.0
	Nothing mentioned		113	29.2	11	9.7	4	3.5	13	11.5	21	18.6	21	18.6	16	14.2	23	20.4	1	0.9	3	2.7
If the review explicitly reported the presence of MOD (*273 SRs*), the strategy described in the Methods section to address MOD is^b^	by excluding participants with MOD from the analyses		4	1.5	0	0.0	0	0.0	1	25.0	1	25.0	0	0.0	1	25.0	0	0.0	0	0.0	1	25.0
	using ITT in the primary analysis with clarifications		25	9.2	2	8.0	1	4.0	3	12.0	3	12.0	4	16.0	8	32.0	3	12.0	0	0.0	1	4.0
	using ITT in the primary analysis without further clarifications		84	30.8	1	1.2	1	1.2	10	11.9	10	11.9	20	23.8	23	27.4	14	16.7	3	3.6	2	2.3
	using dropout as a secondary outcome		61	22.3	4	6.6	4	6.6	12	19.7	4	6.6	14	22.9	11	18.0	7	11.5	3	4.9	2	3.2
	using sensitivity analysis	by excluding studies based on a MOD rate threshold	6	2.2	2	33.3	0	0.0	1	16.7	0	0.0	1	16.7	1	16.7	1	16.7	0	0.0	0	0.0
		by excluding participants with MOD	6	2.2	0	0.0	1	16.7	1	16.7	1	16.7	0	0.0	2	33.3	1	16.7	0	0.0	0	0.0
		other	4	1.5	0	0.0	0	0.0	0	0.0	1	25.0	0	0.0	0	0.0	2	50.0	1	25.0	0	0.0
	using subgroup analysis		6	2.2	0	0.0	1	16.7	0	0.0	0	0.0	3	50.0	2	33.3	0	0.0	0	0.0	0	0.0
	using meta-regression analysis		3	1.1	0	0.0	0	0.0	0	0.0	0	0.0	1	33.3	2	66.7	0	0.0	0	0.0	0	0.0
	Not mentioned		120	44.0	4	3.3	9	7.5	13	10.8	11	9.2	14	11.7	34	28.3	24	20.0	7	5.9	4	3.3
In case of ‘intention-to-treat analysis’ (*109 SRs*), did the authors extract data as reported in the trials or they applied ITT de novo?	Data extracted as reported in the trials		46	42.2	0	0.0	1	2.2	3	6.5	3	6.5	11	23.9	15	32.6	8	17.4	3	6.5	2	4.3
	Intention-to-treat analysis de novo		31	28.4	1	3.2	1	3.2	6	19.4	3	9.7	6	19.4	11	35.5	2	6.5	0	0.0	1	3.2
	Combination of the aforementioned		13	12.0	1	7.7	0	0.0	3	23.1	4	30.8	1	7.7	1	7.7	3	23.1	0	0.0	0	0.0
	Unclear		19	17.4	1	5.3	0	0.0	1	5.3	3	15.8	6	31.6	4	21.1	4	21.1	0	0.0	0	0.0
Bias due to MOD was evaluated	Yes, using a specific tool		198^c^	51.2	6	3.0	8	4.0	21	10.6	15	7.6	35	17.7	52	26.3	43	21.7	10	5.1	8	4.0
	Yes, probably but results are not displayed		72	18.6	5	6.9	3	4.2	9	12.5	11	15.3	14	19.4	20	27.8	9	12.5	0	0.0	1	1.4
	No, but other domains were evaluated		25	6.5	2	8.0	1	4.0	4	16.0	4	16.0	4	16.0	6	24.0	3	12.0	0	0.0	1	4.0
	No evaluation of risk of bias		92	23.8	8	8.7	6	6.5	14	15.2	20	21.7	12	13.0	14	15.2	13	14.1	4	4.3	1	1.1
Bias due to MOD was evaluated explicitly	No explicit evaluation		181	46.8	6	3.3	8	4.4	21	11.6	15	8.3	33	18.2	46	25.4	38	21.0	8	4.4	6	3.3
	With justification of judgments		18^c^	4.6	0	0.0	0	0.0	0	0.0	0	0.0	3	17.6	6	35.3	4	23.5	2	11.8	2	11.8
	Results on bias due to MOD are not displayed		15	3.9	3	20.0	2	13.3	2	13.3	5	33.3	1	6.7	0	0.0	2	13.3	0	0.0	0	0.0
	No evaluation of bias due to MOD		25	6.5	2	8.0	1	4.0	4	16.0	4	16.0	4	16.0	6	24.0	3	12.0	0	0.0	1	4.0
	Only an overall score is provided for each trial		37	9.6	1	2.7	1	2.7	6	16.2	6	16.2	7	18.9	12	32.4	4	10.8	0	0.0	0	0.0
	Results on the risk of bias evaluation are not presented		19	4.9	1	5.3	0	0.0	1	5.3	0	0.0	5	26.3	8	42.1	3	15.8	0	0.0	1	5.3
	No evaluation of risk of bias		92	23.8	8	8.7	6	6.5	14	15.2	20	21.7	12	13.0	14	15.2	13	14.1	4	4.3	1	1.1
*Acknowledging implications of missing outcome data*																						
Among the reviews with MOD (273 SRs), the interpreted results accounted for MOD	Yes		88	32.2	4	4.5	7	8.0	16	18.2	8	9.1	17	19.3	20	22.7	10	11.4	4	4.5	2	2.3
	No		185	67.8	6	3.2	7	3.8	19	10.3	21	11.4	27	14.6	56	30.3	34	18.4	9	4.9	6	3.2
If the interpreted results accounted for MOD (88 SRs), MOD implications were reported in^b^	Abstract		46	52.3	3	6.5	2	4.3	8	17.4	3	6.5	9	19.6	11	23.9	5	11.0	3	6.5	2	4.3
	Results		26	29.5	1	3.8	2	7.7	4	15.4	2	7.7	4	15.4	8	30.8	4	15.4	1	3.8	0	0.0
	Discussion		74	84.1	4	5.4	5	6.8	13	17.6	6	8.1	16	21.6	18	24.3	7	9.5	3	4.0	2	2.7
	Conclusions		11	12.5	0	0.0	1	9.1	2	18.2	1	9.1	2	18.2	2	18.2	1	9.1	0	0.0	2	18.2
If the interpreted results accounted for MOD (88 SRs), they were discussed in the context of which NMA components^b^	NMA treatment effects		84	95.5	4	4.8	7	8.3	15	17.8	7	8.3	16	19.0	20	23.8	10	11.9	4	4.8	1	1.2
	Intervention ranking		13	14.8	1	7.7	1	7.7	2	15.4	1	7.7	3	23.0	1	7.7	1	7.7	2	15.4	1	7.7
	Heterogeneity		7	8.0	0	0.0	0	0.0	0	0.0	2	28.6	2	28.6	2	28.6	1	14.2	0	0.0	0	0.0
	Evidence consistency		3	3.4	0	0.0	1	33.3	0	0.0	0	0.0	0	0.0	2	66.7	0	0.0	0	0.0	0	0.0
What information the authors used to discuss the implications (88 SRs)^b^	Judgments from the risk of bias assessment		10	11.4	1	10.0	1	10.0	1	10.0	1	10.0	1	10.0	5	50.0	0	0.0	0	0.0	0	0.0
	The comments on the quality of evidence in SoF table		1	1.1	0	0.0	0	0.0	0	0.0	0	0.0	0	0.0	0	0.0	0	0.0	0	0.0	1	100
	Sensitivity analysis results		16	18.2	1	6.2	1	6.2	2	12.5	2	12.5	1	6.2	4	25.0	4	25.0	1	6.2	0	0.0
	Subgroup analysis on a dropout-relevant characteristic		4	4.5	0	0.0	0	0.0	0	0.0	0	0.0	3	75.0	1	25.0	0	0.0	0	0.0	0	0.0
	Meta-regression analysis using dropout as covariate		3	3.4	0	0.0	0	0.0	0	0.0	0	0.0	1	33.3	2	66.7	0	0.0	0	0.0	0	0.0
	NMA results on dropout (as a secondary outcome)		58	65.9	4	6.9	3	5.2	11	19.0	4	6.9	14	24.1	10	17.2	7	12.1	3	5.2	2	3.4
	Dropout prevalence		11	12.5	0	0.0	2	18.2	4	36.4	1	9.1	1	9.1	2	18.2	1	9.1	0	0.0	0	0.0
	Reasons for dropout		1	1.1	0	0.0	1	100	0	0.0	0	0.0	0	0.0	0	0.0	0	0.0	0	0.0	0	0.0
	The strategy used to handle MOD in primary analysis		2	2.3	0	0.0	0	0.0	0	0.0	0	0.0	0	0.0	1	50.0	1	50.0	0	0.0	0	0.0

Abbreviations: *ITT*, intention-to-treat analysis, *LOCF* last observation carried forward, *MOD* missing outcome data, *NMA* network meta-analysis, *SoF* summary of finding, *SRs* systematic reviews

^a^One review explicitly mentioned that the protocol is available, but the provided link page could not be found

^b^Multiple selections have been applied

^c^One systematic review clearly indicated that no incomplete outcome data exist in any of the included trials

### Addressing and handling of missing outcome data in protocols

Among the systematic reviews that made their protocol available (*n* = 37, 10%), the majority (*n* = 23, 62%) did not provide any strategy to address MOD in their analysis (Table 1). Five (13%) reviews planned to handle MOD as a secondary outcome, 9 (24%) planned to follow intention-to-treat analysis, whereas 2 (5.4%) planned to exclude participants with MOD or trials without complete outcome data. Of those 14 (38%) reviews that provided at least one pre-planned strategy to address MOD, only 2 (14%) did not address MOD eventually.

### Prevalence in defining missing outcome data

Overall, only 63 (16%) reviews explicitly defined either in their protocol or publication what the reviewers considered as MOD and most were published between 2011 and 2015 (Table 1). For example, Cipriani et al. [35] reported that as secondary outcome “we defined treatment discontinuation (acceptability) as the number of patients who terminated the study early for any reason during the first 8 weeks of treatment (dropouts)”. Other quotes regarding the definition of MOD can be found in Additional file 3.

Further in the definition of MOD, only 23 (6%) reviews explicitly mentioned that the LOCF approach has been already applied in the included trials (most found during 2013 and 2014). However, only 5 (1.3%) reviews clearly distinguished between LOCF and completely MOD (i.e., missing outcomes relating to participants who did not provide any measurements apart from their baseline characteristics before leaving the trial [12]). For example, Filippini et al. [36] imputed completely MOD under the ‘all missing events’ assumption while considering also the LOCF data: “we assumed that the treated- and control-group participants who dropped out and were not included in the study analysis both had the outcome (relapse or disability progression)”.

### Prevalence and handling of missing outcome data in systematic reviews

Seventy percent (*n* = 273) of the systematic reviews explicitly reported that there are MOD in the included trials with most being published between 2011 and 2015, whereas only one systematic review clearly indicated through the risk of bias assessment that no incomplete outcome data exist in any of the included trials (Table 1). Of these 273 reviews, more than half (*n* = 153, 56%) applied at least one strategy to address MOD in their analysis, especially those reviews published during 2014, whereas the remaining reviews did not describe any such strategy.

Among the 153 reviews that described at least one strategy to handle missingness, the majority (*n* = 109, 71%) used intention-to-treat analysis but only 25 (23%) reviews explicitly reported what they meant by intention-to-treat analysis (Table 1). Forty percent (*n* = 61) of the reviews considered MOD as one of the studied outcomes, whereas 4 (2.6%) reviews excluded participants with MOD from the primary analyses. The remaining reviews employed at least one sensitivity analysis (*n* = 16, 10%), followed by subgroup analysis (*n* = 6, 4%) and meta-regression analysis (*n* = 3, 2%) using a specific dropout characteristic (e.g., studies with and without complete outcome data or proportion of dropouts).

Out of 109 systematic reviews that applied intention-to-treat analysis (mainly published during 2013 and 2014), 46 (42%) extracted outcome data as reported in the trials, 31 (28%) applied intention-to-treat analysis de novo and only 25 (80%) reported specific scenarios about MOD, 13 (12%) applied a combination of ‘as reported’ and ‘*de novo* intention-to-treat analysis’, whereas 19 (17%) reviews provided no explicit information to comprehend how intention-to-treat analysis was implemented (see, Additional file 4).

### Evaluation of bias due to missing outcome data in systematic reviews

Almost half the systematic reviews (*n* = 198, 51%) assessed bias due to MOD using a specific tool to evaluate risk of bias of the trials and most were published during 2013 and 2014; only 18 (9.1%) reviews evaluated explicitly the domain by providing justification for the expressions of low, high, or unclear risk of bias (Table 1). The Cochrane risk of bias tool was the preferred tool in 147 (74%) reviews followed by the Jadad scale (*n* = 68, 34%) (see, Additional file 5). Nineteen percent (*n* = 72) of the reviews stated that bias due to MOD has been evaluated but results on that domain are not displayed at all (*n* = 15, 21%) or an overall score is provided for each trial (*n* = 37, 51%) or results on the evaluation of risk of bias are omitted altogether (*n* = 19, 26%). Other domains of risk of bias were evaluated in 25 (6.5%) systematic reviews, whereas no evaluation of risk of bias occurred in the remaining (*n* = 92, 24%).

### Acknowledgment of missing outcome data implications

Out of 273 (70%) systematic reviews that explicitly reported presence of MOD in the included trials, only 88 (32%) interpreted their results while accounting for MOD (mostly found between 2011 and 2014) (Table 1), primarily referring to the implications of MOD on NMA treatment effects (*n* = 84, 95%) using the NMA results on dropout as secondary outcome (*n* = 58, 66%) or the sensitivity analysis results (*n* = 16, 18%). The discussion section (*n* = 74, 84%) followed by the abstract (*n* = 46, 52%) were the sections in which the authors primarily discussed the implications of MOD.

### Characteristics of selected network meta-analyses

**Fig. 4:**
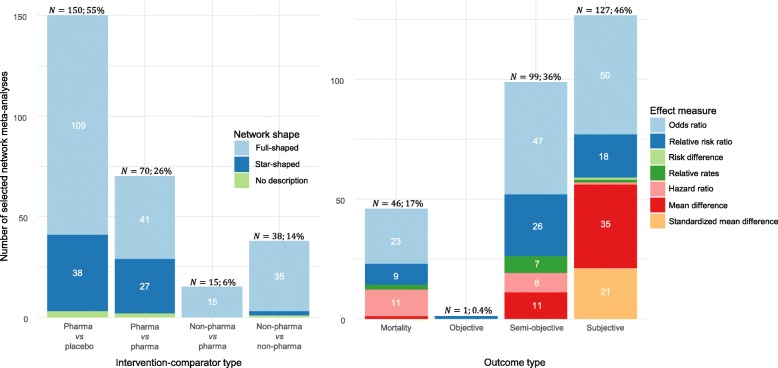
Stacked bar chart of intervention-comparator type and network shape (left) and a stacked bar chart of outcome type and effect measure in 273 selected network meta-analyses (right)

We selected one primary outcome for each systematic review that explicitly or implicitly denoted the presence of MOD in the included trials (*n* = 273). A median of 18 (IQR: 11–36) RCTs were included in the 273 selected NMAs that assessed a median of 6 (IQR: 4–9) interventions. The majority of the NMAs referred to a full network (*n* = 200, 73%), compared pharmacological interventions against placebo (*n* = 150, 55%) and investigated a subjective primary outcome (*n* = 127, 46%). Binary outcomes were more prevalent (*n* = 175, 64%) with odds ratio being the most frequently used effect measure (*n* = 120, 68%). Figure 4 illustrates the distribution of the network shape by intervention-comparator type (left) and the distribution of effect measure by outcome type (right) in 273 selected NMAs.

### Addressing missing outcome data in selected primary outcomes

A median of 5 RCTs (IQR: 3–12) handled intermediate missing outcomes using the LOCF approach (11 NMAs). Only 113 (41%) NMAs explicitly mentioned the analysis plan adopted (Table 2), and most were published during 2012 and 2014, with intention-to-treat analysis being the most prevalent analysis plan (*n* = 105, 93%). While judging the actual method of primary analysis rather than the reported method, we concurred only with 14 (12%) out of 113 NMAs (see, Additional file 6). We were able to judge the actual method applied in the primary analysis in 95 (35%) out of 273 NMAs that were published mostly between 2011 and 2014; we judged the great majority of NMAs to have analyzed the data as reported in the included trials (*n* = 57, 60%). Among the 16 NMAs that were judged to have imputed MOD in the primary analysis, 8 (50%) assumed all MOD to be non-events, 1 (6.2%) assumed all MOD to be events and 7 (44%) provided no information on the missingness scenarios. For the scenarios selected to impute MOD (9 NMAs), the reviewers considered no trial information on the reasons for dropout.

**Table 2:** Characteristics of 273 selected network meta-analyses

Characteristic	Levels		Total		2009 (*n* = 10)		2010 (*n* = 14)		2011 (*n* = 35)		2012 (*n* = 29)		2013 (*n* = 44)		2014 (*n* = 76)		2015 (*n* = 44)		2016 (*n* = 13)		2017 (*n* = 8)	
			n	%																		
*Addressing and handling missing outcome data*																						
Reported choice of primary analysis in the context of MOD	Intention-to-treat analysis		105	38.5	3	2.9	2	1.9	13	12.4	12	11.4	24	22.9	29	27.6	16	15.2	3	2.9	3	2.9
	Modified intention-to-treat analysis		4	1.5	0	0.0	0	0.0	0	0.0	1	25.0	0	0.0	2	50.0	1	25.0	0	0.0	0	0.0
	Per-protocol analysis		4	1.5	0	0.0	0	0.0	1	25.0	1	25.0	0	0.0	1	25.0	0	0.0	0	0.0	1	25.0
	Not mentioned		160	58.6	7	4.4	12	7.5	21	13.1	15	9.4	20	12.5	44	27.5	27	16.9	10	6.2	4	2.5
We judged the actual method of primary analysis rather than the reported method to be possibly	Available case analysis with LOCF		6	2.2	0	0.0	2	33.3	2	33.3	1	16.7	0	0.0	0	0.0	1	16.7	0	0.0	0	0.0
	Available case analysis without LOCF		15	5.5	0	0.0	3	20.0	3	20.0	1	6.7	2	13.3	3	20.0	2	13.3	1	6.7	0	0.0
	Imputation method	with LOCF	2	0.7	0	0.0	0	0.0	1	50.0	0	0.0	1	50.0	0	0.0	0	0.0	0	0.0	0	0.0
		without LOCF	14	5.1	1	7.1	0	0.0	3	21.4	3	21.4	2	14.3	2	14.3	3	21.4	0	0.0	0	0.0
	Modified intention-to-treat analysis		1	0.4	0	0.0	0	0.0	0	0.0	1	100	0	0.0	0	0.0	0	0.0	0	0.0	0	0.0
	Outcome analyzed as reported in the included trials		57	20.9	3	5.3	5	8.8	10	17.5	7	12.3	11	19.3	12	21.1	6	10.5	3	5.3	0	0.0
	Unclear		178	65.2	6	3.4	4	2.2	16	9.0	16	9.0	28	15.7	59	33.1	32	18.0	9	5.1	8	4.5
If applicable, which imputation method was judged to be used (*16 NMAs*)	All MOD as non-events		8	50.0	1	12.5	0	0.0	2	25.0	1	12.5	1	12.5	1	12.5	2	25.0	0	0.0	0	0.0
	All MOD as events		1	6.2	0	0.0	0	0.0	0	0.0	0	0.0	1	100	0	0.0	0	0.0	0	0.0	0	0.0
	Not mentioned		7	43.8	0	0.0	0	0.0	2	28.6	2	28.6	1	14.3	1	14.3	1	14.3	0	0.0	0	0.0
If imputation of MOD was employed (*16 NMAs*), the scenarios considered in the network are	Common across trials and interventions		9	56.3	1	11.1	0	0.0	2	22.2	1	11.1	2	22.2	1	11.1	2	22.2	0	0.0	0	0.0
	Unclear		7	43.7	0	0.0	0	0.0	2	28.6	2	28.6	1	14.3	1	14.3	1	14.3	0	0.0	0	0.0
For the scenarios selected to impute MOD (*16 NMAs*), the reviewer considered relevant trial information on the reasons for dropout	No		9	56.3	1	11.1	0	0.0	2	22.2	1	11.1	2	22.2	1	11.1	2	22.2	0	0.0	0	0.0
	Unclear		7	43.7	0	0.0	0	0.0	2	28.6	2	28.6	1	14.3	1	14.3	1	14.3	0	0.0	0	0.0
*Sensitivity analysis on missing outcome data*																						
Strategy reported to handle MOD in a sensitivity analysis^a^	Trial exclusion		7	2.6	2	28.6	0	0.0	1	14.3	0	0.0	1	14.3	2	28.6	1	14.3	0	0.0	0	0.0
	Available case analysis		5	1.8	0	0.0	1	20.0	1	20.0	0	0.0	0	0.0	2	40.0	1	20.0	0	0.0	0	0.0
	Imputation		3	1.1	0	0.0	1	33.3	0	0.0	1	33.3	0	0.0	0	0.0	1	33.3	0	0.0	0	0.0
	Modified intention-to-treat analysis with other definitions		2	0.7	0	0.0	0	0.0	0	0.0	1	50.0	0	0.0	0	0.0	1	50.0	0	0.0	0	0.0
	No sensitivity analysis		257	94.1	8	3.1	13	5.1	33	12.8	27	10.5	43	16.7	72	28.0	40	15.6	13	5.1	8	3.1
If sensitivity analysis applied (16 NMAs), the authors reported any changes in the inferences after sensitivity analysis	Yes, it was reported that no changes were detected		13	81.2	1	7.7	0	0.0	2	15.4	2	15.4	1	7.7	3	23.1	4	30.8	0	0.0	0	0.0
	Yes, it was reported that changes were detected		2	12.5	0	0.0	1	50.0	0	0.0	0	0.0	0	0.0	1	50.0	0	0.0	0	0.0	0	0.0
	Not mentioned		1	6.3	1	100	0	0.0	0	0.0	0	0.0	0	0.0	0	0.0	0	0.0	0	0.0	0	0.0
Explanation given by the reviewers to support their strategy to handle MOD in primary and sensitivity analysis for the selected outcome	Yes, they provided an explanation		12	4.4	2	16.7	1	8.3	0	0.0	2	16.7	1	8.3	4	33.3	1	8.3	0	0.0	1	8.3
	No explanation is given		112	41.0	2	1.8	1	0.9	16	14.3	12	10.7	24	21.4	32	28.6	18	16.1	4	3.6	3	2.7
	Not applicable		149^b^	54.6	6	4.0	12	8.1	19	12.8	15	10.1	19	12.8	40	26.8	25	16.8	9	6.0	4	2.7
*Availability of primary and missing outcome data for extraction*																						
Provided numerical information on MOD for post-hoc analysis	Extractable (i.e., at arm- or trial-level of every trial)		55	20.1	4	7.3	5	9.1	13	23.6	4	7.3	8	14.5	12	21.8	7	12.7	2	3.6	0	0.0
	Not usable information (e.g., at intervention level)		27	9.9	0	0.0	1	3.7	4	14.8	4	14.8	5	18.5	8	29.6	2	7.4	1	3.7	2	7.4
	No numerical information on MOD		191	70.0	6	3.1	8	4.2	18	9.4	21	11.0	31	16.2	56	29.3	35	18.3	10	5.2	6	3.1
Data extraction for the selected primary outcome is judged to be overall	Extractable^c^		39	14.3	3	7.7	3	7.7	8	20.5	3	7.7	8	20.5	8	20.5	5	12.8	1	2.6	0	0.0
	Contact authors for raw outcome data^d^		8	2.9	1	12.5	2	25.0	2	25.0	0	0.0	0	0.0	1	12.5	2	25.0	0	0.0	0	0.0
	Contact authors for raw MOD^e^		4	1.5	0	0.0	0	0.0	1	25.0	0	0.0	0	0.0	3	75.0	0	0.0	0	0.0	0	0.0
	Not extractable^f^		222	81.3	6	2.7	9	4.1	24	10.8	26	11.7	36	16.2	64	28.8	37	16.7	12	5.4	8	3.6

Abbreviations: *LOCF* last observation carried forward, *MOD* missing outcome data, *NMA* network meta-analysis

^a^Multiple selections have been applied

^b^120 systematic reviews did not mention any strategy to address missing outcome data and 29 systematic reviews addressed missing outcome data only as a secondary outcome

^c^Both primary and missing outcome data are provided for each arm in every trial

^d^No raw primary outcome data are provided but missing outcome data are provided for each arm of every (or some) trial(s)

^e^Primary outcome data are provided at arm-level for every trial but missing outcome data are provided at trial-level

^f^Primary outcome data are provided at contrast-level in each trial with trial- or intervention-level or no information at all on missing outcome data; primary outcome data are provided at arm-level for every trial with intervention-level or no information at all on missing outcome data; no raw primary outcome data are provided as well as no information on missing outcome data

### Sensitivity analysis on missing outcome data for selected primary outcomes

Most NMAs were not re-analyzed in a sensitivity analysis (*n* = 257, 94%) to investigate the implications of MOD on the primary analysis results further (Table 2). Out of 16 NMAs with at least one sensitivity analysis (most published during 2014 and 2015), only 2 (12%) reported that changes were detected when compared with the primary analysis results. Specifically, Delahoy et al. [37] reported that “Both sensitivity analyses [one after excluding missing outcome data and one after imputing all missing cases as failures] supported the findings of the base-case analysis, although statistical significance was not demonstrated”, whereas Cui et al. [38] stated that “[After using per-protocol instead of intention-to-treat analysis] two outcomes for apixaban and dabigatran in preventing total VTE were significantly affected”. Overall, for 12 (4.4%) out of 273 NMAs there was an explicit explanation to support the strategy applied to handle MOD in primary and sensitivity analysis (see, Additional file 7) and the majority was published during 2014.

### Availability of primary and missing outcome data for extraction

Only 55 (20%) out of 273 NMAs provided extractable MOD at arm- or trial-level for every included trial (Table 2) with most being published between 2011 and 2015. In 27 (10%) NMAs, MOD were reported at intervention-level, whereas the remaining 191 (70%) NMAs provided no numerical information on MOD. Overall, 39 (14%) NMAs provided necessary numerical information for each arm in every trial (published mostly between 2011 and 2014) in order to enable extraction of both primary and missing outcome data.

## Discussion

In this systematic survey, we reviewed a total of 387 systematic reviews with NMA on at least three interventions. Only a fraction described in their protocol a strategy to address MOD or provided a definition of MOD. Overall, there was insufficient information in most systematic reviews with NMA to judge the actual strategy applied to handle MOD. Only a small fraction of NMAs were re-analyzed in at least one sensitivity analysis for MOD and an even smaller number explicitly justified the strategy applied in primary and/or sensitivity analysis to address MOD.

This is the first systematic survey that provides comprehensive evidence about the reporting and handling of MOD in networks of interventions. It suggests that planning, reporting and conduct of analyzing the impact of MOD in NMA results is poor and will benefit from related guidance.

We believe that this work is representative because it vastly expands our previous work in mental health Cochrane reviews [12]. We evaluated twice the systematic reviews covering a wide range of several health specialties [12]. Another strength of our study includes searching a multitude of sources that include 4 previously published collections of NMAs and an update of the original searches using an extensive previously published search strategy [32].

The weaknesses of our study are the lack of guidance for handling MOD in NMA itself. Without this guidance, the authors of the systematic reviews could not follow proper reporting and analysis standards. The overall poor quality of the reporting of the eligible systematic reviews raised a number of challenges for our work. First, only a fraction of reviews provided numerical information on MOD (82 out of 387), whereas 70% of the reviews with potential MOD in the included trials provided only implicit information on MOD and primarily through the assessment of bias due to MOD. As a result, we were not able to appraise the precise extent of MOD in the reviews and by extent, in each clinical field. Second, we were not able to provide an actual estimation of the use of the LOCF approach in the included studies since only 11 networks made available such information (again, mostly through the assessment of bias due to MOD). Therefore, we might have underestimated the extent of the LOCF approach in systematic reviews with NMA. Third, we strived to judge as objectively as possible the actual method applied in the primary analysis by developing a classification of judgments which was then applied in all eligible reviews. However, considering also the particular nature of this task this judgment remained subjective. Finally, we did not attempt to contact the authors of the reviews that mentioned a protocol but did not make the protocol publicly available (1 out of 5 reviews). It is unlikely, though, that these protocols would have addressed MOD more explicitly.

The results of our study are comparable with our previous work [12] focusing on Cochrane reviews with two interventions in mental health and with the work of Akl et al. [13] who investigated Cochrane and non-Cochrane reviews with focus on binary outcome data. We observed that only a small fraction of reviews provided a clear definition for MOD or distinguished between LOCF and *completely* MOD. Furthermore, most of the reviews addressed MOD either as a secondary outcome or by applying an imputation strategy for the purpose of complying with the intention-to-treat principle. Sensitivity analysis was among the least prevalent methods to address MOD, whereas the interpreted results were hardly discussed in the context of MOD. Akl et al. [13] encountered also a low prevalence in the reporting and handling of MOD. Exclusion of missing participants from the analysis and application of sensitivity analysis were the most and least prevalent methods, respectively. Assessment of bias due to MOD was primarily a characteristic of Cochrane but not of other reviews.

Nevertheless, contrary to our previous work [12], we were not able to judge the actual method applied in the majority of the selected NMAs. This difficulty primarily stems from insufficient information about important components that are necessary to derive the actual method of primary analysis explicitly or implicitly. Additional file 6 shows examples of systematic reviews with explicit, implicit and unclear judgments alongside the justification of our judgments.

Our systematic survey revealed that systematic reviews with NMA rarely develop protocols prior to their conduct, while those that make protocols available in public, tend not to provide a strategy to address MOD. A carefully planned protocol should include explicit information on collecting MOD for each arm in every trial, on the methodology considered (e.g., imputation or likelihood-based methods) and assumptions about missing cases (source and distribution across trials and interventions). Ideally, the reviewers should also attempt to distinguish between completely MOD and LOCF data and record the trials with information on LOCF. In that way, the actual extent of MOD and their implications on the conclusions can be appraised more accurately.

We found that many systematic reviews favored intention-to-treat analysis but only a handful of those provided further information on the technique and assumptions made about the missing cases. Indeed, a great majority of these reviews merely mentioned that, ‘where possible, data were extracted under the intention-to-treat principle as reported in the included trials’. However, the reviewers made no efforts to evaluate the validity of the analysis claimed in these trials, as they seem to have taken at face value the reported results. The latter increases the risk of misconducting an intention-to-treat analysis in the review, since intention-to-treat analysis is notorious for being described and implemented inadequately in trials [39]. Without possessing at least the number analyzed out of the number randomized in the included trials, the reported analysis in the selected outcome may not be following the genuine intention-to-treat principle.

Without information available on post-withdrawal outcomes, MOD are eventually analyzed using untestable assumptions [40]. Therefore, a set of clinically plausible sensitivity analyses needs to be selected to explore the deviations from the assumption made in the primary analysis and by extent, the robustness of the inferences [40, 41]. Nevertheless, our survey demonstrated that sensitivity analyses in the context of MOD are very rarely planned in systematic reviews with NMAs (*n* = 16, 4.1%). We previously found that only 16% of the eligible meta-analyses applied at least one sensitivity analysis, whereas only half communicated their findings while considering the results of the sensitivity analyses. It is possible that more careful protocol development and peer review of Cochrane reviews provided some protection against this shortcoming. Gamble and Hollis [17] first shed light on the methods used in Cochrane reviews with meta-analyses to address MOD, and they found that half of the systematic reviews planned and performed sensitivity analysis, whereas interpretation of the sensitivity analyses was either unclear or omitted, overall.

## Conclusions

Our comprehensive empirical systematic survey indicates that the quality of reporting and handling MOD in systematic reviews with NMAs is inadequate. Despite the published literature on MOD, reviewers remain either unaware of the presence and importance of MOD or unable to plan to deal with MOD in systematic reviews of multiple interventions. In addition, the poor handling of MOD attests to limited knowledge of the reviewers regarding the existing relevant methodology. Therefore, further methods development, reporting guidelines and education amongst reviewers is deemed necessary to allow addressing the implications on the quality of the inferences delivered to the healthcare system. To ensure a transparent, detail-oriented reporting and methodologically appropriate management of MOD in systematic reviews, peer reviewers and journal editors may benefit from training as well.

## Additional files

Additional file 1: **Appendix A.** List of excluded articles by reason for exclusion. (DOC 223 kb)

Additional file 2: **Appendix B.** List of included articles by year of publication. (DOC 254 kb)

Additional file 3: **Appendix C.** List of verbatim definitions of missing outcome data. (DOCX 23 kb)

Additional file 4: **Appendix D.** Description of intention-to-treat analyses according to reports in the reviews. (DOCX 35 kb)

Additional file 5: **Appendix E.** Tools used for the risk of bias assessment in 387 systematic reviews. (DOCX 49 kb)

Additional file 6: **Appendix F.** Examples of systematic reviews with explicit, implicit and unclear judgments. (DOCX 57 kb)

Additional file 7: **Appendix G.** Explanations to support the strategy applied in primary and sensitivity analysis. (DOCX 22 kb)

## Abbreviations

GRADE: Grading of Recommendations, Assessment, Development and Evaluation
IMOR: Informative missingness odds ratio
LOCF: Last-observation-carried-forward
MOD: Missing outcome data
NMA: Network meta-analysis
RCT: Randomized controlled trials
