# Supplementary material for: A systematic survey shows that reporting and handling of missing outcome data in networks of interventions is poor
Source: BMC Med Res Methodol. 2018 Oct 24;18:115. doi: 10.1186/s12874-018-0576-9 (PMC6201503; doi:10.1186/s12874-018-0576-9)
Supplement: Supplementary file 1 — Appendix A. List of excluded articles by reason for exclusion. (DOC 223 kb) [file 12874_2018_576_MOESM1_ESM.doc]

**Appendix A. List of excluded articles by reason of exclusion**

| **Articles in Chinese, Hungarian and Russian language** |
| --- |

1. Avxent'eva MV, Cherniavskiĭ AM, Piadushkina EA. [Economic Evaluation of Riociguat in Patients with Inoperable or Residual Chronic Thromboembolic Pulmonary Hypertension]. [Article in Russian] Kardiologiia. 2015;55(3):33-40.
2. Brodszky V. [Effectiveness of biological treatments based on ACR70 response in rheumatoid arthritis: indirect comparison and meta-regression using Bayes-model]. [Article in Hungarian] Orv Hetil. 2011;152(23):919-28. doi: 10.1556/OH.2011.29138.
3. Ni RH, Tang HL, Zhai SD, Li ZL. [Multiple treatments for infantile rotavirus enteritis: a network meta-analysis]. World Chinese Journal of Digestology. 2012; 20(5): 438-443.
4. Zhang HN, Shao R. A Network Meta-Analysis on the Influence of Antihypertensive Agents on New-onset Diabetes. Chinese Pharmaceutical Journal. 2013;11.

| **Disconnected networks** |
| --- |

1. Qi P, Chen M, Zhang L, Song R, He Z, Wang Z. A Meta-Analysis and Indirect Comparison of Endothelin A Receptor Antagonist for Castration-Resistant Prostate Cancer. PLoS One. 2015;10(7):e0133803. <http://dx.plos.org/10.1371/journal.pone.0133803>.

2. Wolff R, Ryder S, Bossi A, Briganti A, Crook J, Henry A, et al. A systematic review of randomised controlled trials of radiotherapy for localised prostate cancer. Eur J Cancer. 2015;51(16):2345–67. <http://dx.doi.org/10.1016/j.ejca.2015.07.019>.

| **Dropout as primary outcome** |
| --- |

1. Desai R, Hansen R, Rao J, Wilkins T, Harden E, Yuen A, et al. Mixed treatment comparison of the treatment discontinuations of biologic disease-modifying antirheumatic drugs in adults with rheumatoid arthritis. Ann Pharmacother. 2012;46(11):1491–505. <http://journals.sagepub.com/doi/10.1345/aph.1R203>.
2. Edwards S, Smith C. Tolerability of atypical antipsychotics in the treatment of adults with schizophrenia or bipolar disorder: A mixed treatment comparison of randomized controlled trials. Clin Ther. 2009;31(Pt 1):1345–59.<http://dx.doi.org/10.1016/j.clinthera.2009.07.004>.
3. Lin P, Chen H, Wang Y, Tu Y. Primary molar pulpotomy: A systematic review and network meta-analysis. J Dent. 2014;42(9):1060–77. <http://dx.doi.org/10.1016/j.jdent.2014.02.001>.
4. Mavranezouli I, Meader N, Cape J, Kendall T. The cost effectiveness of pharmacological treatments for generalized anxiety disorder. Pharmacoeconomics. 2013;31(4):317–33.
5. Meader N. A comparison of methadone, buprenorphine and alpha_2_ adrenergic agonists for opioid detoxification: a mixed treatment comparison meta-analysis. Drug Alcohol Depend. 2010;108(1–2):110–4. <http://dx.doi.org/10.1016/j.drugalcdep.2009.12.008>
6. Mills E, Lester R, Thorlund K, Lorenzi M, Muldoon K, Kanters S, et al. Interventions to promote adherence to antiretroviral therapy in Africa: a network meta-analysis. Lancet HIV. 2014;1(3):104–11.
7. Naci H, Brugts J, Ades T. Comparative tolerability and harms of individual statins: a study-level network meta-analysis of 246 955 participants from 135 randomized, controlled trials. Circ Cardiovasc Qual Outcomes. 2013;6(4):390–9.
8. Singh J, Wells G, Christensen R, Tanjong Ghogomu E, Maxwell L, MacDonald J, et al. Adverse effects of biologics: a network meta-analysis and Cochrane overview. Cochrane Database Syst Rev. 2011;(2):CD008794. <http://doi.wiley.com/10.1002/14651858.CD008794.pub2>.
9. Zaccara G, Giovannelli F, Maratea D, Fadda V, Verrotti A. Neurological adverse events of new generation sodium blocker antiepileptic drugs. Meta-analysis of randomized, double-blinded studies with eslicarbazepine acetate, lacosamide and oxcarbazepine. Seizure. 2013;22(7):528–36. <http://dx.doi.org/10.1016/j.seizure.2013.03.016>.

| **Letter to the Editor, Editorial, commentary** |
| --- |

1. Belsey J. Appropriate use of information in therapeutic decision-making: reflections on indirect comparisons. Curr Med Res Opin. 2015;31(2):343–6.
2. Brasher P. Multiple treatment comparison analysis: If A is better than B, and B is better than C, is A better than C? Can J Anaesth. 2013;60(3):227–9.
3. Caldwell D. An overview of conducting systematic reviews with network meta-analysis. Syst Rev. 2014;3:109.
4. Catalá-López F. Renoprotective effects of renin-angiotensin-aldosterone system blockers in type 2 diabetes: demystifying multiple treatment comparisons in a network meta-analysis. Diabetologia. 2012;55(9):2547–8.
5. Cipriani A, Geddes J. Placebo for depression: we need to improve the quality of scientific information but also reject too simplistic approaches or ideological nihilism. BMC Med. 2014;12(1):105. <http://bmcmedicine.biomedcentral.com/articles/10.1186/1741-7015-12-105>
6. de Jongh A, de Roos C, Bicanic I. Integrating fragmented evidence by network meta-analysis: relative effectiveness of psychological interventions for adults with post-traumatic stress disorder’. Psychol Med. 2014;44(15):3357–8.
7. Maratea D, Fadda V, Trippoli S, Messori A. Prevention of venous thromboembolism after major orthopedic surgery: indirect comparison of three new oral anticoagulants. J Thromb Haemost. 2011;9(9):1868–70.
8. Martin-Broto J. Indirect comparisons in cost-effectiveness analysis: are we being naïve? Clin Transl Oncol. 2015;17(1):85–6.
9. Messori A. Practical application of statistical models aimed at assessing bioequivalence through network meta-analysis. Eur J Clin Pharmacol. 2014;70(12):1527–8.
10. Messori A, Fadda V, Maratea D, Trippoli S. New endovascular devices for acute ischemic stroke: summarizing evidence by multiple treatment comparison meta-analysis. Ann Vasc Surg. 2013;27(3):395–6. <http://dx.doi.org/10.1016/j.avsg.2012.10.010>.
11. Migliore A, Broccoli S, Bizzi E, Laganà B. Indirect comparison of etanercept, infliximab, and adalumimab for psoriatic arthritis: mixed treatment comparison using placebo as common comparator. Clin Rheumatol. 2012;31(1):193–4.
12. Naci H, Van Valkenhoef G, Higgins J, Fleurence R, Ades A. Evidence-based prescribing: combining network meta-analysis with multicriteria decision analysis to choose among multiple drugs. Circ Cardiovasc Qual Outcomes. 2014;7(5):787–92.
13. Nixon R, Capkun-Niggli G, Bergvall N, Dias S. Re: Hutchinson M, Fox RJ, Havrdova E, et al. Efficacy and safety of BG-12 (dimethyl fumarate) and other disease modifying therapies for the treatment of relapsing-remitting multiple sclerosis: a systematic review and mixed treatment comparison. Curr Med Res Opin. 2015;31(1):51–2.
14. Ortega A, Fraga M, Alegre-Del-Rey E, Puigventós-Latorre F, Porta A, Ventayol P, et al. A checklist for critical appraisal of indirect comparisons. Int J Clin Pract. 2014;68(10):1181–9.
15. Pazianas M, Abrahamsen B. Comment on Tadrous et al.: comparative gastrointestinal safety of bisphosphonates in primary osteoporosis: a network meta-analysis. Osteoporos Int. 2014;25(11):2669.
16. Pipe A. Network meta-analysis demonstrates the safety of pharmacotherapy for smoking cessation in cardiovascular patients. Evid Based Med. 2014;19(5):193. <http://ebm.bmj.com/content/19/5/193.short>.
17. Puig L. On clinical thresholds, clinical equivalents and indirect comparisons of biological treatments for moderate-to-severe psoriasis. J Clin Pharm Ther. 2015;40(2):131–4.
18. Tadrous M, Wong L, Mamdani M, Juurlink D, Krahn M, Lévesque L, et al. Comparative gastrointestinal safety of bisphosphonates in primary osteoporosis: a network meta-analysis-reply to Pazianas and Abrahamsen. Osteoporos Int. 2014;25(4):2671–2.
19. Tonin F, Piazza T, Wiens A, Fernandez-Llimos F, Pontarolo R. Adverse events and treatment failure leading to discontinuation of recently approved antipsychotic drugs in schizophrenia: A network meta-analysis. Schizophr Res. 2015;169(1–3):483–5. <http://dx.doi.org/10.1016/j.schres.2015.09.019>.
20. Tubaro A, De Nunzio C. Words of wisdom. Re: Comparative efficacy and safety of medical treatments for the management of overactive bladder: a systematic literature review and mixed treatment comparison. Eur Urol. 2014;65(6):1220–1. <http://dx.doi.org/10.1016/j.eururo.2014.02.027>.
21. Wattar B, Zamora J, Khan K. Informing treatment decisions through meta-analysis: to network or not? Evid Based Med. 2017;22(1):12–5.

| **Literature review on classical or network meta-analysis** |
| --- |

1. Biondi-Zoccai G, Abbate A, Benedetto U, Palmerini T, D’Ascenzo F, Frati G. Network meta-analysis for evidence synthesis: what is it and why is it posed to dominate cardiovascular decision making? Int J Cardiol. 2015;182:309–14. <http://dx.doi.org/10.1016/j.ijcard.2015.01.023>.
2. Donegan S, Williamson P, D’Alessandro U, Tudur Smith C. Assessing key assumptions of network meta-analysis: a review of methods. Res Synth Methods. 2013;4(4):291–323.
3. Donovan L, Wakefield C, Russell V, Cohn R. Hospital-based bereavement services following the death of a child: a mixed study review. Palliat Med. 2015;29(3):193–210. <http://journals.sagepub.com/doi/10.1177/0269216314556851>.
4. Kim H, Gurrin L, Ademi Z, Liew D. Overview of methods for comparing the efficacies of drugs in the absence of head-to-head clinical trial data. Br J Clin Pharmacol. 2014;77(1):116–21.
5. Kleijnen S, Fathallah M, Van Der Linden M, Vancraeynest P, Dahmani B, Timoney A, et al. Can a Joint Assessment Provide Relevant Information for National/Local Relative Effectiveness Assessments? An In-Depth Comparison of Pazopanib Assessments. Value Health. 2015;18(5):663–72. <http://dx.doi.org/10.1016/j.jval.2015.03.1790>.
6. Laws A, Kendall R, Hawkins N. A comparison of national guidelines for network meta-analysis. Value Health. 2014;17(5):642–54. <http://dx.doi.org/10.1016/j.jval.2014.06.001>.
7. Lee A. Review of mixed treatment comparisons in published systematic reviews shows marked increase since 2009. J Clin Epidemiol. 2014;67(2):138–43. <http://dx.doi.org/10.1016/j.jclinepi.2013.07.014>.
8. Palmerini T, Biondi-Zoccai G, Stone G. Stent selection to minimize the risk of stent thrombosis. Curr Opin Cardiol. 2014;29(6):578–85.
9. Tan S, Bujkiewicz S, Sutton A, Dequen P, Cooper N. Presentational approaches used in the UK for reporting evidence synthesis using indirect and mixed treatment comparisons. J Health Serv Res Policy. 2013;18(4):224–32.
10. Thorlund K, Druyts E, Aviña-Zubieta J, Wu P, Mills E. Why the findings of published multiple treatment comparison meta-analyses of biologic treatments for rheumatoid arthritis are different: an overview of recurrent methodological shortcomings. Ann Rheum Dis. 2013;72(9):1524–35. <http://ard.bmj.com/lookup/doi/10.1136/annrheumdis-2012-201574>.
11. Veroniki A, Soobiah C, Tricco A, Elliott M, Straus S. Methods and characteristics of published network meta-analyses using individual patient data: protocol for a scoping review. BMJ Open. 2015;5(4):e007103. <http://www.pubmedcentral.nih.gov/articlerender.fcgi?artid=4420933&tool=pmcentrez&rendertype=abstract>.
12. Zaccara G, Giovannelli F, Bell G, Sander J. Network meta-analyses of antiepileptic drug efficacy and tolerability in drug-resistant focal epilepsies: a clinical perspective. Eur J Clin Pharmacol. 2014;70(6):647–54.

| **Meta-epidemiological and empirical studies** |
| --- |

1. Caldwell D, Dias S, Welton N. Extending Treatment Networks in Health Technology Assessment: How Far Should We Go? Value Health. 2015;18(5):673–81.
2. Dequen P, Sutton A, Scott D, Abrams K. Searching for indirect evidence and extending the network of studies for network meta-analysis: case study in venous thromboembolic events prevention following elective total knee replacement surgery. Value Health. 2014;17(4):416–23. <http://dx.doi.org/10.1016/j.jval.2014.02.013>.
3. Fountoulakis K, Veroniki A, Siamouli M, Möller H. No role for initial severity on the efficacy of antidepressants: results of a multi-meta-analysis. Ann Gen Psychiatry. 2013;12(1):26. <http://annals-general-psychiatry.biomedcentral.com/articles/10.1186/1744-859X-12-26>.
4. Llorca P, Lançon C, Brignone M, Painchault C, Rive B, Toumi M, et al. Can we well assess the relative efficacy and tolerability of a new drug versus others at the time of marketing authorization using mixed treatment comparisons? A detailed illustration with escitalopram. J Mark access Heal policy. 2015;3.
5. Naci H, Dias S, Ades A. Industry sponsorship bias in research findings: a network meta-analysis of LDL cholesterol reduction in randomised trials of statins. BMJ. 2014;349:g5741. <http://www.bmj.com/cgi/doi/10.1136/bmj.g5741>.
6. Naci H, Ioannidis J. Comparative effectiveness of exercise and drug interventions on mortality outcomes: metaepidemiological study. BMJ. 2013;347:f5577. Available from: <http://www.bmj.com/cgi/doi/10.1136/bmj.f5577>.
7. Naci H, Ioannidis J. Comparative effectiveness of exercise and drug interventions on mortality outcomes: metaepidemiological study. Br J Sports Med. 2015;49(21):1414–22.
8. Naudet F, Millet B, Charlier P, Reymann J, Maria A, Falissard B. Which placebo to cure depression? A thought-provoking network meta-analysis. BMC Med. 2013;11:230.
9. Rheims S, Perucca E, Cucherat M, Ryvlin P. Factors determining response to antiepileptic drugs in randomized controlled trials. A systematic review and meta-analysis. Epilepsia. 2011;52(2):219–33.
10. Rücker G, Schwarzer G. Reduce dimension or reduce weights? Comparing two approaches to multi-arm studies in network meta-analysis. Stat Med. 2014;33(25):4353–69.
11. Trinquart L, Abbé A, Ravaud P. Impact of reporting bias in network meta-analysis of antidepressant placebo-controlled trials. PLoS One. 2012;7(4):e35219.
12. Zafari Z, Thorlund K, FitzGerald JM, Marra CA, Sadatsafavi M. Network vs. pairwise meta-analyses: a case study of the impact of an evidence-synthesis paradigm on value of information outcomes. Pharmacoeconomics. 2014;32(10):995-1004. doi: 10.1007/s40273-014-0179-1.

| **Methodological articles on classical and network meta-analysis** |
| --- |

1. Achana F, Cooper N, Bujkiewicz S, Hubbard S, Kendrick D, Jones D, et al. Network meta-analysis of multiple outcome measures accounting for borrowing of information across outcomes. BMC Med Res Methodol. 2014;14(1):92. <http://bmcmedresmethodol.biomedcentral.com/articles/10.1186/1471-2288-14-92>.
2. Ades A, Mavranezouli I, Dias S, Welton N, Whittington C, Kendall T. Network meta-analysis with competing risk outcomes. Value Health. 2010;13(8):976–83. <http://dx.doi.org/10.1111/j.1524-4733.2010.00784.x>.
3. Caldwell D, Welton N, Ades A. Mixed treatment comparison analysis provides internally coherent treatment effect estimates based on overviews of reviews and can reveal inconsistency. J Clin Epidemiol. 2010;63(8):875–82. <http://dx.doi.org/10.1016/j.jclinepi.2009.08.025>.
4. Chambers J, Winn A, Zhong Y, Olchanski N, Cangelosi M. Potential role of network meta-analysis in value-based insurance design. Am J Manag Care. 2014;20(8):641–8.
5. Cope S, Zhang J, Saletan S, Smiechowski B, Jansen J, Schmid P. A process for assessing the feasibility of a network meta-analysis: a case study of everolimus in combination with hormonal therapy versus chemotherapy for advanced breast cancer. BMC Med. 2014;12(1):93. <http://bmcmedicine.biomedcentral.com/articles/10.1186/1741-7015-12-93>.
6. Dakin H, Welton N, Ades A, Collins S, Orme M, Kelly S. Mixed treatment comparison of repeated measurements of a continuous endpoint: an example using topical treatments for primary open-angle glaucoma and ocular hypertension. Stat Med. 2011;30(20):2511–35.
7. Delea T, Amdahl J, Chit A, Amonkar M. Cost-effectiveness of lapatinib plus letrozole in her2-positive, hormone receptor-positive metastatic breast cancer in Canada. Curr Oncol. 2013;20(5):e371-387.
8. DeSantis S, Zhu H. A Bayesian mixed-treatment comparison meta-analysis of treatments for alcohol dependence and implications for planning future trials. Med Decis Mak. 2014;34(7):899–910. <http://www.ncbi.nlm.nih.gov/pubmed/24935915>.
9. Dias S, Welton N, Caldwell D, Ades A. Checking consistency in mixed treatment comparison meta-analysis. Stat Med. 2010;29(7–8):932–44.
10. Donegan S, Williamson P, D’Alessandro U, Garner P, Smith C. Combining individual patient data and aggregate data in mixed treatment comparison meta-analysis: individual patient data may be beneficial if only for a subset of trials. Stat Med. 2013;32(6):914–30.
11. Donegan S, Williamson P, D’Alessandro U, Tudur Smith C. Assessing the consistency assumption by exploring treatment by covariate interactions in mixed treatment comparison meta-analysis: Individual patient-level covariates versus aggregate trial-level covariates. Stat Med. 2012;31(29):3840–57.
12. Dorman E, Kansal AR, Sarda S. The budget impact of introducing delayed-release dimethyl fumarate for treatment of relapse-remitting multiple sclerosis in Canada. J Med Econ. 2015;18(12):1085-91. doi: 10.3111/13696998.2015.1076826.
13. Efthimiou O, Mavridis D, Cipriani A, Leucht S, Bagos P, Salanti G. An approach for modelling multiple correlated outcomes in a network of interventions using odds ratios. Stat Med. 2014;33(13):2275–87.
14. Efthimiou O, Mavridis D, Riley R, Cipriani A, Salanti G. Joint synthesis of multiple correlated outcomes in networks of interventions. Biostatistics. 2015;16(1):84–97.
15. Goring S, Gustafson P, Liu Y, Saab S, Cline S, Platt R. Disconnected by design: analytic approach in treatment networks having no common comparator. Res Synth Methods. 2016;7(4):420–32.
16. Govan L, Ades A, Weir C, Welton N, Langhorne P. Controlling ecological bias in evidence synthesis of trials reporting on collapsed and overlapping covariate categories. Stat Med. 2010;29(12):1340–56.
17. Hong H, Chu H, Zhang J, Carlin B. A Bayesian missing data framework for generalized multiple outcome mixed treatment comparisons. Res Synth Methods. 2016;7(1):6–22.
18. Hong H, Fu H, Price K, Carlin B. Incorporation of individual-patient data in network meta-analysis for multiple continuous endpoints, with application to diabetes treatment. Stat Med. 2015;34(20):2794–819.
19. Ishak K, Proskorovsky I, Benedict A. Simulation and matching-based approaches for indirect comparison of treatments. Pharmacoeconomics. 2015;33(6):537–49. <http://dx.doi.org/10.1007/s40273-015-0271-1>.
20. Jackson D, Barrett J, Rice S, White I, Higgins J. A design-by-treatment interaction model for network meta-analysis with random inconsistency effects. Stat Med. 2014;33(21):3639–54.
21. Jansen J. Network meta-analysis of survival data with fractional polynomials. BMC Med Res Methodol. 2011;11:61. <http://bmcmedresmethodol.biomedcentral.com/articles/10.1186/1471-2288-11-61>.
22. Jansen J, Vieira M, Cope S. Network meta-analysis of longitudinal data using fractional polynomials. Stat Med. 2015;34(15):2294–311. <http://doi.wiley.com/10.1002/sim.6492>.
23. Kawatkar A, Hay J, Stohl W, Nichol M. Incremental expenditure of biologic disease modifying antirheumatic treatment using instrumental variables in panel data. Health Econ. 2013;22(7):807–23.
24. König J, Krahn U, Binder H. Visualizing the flow of evidence in network meta-analysis and characterizing mixed treatment comparisons. Stat Med. 2013;32(30):5414–29.
25. Li L, Tian J, Tian H, Moher D, Liang F, Jiang T, et al. Network meta-analyses could be improved by searching more sources and by involving a librarian. J Clin Epidemiol. 2014;67(9):1001–7. <http://dx.doi.org/10.1016/j.jclinepi.2014.04.003>.
26. Lu G, Ades A. Modeling between-trial variance structure in mixed treatment comparisons. Biostatistics. 2009;10(4):792–805.
27. Mavridis D, Welton N, Sutton A, Salanti G. A selection model for accounting for publication bias in a full network meta-analysis. Stat Med. 2014;33(30):5399–412.
28. Mavridis D, White I, Higgins J, Cipriani A, Salanti G. Allowing for uncertainty due to missing continuous outcome data in pairwise and network meta-analysis. Stat Med. 2015;34(5):721–41.
29. Menten J, Lesaffre E. A general framework for comparative Bayesian meta-analysis of diagnostic studies. BMC Med Res Methodol. 2015;15(1):70. <http://www.biomedcentral.com/1471-2288/15/70>.
30. Messori A, Fadda V, Maratea D, Trippoli S, Gatto R, De Rosa M, et al. Biological drugs for the treatment of moderate-to-severe psoriasis by subcutaneous route: determining statistical equivalence according to evidence-based methods. Clin Drug Investig. 2014;34(8):593–8.
31. Messori A, Fadda V, Maratea D, Trippoli S, Marinai C. Anti-reabsorptive agents in women with osteoporosis: determining statistical equivalence according to evidence-based methods. J Endocrinol Invest. 2014;37(8):769–73.
32. Mills E, Thorlund K, Ioannidis J. Calculating additive treatment effects from multiple randomized trials provides useful estimates of combination therapies. J Clin Epidemiol. 2012;65(12):1282–8. <http://dx.doi.org/10.1016/j.jclinepi.2012.07.012>.
33. Moreno S, Sutton A, Ades A, Cooper N, Abrams K. Adjusting for publication biases across similar interventions performed well when compared with gold standard data. J Clin Epidemiol. 2011;64(11):1230–41. <http://dx.doi.org/10.1016/j.jclinepi.2011.01.009>.
34. Nikolakopoulou A, Mavridis D, Salanti G. Using conditional power of network meta-analysis (NMA) to inform the design of future clinical trials. Biom J. 2014;56(6):973–90.
35. Noma H, Tanaka S, Matsui S, Cipriani A, Furukawa T. Quantifying indirect evidence in network meta-analysis. Stat Med. 2017;36(6):917–27.
36. Owen R, Tincello D, Keith R. Network meta-analysis: development of a three-level hierarchical modeling approach incorporating dose-related constraints. Value Health. 2015;18(1):116–26.
37. Pallmann P, Pretorius M, Ritz C. Simultaneous comparisons of treatments at multiple time points: Combined marginal models versus joint modeling. Stat Methods Med Res. 2015;pii: 0962280215603743. <http://smm.sagepub.com/cgi/doi/10.1177/0962280215603743>.
38. Pechlivanoglou P, Abegaz F, Postma M, Wit E. An alternative parameterization of Bayesian logistic hierarchical models for mixed treatment comparisons. Pharm Stat. 2015;14(4):322–31.
39. Piepho H. Network-meta analysis made easy: detection of inconsistency using factorial analysis-of-variance models. BMC Med Res Methodol. 2014;14(1):61. <http://bmcmedresmethodol.biomedcentral.com/articles/10.1186/1471-2288-14-61>.
40. Puhan M, Schünemann H, Murad M, Li T, Brignardello-Petersen R, Singh J, et al. A GRADE Working Group approach for rating the quality of treatment effect estimates from network meta-analysis. BMJ. 2014;349:g5630. <http://www.bmj.com/cgi/doi/10.1136/bmj.g5630>.
41. Reken S, Sturtz S, Kiefer C, Böhler Yb, Wieseler B. Assumptions of mixed treatment comparisons in health technology assessments - Challenges and possible steps for practical application. PLoS One. 2016;11(8):e0160712.
42. Ring A, Morris T, Hohl K, Schall R. Indirect bioequivalence assessment using network meta-analyses. Eur J Clin Pharmacol. 2014;70(8):947–55.
43. Salanti G, Del Giovane C, Chaimani A, Caldwell D, Higgins J. Evaluating the quality of evidence from a network meta-analysis. PLoS One. 2014;9(7):e99682.
44. Salanti G, Marinho V, Higgins J. A case study of multiple-treatments meta-analysis demonstrates that covariates should be considered. J Clin Epidemiol. 2009;62(8):857–64. <http://dx.doi.org/10.1016/j.jclinepi.2008.10.001>.
45. Saramago P, Chuang L, Soares M. Network meta-analysis of (individual patient) time to event data alongside (aggregate) count data. BMC Med Res Methodol. 2014;14(1):105. <http://bmcmedresmethodol.biomedcentral.com/articles/10.1186/1471-2288-14-105>.
46. Soini E, Joutseno J, Sumelahti M. Cost-utility of First-line Disease-modifying Treatments for Relapsing–Remitting Multiple Sclerosis. Clin Ther. 2017;39(3):537–557.e10. <http://dx.doi.org/10.1016/j.clinthera.2017.01.028>.
47. Thom H, Capkun G, Cerulli A, Nixon R, Howard L. Network meta-analysis combining individual patient and aggregate data from a mixture of study designs with an application to pulmonary arterial hypertension. BMC Med Res Methodol. 2015;15(1):34.
48. Thorlund K, Druyts E, Toor K, Jansen J, Mills E. Incorporating alternative design clinical trials in network meta-analyses. Clin Epidemiol. 2014;7:29–35.
49. Thorlund K, Thabane L, Mills E. Modelling heterogeneity variances in multiple treatment comparison meta-analysis – are informative priors the better solution? BMC Med Res Methodol. 2013;13(1):2. <https://bmcmedresmethodol.biomedcentral.com/articles/10.1186/1471-2288-13-2>.
50. Tu Y. Use of generalized linear mixed models for network meta-analysis. Med Decis Mak. 2014;34(7):911–8. <http://www.ncbi.nlm.nih.gov/pubmed/25260872>.
51. Tu Y. Using Generalized Linear Mixed Models to Evaluate Inconsistency within a Network Meta-Analysis. Value Health. 2015;18(8):1120–5. <http://linkinghub.elsevier.com/retrieve/pii/S1098301515050706>.
52. Tu Y. Linear mixed model approach to network meta-analysis for continuous outcomes in periodontal research. J Clin Periodontol. 2015;42(2):204–12. <http://doi.wiley.com/10.1111/jcpe.12362>.
53. Vargas C, Espinoza M, Giglio A, Soza A. Cost effectiveness of daclatasvir/asunaprevir versus peginterferon/ribavirin and protease inhibitors for the treatment of hepatitis c genotype 1b Naïve patients in Chile. PLoS One. 2015;10(11):e0141660.
54. Vemer P, Al M, Oppe M, Rutten-Van Mölken M. Mix and match. A simulation study on the impact of mixed-Treatment comparison methods on health-economic outcomes. PLoS One. 2017;12(2):e0171292.
55. Veroniki A, Mavridis D, Higgins J, Salanti G. Characteristics of a loop of evidence that affect detection and estimation of inconsistency: a simulation study. BMC Med Res Methodol. 2014;14:106.
56. Warren F, Abrams K, Sutton A. Hierarchical network meta-analysis models to address sparsity of events and differing treatment classifications with regard to adverse outcomes. Stat Med. 2014;33(14):2449–66.
57. Whegang Youdom S, Samson A, Basco L, Thalabard J. Multiple treatment comparisons in a series of anti-malarial trials with an ordinal primary outcome and repeated treatment evaluations. Malar J. 2012;11:147. <http://www.pubmedcentral.nih.gov/articlerender.fcgi?artid=3496581&tool=pmcentrez&rendertype=abstract>
58. Youn J, Lord J, Hemming K, Girling A, Buxton M. Bayesian meta-analysis on medical devices: application to implantable cardioverter defibrillators. Int J Technol Assess Health Care. 2012;28(2):115–24. <http://www.journals.cambridge.org/abstract_S0266462312000037>.
59. Yu-Kang T. Node-Splitting Generalized Linear Mixed Models for Evaluation of Inconsistency in Network Meta-Analysis. Value Heal. 2016;19(8):957–63.
60. Zhang J, Fu H, Carlin B. Detecting outlying trials in network meta-analysis. Stat Med. 2015;34(19):2695–707.

| **Mixture of RCTs and non-standard RCT design** |
| --- |

1. Ara R, Blake L, Gray L, Hernandez M, Crowther M, Dunkley A, et al. What is the clinical effectiveness and cost-effectiveness of using drugs in treating obese patients in primary care? A systematic review. Health Technol Assess. 2012;16(5):iii–xiv, 1-195.
2. Ara R, Pandor A, Stevens J, Rees A, Rafia R. Early high-dose lipid-lowering therapy to avoid cardiac events: a systematic review and economic evaluation. Health Technol Assess. 2009;13(34):1–74.
3. Bannuru R, Schmid C, Kent D, Vaysbrot E, Wong J, McAlindon T. Comparative effectiveness of pharmacologic interventions for knee osteoarthritis: a systematic review and network meta-analysis. Ann Intern Med. 2015;162(1):46–54.
4. Bodalia P, Grosso A, Sofat R, Macallister R, Smeeth L, Dhillon S, et al. Comparative efficacy and tolerability of anti-epileptic drugs for refractory focal epilepsy: systematic review and network meta-analysis reveals the need for long term comparator trials. Br J Clin Pharmacol. 2013;76(5):649–67.
5. Buti J, Baccini M, Nieri M, La Marca M, Pini-Prato G. Bayesian network meta-analysis of root coverage procedures: ranking efficacy and identification of best treatment. J Clin Periodontol. 2013;40(4):372–86.
6. Caldeira D, Pinto F, Ferreira J. Dyspnea and reversibility profile of P2Y_12_ antagonists: systematic review of new antiplatelet drugs. Am J Cardiovasc Drugs. 2014;14(4):303–11.
7. Calzavara-Pinton P, Zane C, Pacou M, Szeimies R. Bucher’s indirect comparison of daylight photodynamic therapy with methyl aminolevulinate cream versus diclofenac plus hyaluronic acid gel for the treatment of multiple actinic keratosis. Eur J Dermatology. 2016;26(5):487–92.
8. Chen Y, Madan J, Welton N, Yahaya I, Aveyard P, Bauld L, et al. Effectiveness and cost-effectiveness of computer and other electronic aids for smoking cessation: a systematic review and network meta-analysis. Health Technol Assess. 2012;16(38):1–205.
9. Chilton M, Dunkley A, Carter P, Davies M, Khunti K, Gray L. The effect of antiobesity drugs on waist circumference: a mixed treatment comparison. Diabetes Obes Metab. 2014;16(3):237–47.
10. Clive A, Jones H, Bhatnagar R, Preston N, Maskell N. Interventions for the management of malignant pleural effusions: a network meta-analysis. Cochrane Database Syst Rev. 2016;(5):CD01052.
11. da Costa B, Reichenbach S, Keller N, Nartey L, Wandel S, Jüni P, et al. Effectiveness of non-steroidal anti-inflammatory drugs for the treatment of pain in knee and hip osteoarthritis: a network meta-analysis. Lancet. 2016;387(10033):2093–105.
12. Danchin N, Marzilli M, Parkhomenko A, Ribeiro J. Efficacy comparison of trimetazidine with therapeutic alternatives in stable angina pectoris: a network meta-analysis. Cardiology. 2011;120(2):59–72.
13. Datto C, Hellmund R, Siddiqui M. Efficacy and tolerability of naproxen/esomeprazole magnesium tablets compared with non-specific NSAIDs and COX-2 inhibitors: a systematic review and network analyses. Open Access Rheumatoly. 2013;5:1–19.
14. Edwards S, Barton S, Thurgar E, Trevor N. Topotecan, pegylated liposomal doxorubicin hydrochloride, paclitaxel, trabectedin and gemcitabine for advanced recurrent or refractory ovarian cancer: a systematic review and economic evaluation. Health Technol Assess. 2015;19(7):1–480.
15. Faggion C, Listl S, Frühauf N, Chang H, Tu Y. A systematic review and Bayesian network meta-analysis of randomized clinical trials on non-surgical treatments for peri-implantitis. J Clin Periodontol. 2014;41(10):1015–25. <http://doi.wiley.com/10.1111/jcpe.12292>.
16. Foote C, Guyatt G, Vignesh K, Mundi R, Chaudhry H, Heels-Ansdell D, et al. Which Surgical Treatment for Open Tibial Shaft Fractures Results in the Fewest Reoperations? A Network Meta-analysis. Clin Orthop Relat Res. 2015;473(7):2179–92. <http://dx.doi.org/10.1007/s11999-015-4224-y>.
17. Galvan-Banqueri M, Marin Gil R, Santos Ramos B, Bautista Paloma F. Biological treatments for moderate-to-severe psoriasis: indirect comparison. J Clin Pharm Ther. 2013;38(2):121–30.
18. García García M, Rosero Arenas M, Ruiz Granell R, Chorro Gascó F, Martínez Cornejo A. Usefulness of cardiac resynchronisation therapy devices and implantable cardioverter defibrillators in the treatment of heart failure due to severe systolic dysfunction: systematic review of clinical trials and network meta-analysis. Heart Asia. 2016;8(1):8–15. <http://heartasia.bmj.com/lookup/doi/10.1136/heartasia-2015-010634>.
19. Generali D, Venturini S, Rognoni C, Ciani O, Pusztai L, Loi S, et al. A network meta-analysis of everolimus plus exemestane versus chemotherapy in the first- and second-line treatment of estrogen receptor-positive metastatic breast cancer. Breast Cancer Res Treat. 2015;152(1):95–117.
20. Gray L, Cooper N, Dunkley A, Warren F, Ara R, Abrams K, et al. A systematic review and mixed treatment comparison of pharmacological interventions for the treatment of obesity. Obes Rev. 2012;13(6):483–98.
21. Griebeler M, Morey-Vargas O, Brito J, Tsapas A, Wang Z, Carranza Leon B, et al. Pharmacologic interventions for painful diabetic neuropathy: An umbrella systematic review and comparative effectiveness network meta-analysis. Ann Intern Med. 2014;161(9):639–49.
22. Han Y, Stevens A, Dashtipour K, Hauser R, Mari Z. A mixed treatment comparison to compare the efficacy and safety of botulinum toxin treatments for cervical dystonia. J Neurol. 2016;263(4):772–80.
23. Haspinger E, Agustoni F, Torri V, Gelsomino F, Platania M, Zilembo N, et al. Is there evidence for different effects among EGFR-TKIs? Systematic review and meta-analysis of EGFR tyrosine kinase inhibitors (TKIs) versus chemotherapy as first-line treatment for patients harboring EGFR mutations. Crit Rev Oncol Hematol. 2015;94(2):213–27. <http://dx.doi.org/10.1016/j.critrevonc.2014.11.005>.
24. Häuser W, Petzke F, Üceyler N, Sommer C. Comparative efficacy and acceptability of amitriptyline, duloxetine and milnacipran in fibromyalgia syndrome: a systematic review with meta-analysis. Rheumatology. 2011;50(3):532–43.
25. Hornyak M, Scholz H, Kohnen R, Bengel J, Kassubek J, Trenkwalder C. What treatment works best for restless legs syndrome? Meta-analyses of dopaminergic and non-dopaminergic medications. Sleep Med Rev. 2014;18(2):153–64. <http://dx.doi.org/10.1016/j.smrv.2013.03.004>.
26. Imamura M, Abrams P, Bain C, Buckley B, Cardozo L, Cody J, et al. Systematic review and economic modelling of the effectiveness and cost-effectiveness of non-surgical treatments for women with stress urinary incontinence. Health Technol Assess. 2010;14(40):1–188.
27. Katsanos K, Spiliopoulos S, Saha P, Diamantopoulos A, Karunanithy N, Krokidis M, et al. Comparative efficacy and safety of different antiplatelet agents for prevention of major cardiovascular events and leg amputations in patients with peripheral arterial disease: A systematic review and network meta- analysis. PLoS One. 2015;10(8):e013569. <http://dx.doi.org/10.1371/journal.pone.0135692>.
28. Kessler T, Bachmann L, Minder C, Löhrer D, Umbehr M, Schünemann H, et al. Adverse event assessment of antimuscarinics for treating overactive bladder: a network meta-analytic approach. PLoS One. 2011;6(2):e16718.
29. Lapitan M, Cody J. Open retropubic colposuspension for urinary incontinence in women. Cochrane Database Syst Rev. 2016;2:CD002912.
30. Lapitan M, Cody J, Grant A. Open retropubic colposuspension for urinary incontinence in women. Cochrane Database Syst Rev. 2009;(4):CD002912.
31. Le Cleach L, Trinquart L, Do G, Maruani A, Lebrun-Vignes B, Ravaud P, et al. Oral antiviral therapy for prevention of genital herpes outbreaks in immunocompetent and nonpregnant patients. Cochrane Database Syst Rev. 2014;(8):CD009036. <http://doi.wiley.com/10.1002/14651858.CD009036.pub2>.
32. Leibovici-Weissman Y, Neuberger A, Bitterman R, Sinclair D, Salam M, Paul M. Antimicrobial drugs for treating cholera. Cochrane Database Syst Rev. 2014;(6):CD008625. <http://onlinelibrary.wiley.com/doi/10.1002/14651858.CD008625.pub2/abstract>.
33. Lewis R, Williams N, Sutton A, Burton K, Din N, Matar H, et al. Comparative clinical effectiveness of management strategies for sciatica: systematic review and network meta-analyses. Spine J. 2015;15(6):1461–77. <http://dx.doi.org/10.1016/j.spinee.2013.08.049>.
34. Lin L, Zhao Y, Chew P, Sng C, Wong H, Yip L, et al. Comparative efficacy and tolerability of topical prostaglandin analogues for primary open-angle glaucoma and ocular hypertension. Ann Pharmacother. 2014;48(12):1585–93.
35. Lin P, Cheng Y, Chu C, Chien K, Lin C, Tu Y. In-office treatment for dentin hypersensitivity: a systematic review and network meta-analysis. J Clin Periodontol. 2013;40(1):53–64.
36. Liu Y, Yan R, Song A, Niu X, Cao C, Wei J, et al. Aliskiren/amlodipine vs. aliskiren/hydrochlorothiazide in hypertension: indirect meta-analysis of trials comparing the two combinations vs. monotherapy. Am J Hypertens. 2014;27(2):268–78.
37. Maund E, Craig D, Suekarran S, Neilson A, Wright K, Brealey S, et al. Management of frozen shoulder: a systematic review and cost-effectiveness analysis. Health Technol Assess. 2012;16(11):1–264.
38. Maxwell L, Zochling J, Boonen A, Singh J, Veras M, Tanjong Ghogomu E, et al. TNF-alpha inhibitors for ankylosing spondylitis. Cochrane database Syst Rev. 2015;4:CD005468.
39. McIntosh B, Cameron C, Singh S, Yu C, Ahuja T, Welton N, et al. Second-line therapy in patients with type 2 diabetes inadequately controlled with metformin monotherapy: a systematic review and mixed-treatment comparison meta-analysis. Open Med. 2011;5(1):e35-48.
40. Musini V, Nazer M, Bassett K, Wright J. Blood pressure-lowering efficacy of monotherapy with thiazide diuretics for primary hypertension. Cochrane Database Syst Rev. 2014;(5):CD003824.
41. Numthavaj P, Thakkinstian A, Dejthevaporn C, Attia J. Corticosteroid and antiviral therapy for Bell’s palsy: a network meta-analysis. BMC Neurol. 2011;11:1. <http://www.biomedcentral.com/1471-2377/11/1>.
42. Pilkington G, Boland A, Brown T, Oyee J, Bagust A, Dickson R. A systematic review of the clinical effectiveness of first-line chemotherapy for adult patients with locally advanced or metastatic non-small cell lung cancer. Thorax. 2015;70(4):359–67. <http://thorax.bmj.com/cgi/doi/10.1136/thoraxjnl-2014-205914>.
43. Price R, MacLennan G, Glen J. Selective digestive or oropharyngeal decontamination and topical oropharyngeal chlorhexidine for prevention of death in general intensive care: systematic review and network meta-analysis. BMJ. 2014;348:g2197. <http://www.bmj.com/cgi/doi/10.1136/bmj.g2197>.
44. Reinecke H, Weber C, Lange K, Simon M, Stein C, Sorgatz H. Analgesic efficacy of opioids in chronic pain: Recent meta-analyses. Br J Pharmacol. 2015;172(2):324–33.
45. Roskell N, Anzueto A, Hamilton A, Disse B, Becker K. Once-daily long-acting beta-agonists for chronic obstructive pulmonary disease: an indirect comparison of olodaterol and indacatero. Int J Chron Obstruct Pulmon Dis. 2014;9:813–24.
46. Roskell N, Beard S, Zhao Y, Le T. A meta-analysis of pain response in the treatment of fibromyalgia. Pain Pract. 2011;11(6):516–27. <http://onlinelibrary.wiley.com/doi/10.1111/j.1533-2500.2010.00441.x/full>.
47. Rudroju N, Bansal D, Teja Talakokkula S, Gudala K, Hota D, Bhansali A, et al. Comparative efficacy and safety of six antidepressants and anticonvulsants in painful diabetic neuropathy: a network meta-analysis. Pain Physician. 2013;16(6):E705-14.
48. Samarasekera E, Sawyer L, Wonderling D, Tucker R, Smith C. Topical therapies for the treatment of plaque psoriasis: systematic review and network meta-analyses. Br J Dermatol. 2013;168(5):954–67.
49. Schmitt J, Rosumeck S, Thomaschewski G, Sporbeck B, Haufe E, Nast A. Efficacy and safety of systemic treatments for moderate-to-severe psoriasis: meta-analysis of randomized controlled trials. Br J Dermatol. 2014;170(2):274–303.
50. Selph S, Carson S, Fu R, Thakurta S, Low A, McDonagh M. Drug Class Review Neuropathic Pain: Final Update 1 Report. Prepared by the Oregon Evidence-based Practice Center for the Drug Effectiveness Review Project. Oregon Health & Science University. Portland, OR. 2011. Available at: <http://derp.ohsu.edu/about/final-document-display.cfm>.
51. Shams T, Firwana B, Habib F, Alshahrani A, Alnouh B, Murad M, et al. SSRIs for hot flashes: a systematic review and meta-analysis of randomized trials. J Gen Intern Med. 2014;29(1):204–13.
52. Snedecor S, Sudharshan L, Cappelleri J, Sadosky A, Desai P, Jalundhwala Y, et al. Systematic review and meta-analysis of pharmacological therapies for pain associated with postherpetic neuralgia and less common neuropathic conditions. Int J Clin Pract. 2014;68(7):900–18.
53. Snedecor S, Sudharshan L, Cappelleri J, Sadosky A, Mehta S, Botteman M. Systematic review and meta-analysis of pharmacological therapies for painful diabetic peripheral neuropathy. Pain Pract. 2014;14(2):167–84.
54. Stagg H, Harris R, Hatherell H, Obach D, Zhao H, Tsuchiya N, et al. What are the most efficacious treatment regimens for isoniazid-resistant tuberculosis? A systematic review and network meta-analysis. Thorax. 2016;71(10):940–9. <http://thorax.bmj.com/lookup/doi/10.1136/thoraxjnl-2015-208262>.
55. Steiner S, Moertl D, Chen L, Coyle D, Wells G. Network meta-analysis of prasugrel, ticagrelor, high- and standarddose clopidogrel in patients scheduled for percutaneous coronary interventions. Thromb Haemost. 2012;108(2):318–27.
56. Stevens J, Khunti K, Harvey R, Johnson M, Preston L, Woods Hb, et al. Preventing the progression to type 2 diabetes mellitus in adults at high risk: a systematic review and network meta-analysis of lifestyle, pharmacological and surgical interventions. Diabetes Res Clin Pract. 2015;107(3):320–31. <http://dx.doi.org/10.1016/j.diabres.2015.01.027>.
57. Stowe R, Ives N, Clarke C, Deane K, Van H, Wheatley K, et al. Evaluation of the efficacy and safety of adjuvant treatment to levodopa therapy in Parkinson’s disease patients with motor complications. Cochrane Database Syst Rev. 2010;7:CD007166.
58. Sun Y, van Valkenhoef G, Morel T. A mixed treatment comparison of gabapentin enacarbil, pramipexole, ropinirole and rotigotine in moderate-to-severe restless legs syndrome. Curr Med Res Opin. 2014;30(11):2267–78. <http://www.tandfonline.com/doi/full/10.1185/03007995.2014.946124>.
59. Thorlund K, Mills E, Wu P, Ramos E, Chatterjee A, Druyts E, et al. Comparative efficacy of triptans for the abortive treatment of migraine: a multiple treatment comparison meta-analysis. Cephalalgia. 2014;34(4):258–67. <http://journals.sagepub.com/doi/10.1177/0333102413508661>.
60. Thorlund K, Wu P, Druyts E, Eapen S, Mills E. Nonergot dopamine-receptor agonists for treating Parkinson’s disease - a network meta-analysis. Neuropsychiatr Dis Treat. 2014;10:767–76.
61. Tu Y, Needleman I, Chambrone L, Lu H, Faggion CJ. A Bayesian network meta-analysis on comparisons of enamel matrix derivatives, guided tissue regeneration and their combination therapies. J Clin Periodontol. 2012;39(3):303–14. <http://discovery.ucl.ac.uk/1342826/>.
62. Tu Y, Woolston A, Faggion CJ. Do bone grafts or barrier membranes provide additional treatment effects for infrabony lesions treated with enamel matrix derivatives? A network meta-analysis of randomized-controlled trials. J Clin Periodontol. 2010;37(1):59–79.
63. Walsh T, Worthington H, Glenny A, Appelbe P, Marinho V, Shi X. Fluoride toothpastes of different concentrations for preventing dental caries in children and adolescents. Cochrane Database Syst Rev. 2010;(1):CD007868.
64. Wiebe N, Padwal R, Field C, Marks S, Jacobs R, Tonelli M. A systematic review on the effect of sweeteners on glycemic response and clinically relevant outcomes. BMC Med. 2011;9(1):123. <http://bmcmedicine.biomedcentral.com/articles/10.1186/1741-7015-9-123>.
65. Wolff R, Bala M, Westwood M, Kessels A, Kleijnen J. 5% lidocaine medicated plaster in painful diabetic peripheral neuropathy (DPN): a systematic review. Swiss Med Wkly. 2010;140(21–22):297–306.
66. Yuan J, Zhang R, Yang Z, Lee J, Liu Y, Tian J, et al. Comparative effectiveness and safety of oral phosphodiesterase type 5 inhibitors for erectile dysfunction: a systematic review and network meta-analysis. Eur Urol. 2013;63(5):902–12.
67. Zeppetella G, Davies A, Eijgelshoven I, Jansen J. A network meta-analysis of the efficacy of opioid analgesics for the management of breakthrough cancer pain episodes. J Pain Symptom Manage. 2014;47(4):772–785.e5. <http://dx.doi.org/10.1016/j.jpainsymman.2013.05.020>.
68. Zhu G, Huang S, Huang G, Wang L, Lin Y, Wu Y, et al. Optimal drug regimens for primary biliary cirrhosis: a systematic review and network meta-analysis. Oncotarget. 2015;6(27):24533–49.
69. Zhu G, Shi K, Huang S, Huang G, Lin Y, Zhou Z, et al. Network meta-analysis of randomized controlled trials: efficacy and safety of UDCA-based therapies in primary biliary cirrhosis. Medicine (Baltimore). 2015;94(11):e609. <http://content.wkhealth.com/linkback/openurl?sid=WKPTLP:landingpage&an=00005792-201503030-00010>
70. Zong S, Zhao G, Su L, Liang W, Li L, Cheng G, et al. Treatments for the Fifth Metacarpal Neck Fractures: A Network Meta-analysis of Randomized Controlled Trials. Medicine (Baltimore). 2016;95(11):e3059.

| **Mixture of RCTs and observational studies** |
| --- |

1. Bonthapally V, Wu E, Macalalad A, Yang H, Shonukan O, Liu Y, et al. Brentuximab vedotin in relapsed/refractory Hodgkin lymphoma post-autologous transplant: meta-analysis versus historical data. Curr Med Res Opin. 2015;31(5):993–1001.
2. Brazzelli M, Cruickshank M, Tassie E, McNamee P, Robertson C, Elders A, et al. Collagenase clostridium histolyticum for the treatment of Dupuytren’s contracture: systematic review and economic evaluation. Health Technol Assess. 2015;19(90):1–202.
3. Carter P, Achana F, Troughton J, Gray L, Khunti K, Davies M. A Mediterranean diet improves HbA1c but not fasting blood glucose compared to alternative dietary strategies: a network meta-analysis. J Hum Nutr Diet. 2014;27(3):280–97.
4. Cooper N, Kendrick D, Achana F, Dhiman P, He Z, Wynn P, et al. Network meta-analysis to evaluate the effectiveness of interventions to increase the uptake of smoke alarms. Epidemiol Rev. 2012;34:32–45.
5. Donahue K, Jonas D, Hansen R, Roubey R, Jonas B, Lux LJ, et al. Drug Therapy for Rheumatoid Arthritis in Adults: An Update. Comparative Effectiveness Review No. 55. (Prepared by RTI-UNC Evidence-based Practice Center under Contract No. 290-02-0016-I.) Rockville, MD: Agency for Healthcare Research and Quality. April 2012.
6. Gartlehner G, Hansen R, Morgan L, Thaler K, Lux L, Noord M, et al. Comparative benefits and harms of second-generation antidepressants for treating major depressive disorder: an updated meta-analysis. Ann Intern Med. 2011;155(16):772–85.
7. Hutton B, Joseph L, Fergusson D, Mazer C, Shapiro S, Tinmouth A. Risks of harms using antifibrinolytics in cardiac surgery: systematic review and network meta-analysis of randomised and observational studies. BMJ. 2012;345:e5798. <http://www.bmj.com/cgi/doi/10.1136/bmj.e5798>.
8. Koifman E, Lipinski M, Escarcega R, Didier R, Kiramijyan S, Torguson R, et al. Comparison of Watchman device with new oral anti-coagulants in patients with atrial fibrillation: A network meta-analysis. Int J Cardiol. 2016;205:17–22. <http://dx.doi.org/10.1016/j.ijcard.2015.11.181>.
9. Levi Marpillat N, Macquin-Mavier I, Tropeano A, Bachoud-Levi A, Maison P. Antihypertensive classes, cognitive decline and incidence of dementia: a network meta-analysis. J Hypertens. 2013;31(6):1073–82. <http://content.wkhealth.com/linkback/openurl?sid=WKPTLP:landingpage&an=00004872-201306000-00003>
10. Lewis R, Williams N, Matar H, Din N, Fitzsimmons D, Phillips C, et al. The clinical effectiveness and cost-effectiveness of management strategies for sciatica: systematic review and economic model. Health Technol Assess. 2011;15(39):1–578.
11. Loveman E, Copley V, Colquitt J, Scott D, Clegg A, Jones J, et al. The clinical effectiveness and cost-effectiveness of treatments for idiopathic pulmonary fibrosis: a systematic review and economic evaluation. Health Technol Assess. 2015;19(20):i–xxiv.
12. Loveman E, Copley V, Colquitt J, Scott D, Clegg A, Jones J, et al. The effectiveness and cost-effectiveness of treatments for idiopathic pulmonary fibrosis: systematic review, network meta-analysis and health economic evaluation. BMC Pharmacol Toxicol. 2014;15:63.
13. Lu D, Zhang Q, Liu Q, Wang K, Wang S, Shan Q. Bleeding risks with novel oral anticoagulants during catheter ablation of atrial fibrillation: a systematic review and network meta-analysis. J Interv Card Electrophysiol. 2015;44(2):105–11.
14. Manzoli L, de Vito C, Salanti G, D’Addario M, Villari P, Ioannidis J. Meta-analysis of the immunogenicity and tolerability of pandemic influenza A 2009 (H1N1) vaccines. PLoS One. 2011;6(9):e2438.
15. Mumtaz K, Faisal N, Husain S, Morillo A, Renner E, Shah P. Universal prophylaxis or preemptive strategy for cytomegalovirus disease after liver transplantation: a systematic review and meta-analysis. Am J Transplant. 2015;15(2):472–81.
16. Nascimento B, Belfort A, Macedo F, Sant’Anna F, Pereira G, Costa M, et al. Meta-analysis of deferral versus performance of coronary intervention based on coronary pressure-derived fractional flow reserve. Am J Cardiol. 2015;115(3):385–91. <http://dx.doi.org/10.1016/j.amjcard.2014.11.014>.
17. Ramsay C, Adewuyi T, Gray J, Hislop J, Shirley M, Jayakody S, et al. Ablative therapy for people with localised prostate cancer: a systematic review and economic evaluation. Health Technol Assess. 2015;19(49):1–490.
18. Robertson C, Close A, Fraser C, Gurung T, Jia X, Sharma P, et al. Relative effectiveness of robot-assisted and standard laparoscopic prostatectomy as alternatives to open radical prostatectomy for treatment of localised prostate cancer: a systematic review and mixed treatment comparison meta-analysis. BJU Int. 2013;112(6):798–812.
19. Schoenberg M, Marx S, Kersten J, Rösch T, Belle S, Kähler G, et al. Laparoscopic Heller myotomy versus endoscopic balloon dilatation for the treatment of achalasia: a network meta-analysis. Ann Surg. 2013;258(6):943–52. <http://www.ncbi.nlm.nih.gov/pubmed/24220600>.
20. Shamliyan T, Choi J, Ramakrishnan R, Miller J, Wang S, Taylor F, et al. Preventive pharmacologic treatments for episodic migraine in adults. J Gen Intern Med. 2013;28(9):1225–37.
21. Shamliyan T, Kane R, Taylor F. Migraine in Adults: Preventive Pharmacologic Treatments. Comparative Effectiveness Review No. 103. (Prepared by the University of Minnesota Evidence-based Practice Center under Contract No. 290-2007-10064-I) AHRQ Publication No. 13-EHC068-EF. Rockville, MD: Agency for Healthcare Research and Quality; April 2013.
22. Thompson Coon J, Hoyle M, Green C, Liu Z, Welch K, Moxham T, et al. Bevacizumab, sorafenib tosylate, sunitinib and temsirolimus for renal cell carcinoma: a systematic review and economic evaluation. Health Technol Assess. 2010;14(2):1–184.
23. van Walsem A, Pandhi S, Nixon R, Guyot P, Karabis A, Moore R. Relative benefit-risk comparing diclofenac to other traditional non-steroidal anti-inflammatory drugs and cyclooxygenase-2 inhibitors in patients with osteoarthritis or rheumatoid arthritis: a network meta-analysis. Arthritis Res Ther. 2015;17:66. <http://www.pubmedcentral.nih.gov/articlerender.fcgi?artid=4411793&tool=pmcentrez&rendertype=abstract>.
24. Wilhelmus K. Antiviral treatment and other therapeutic interventions for herpes simplex virus epithelial keratitis. Cochrane Database Syst Rev. 2015;(1):CD002898.
25. Zhang X, Wang W, Wang W, Cao N. Effectiveness and safety of controlled venous pressure in liver surgery: a systematic review and network meta-analysis. Biomed Res Int. 2015;2015::290234.
26. Zheng H, Barnett A, Merollini K, Sutton A, Cooper N, Berendt T, et al. Control strategies to prevent total hip replacement-related infections: a systematic review and mixed treatment comparison. BMJ Open. 2014;4(3):e003978. <http://bmjopen.bmj.com/lookup/doi/10.1136/bmjopen-2013-003978>.

| **More interventions than synthesized studies** |
| --- |

1. Albert D. Are All Biologics the Same? Optimal Treatment Strategies for Patients With Early Rheumatoid Arthritis: Systematic Review and Indirect Pairwise Meta-Analysis. J Clin Rheumatol. 2015;21(8):398–404. <http://content.wkhealth.com/linkback/openurl?sid=WKPTLP:landingpage&an=00124743-201512000-00002>.
2. Alotaibi G, Alsaleh K, Wu C, McMurtry M. Dabigatran, rivaroxaban and apixaban for extended venous thromboembolism treatment: network meta-analysis. Int Angiol. 2014;33(4):301–8.
3. Cheng M, Goulart B, Veenstra D, Blough D, Devine E. A network meta-analysis of therapies for previously untreated chronic lymphocytic leukemia. Cancer Treat Rev. 2012;38(8):1004–11. <http://dx.doi.org/10.1016/j.ctrv.2012.02.006>.
4. Cohen A, Hamilton M, Mitchell S, Phatak H, Liu X, Bird A, et al. Comparison of the novel oral anticoagulants apixaban, dabigatran, edoxaban, and rivaroxaban in the initial and long-term treatment and prevention of venous thromboembolism: Systematic review and network meta-analysis. PLoS One. 2015;10(12):e0144856.
5. Derzko C, Sergerie M, Siliman G, Alberton M, Thorlund K. Comparative efficacy and safety of estradiol transdermal preparations for the treatment of vasomotor symptoms in postmenopausal women: an indirect comparison meta-analysis. Menopause. 2016;23(3):294–303.
6. Dranitsaris G, Schmitz S, Broom R. Small molecule targeted therapies for the second-line treatment for metastatic renal cell carcinoma: a systematic review and indirect comparison of safety and efficacy. J Cancer Res Clin Oncol. 2013;139(11):1917–26.
7. Ford J, Jones R, Elders A, Mulatero C, Royle P, Sharma P, et al. Denosumab for treatment of bone metastases secondary to solid tumours: systematic review and network meta-analysis. Eur J Cancer. 2013;49(2):416–30. <http://dx.doi.org/10.1016/j.ejca.2012.07.016>.
8. Ford J, Shyangdan D, Uthman O, Lois N, Waugh N. Drug treatment of macular oedema secondary to central retinal vein occlusion: a network meta-analysis. BMJ Open. 2014;4(7):e005292. <http://bmjopen.bmj.com/cgi/doi/10.1136/bmjopen-2014-005292>.
9. Freeman K, Riley A, Duke D, Fu R. Systematic review and meta-analysis of behavioral interventions for fecal incontinence with constipation. J Pediatr Psychol. 2014;39(8):887–902.
10. Greenhalgh J, Bagust A, Boland A, Martin Saborido C, Oyee J, Blundell M, et al. Clopidogrel and modified-release dipyridamole for the prevention of occlusive vascular events (review of Technology Appraisal No. 90): a systematic review and economic analysis. Health Technol Assess. 2011;15(31):1–178.
11. Huisman E, Papadimitropoulou K, Jarrett J, Bending M, Firth Z, Allen F, et al. Systematic literature review and network meta-analysis in highly active relapsing-remitting multiple sclerosis and rapidly evolving severe multiple sclerosis. BMJ Open. 2017;7(3):e013430. <http://bmjopen.bmj.com/lookup/doi/10.1136/bmjopen-2016-013430>.
12. Katz P, Takyar S, Palmer P, Liedgens H. Sublingual, transdermal and intravenous patient-controlled analgesia for acute post-operative pain: systematic literature review and mixed treatment comparison. Curr Med Res Opin. 2017;33(5):899–910. <https://www.tandfonline.com/doi/full/10.1080/03007995.2017.1294559>
13. Márquez-Cruz M, Díaz-Martínez J, Soto-Molina H, Jorge De Saráchaga A, Cervantes-Arriaga A, Llorens-Arenas R, et al. A systematic review and mixed treatment comparison of monotherapy in early Parkinson’s disease: Implications for Latin America. Expert Rev Pharmacoeconomics Outcomes Res. 2016;16(1):97–102. <http://www.tandfonline.com/doi/abs/10.1586/14737167.2016.1135740?journalCode=ierp20>.
14. McMurray J, Packer M, Desai A, Gong J, Greenlaw N, Lefkowitz M, et al. A putative placebo analysis of the effects of LCZ696 on clinical outcomes in heart failure. Eur Heart J. 2015;36(7):434–9.
15. Mhaskar R, Redzepovic J, Wheatley K, Oac C, Miladinovic B, Glasmacher A, et al. Bisphosphonates in multiple myeloma: a network meta- analysis. Cochrane Database Sytematic Rev. 2012;(5):CD003188.
16. Migliore A, Bizzi E, Bernardi M, Picchianti Diamanti A, Lagana B, Petrella L. Indirect comparison between subcutaneous biologic agents in ankylosing spondylitis. Clin Drug Investig. 2015;35(1):23–9.
17. Migliore A, Broccoli S, Bizzi E, Laganà B. Indirect comparison of the effects of anti-TNF biological agents in patients with ankylosing spondylitis by means of a mixed treatment comparison performed on efficacy data from published randomised, controlled trials. J Med Econ. 2012;15(3):473-80.
18. Morimoto T, Crawford B, Wada K, Ueda S. Comparative efficacy and safety of novel oral anticoagulants in patients with atrial fibrillation: A network meta-analysis with the adjustment for the possible bias from open label studies. J Cardiol. 2015;66(6):466–74. <http://dx.doi.org/10.1016/j.jjcc.2015.05.018>.
19. Nussbaumer B, Morgan L, Reichenpfader U, Greenblatt A, Hansen R, Van Noord M, et al. Comparative efficacy and risk of harms of immediate- versus extended-release second-generation antidepressants: a systematic review with network meta-analysis. CNS Drugs. 2014;28(8):699–712.
20. Police R, Trask P, Wang J, Olivares R, Khan S, Abbe A, et al. Randomized controlled trials in relapsed/refractory chronic lymphocytic leukemia: a systematic review and meta-analysis. Clin Lymphoma, Myeloma Leuk. 2015;15(4):199–207. <http://dx.doi.org/10.1016/j.clml.2014.09.008>.
21. Quigley J, Bryden P, Scott D, Kuwabara H, Cerri K. Relative efficacy and safety of simeprevir and telaprevir in treatment-naïve hepatitis C-infected patients in a Japanese population: A Bayesian network meta-analysis. Hepatol Res. 2015;45(10):E89-98.
22. Simillis C, Li T, Vaughan J, Becker L, Davidson B, Gurusamy K. Methods to decrease blood loss during liver resection: a network meta-analysis. Cochrane Database Syst Rev. 2014;(4):CD010683.
23. Sorensen S, Tam C, Diels J, Sanden S, Wildgust M, Trambitas C, et al. Indirect Comparison of Ibrutinib Versus Idelalisib Plus Ofatumumab in Patients With Previously Treated Chronic Lymphocytic Leukemia. Clin Ther. 2017;39(1):178–189.e5. <http://dx.doi.org/10.1016/j.clinthera.2016.12.001>.
24. van Beurden-Tan C, Franken M, Blommestein H, Uyl-de Groot C, Sonneveld P. Systematic Literature Review and Network Meta-Analysis of Treatment Outcomes in Relapsed and/or Refractory Multiple Myeloma. J Clin Oncol. 2017;35(12):1312–9. <http://ascopubs.org/doi/10.1200/JCO.2016.71.1663>.
25. Verdecchia P, Angeli F, Lip G, Reboldi G. Edoxaban in the evolving scenario of non vitamin K antagonist oral anticoagulants imputed placebo analysis and multiple treatment comparisons. PLoS One. 2014;9(6):e100478.
26. Zagmutt F, Carroll C. Meta-analysis of adverse events in recent randomized clinical trials for dimethyl fumarate, glatiramer acetate and teriflunomide for the treatment of relapsing forms of multiple sclerosis. Int J Neurosci. 2015;125(11):798–807. <http://www.tandfonline.com/doi/full/10.3109/00207454.2014.979982>.
27. Zintzaras E, Doxani C, Mprotsis T, Schmid C, Hadjigeorgiou G. Network analysis of randomized controlled trials in multiple sclerosis. Clin Ther. 2012;34(4):857–869.e9. <http://dx.doi.org/10.1016/j.clinthera.2012.02.018>.

| **Network meta-analysis on laboratory and animal studies** |
| --- |

1. da Silva N, Carriquiry A, O’Neill K, Opriessnig T, O’Connor A. Mixed treatment comparison meta-analysis of porcine circovirus type 2 (PCV2) vaccines used in piglets. Prev Vet Med. 2014;117(3–4):413–24. <http://dx.doi.org/10.1016/j.prevetmed.2014.10.006>.
2. O’Connor A, Yuan C, Cullen J, Coetzee J, da Silva N, Wang C. A mixed treatment meta-analysis of antibiotic treatment options for bovine respiratory disease – An update. Prev Vet Med. 2016;132:130–9. <http://dx.doi.org/10.1016/j.prevetmed.2016.07.003>.
3. Rheinheimer J, Ziegelmann P, Carlessi R, Reck L, Bauer A, Leitão C, et al. Different digestion enzymes used for human pancreatic islet isolation: a mixed treatment comparison (MTC) meta-analysis. Islets. 2014;6(4):e977118.

| **Classical and network meta-analysis on diagnostic studies** |
| --- |

1. Alldred S, Takwoingi Y, Guo B, Pennant M, Deeks J, Neilson J, et al. First trimester serum tests for Down’s syndrome screening. Cochrane Database Syst Rev. 2015;(11):CD011975.
2. Mallee W, Wang J, Poolman R. Computed tomography versus magnetic resonance imaging versus bone scintigraphy for clinically suspected scaphoid fractures in patients with negative plain radiographs. Cochrane Database Syst Rev. 2015;(6):CD010023.
3. Ochodo E, Gopalakrishna G, Spek B, Reitsma J, van Lieshout L, Polman K, et al. Circulating antigen tests and urine reagent strips for diagnosis of active schistosomiasis in endemic areas. Cochrane database Syst Rev. 2015;(3):CD009579.
4. Taggar J, Coleman T, Lewis S, Heneghan C, Jones M. Accuracy of methods for diagnosing atrial fibrillation using 12-lead ECG: A systematic review and meta-analysis. Int J Cardiol. 2015;184:175–83. <http://dx.doi.org/10.1016/j.ijcard.2015.02.014>.
5. Wang J, Bossuyt P, Geskus R, Zwinderman A, Dolleman M, Broer S, et al. Using individual patient data to adjust for indirectness did not successfully remove the bias in this case of comparative test accuracy. J Clin Epidemiol. 2015;68(3):290–8. <http://dx.doi.org/10.1016/j.jclinepi.2014.10.005>.

| **Overview of systematic reviews** |
| --- |

1. Cahill K, Stevens S, Perera R, Lancaster T. Pharmacological interventions for smoking cessation: an overview and network meta-analysis. Cochrane Database Syst Rev. 2013;(5):CD009329.
2. Hartmann-Boyce J, Stead L, Cahill K, Lancaster T. Efficacy of interventions to combat tobacco addiction: Cochrane update of 2013 reviews. Addiction. 2014;109(9):1414–25.
3. Kotb A, Cameron C, Hsieh S, Wells G. Comparative effectiveness of different forms of telemedicine for individuals with heart failure (HF): a systematic review and network meta-analysis. PLoS One. 2015;10(2):e0118681. <http://dx.doi.org/10.1371/journal.pone.0118681>.
4. Moore R, Derry S, Wiffen P, Straube S, Aldington D. Overview review: Comparative efficacy of oral ibuprofen and paracetamol (acetaminophen) across acute and chronic pain conditions. Eur J Pain. 2015;19(9):1213–23.
5. Oh G, Yu J, Choi KS, Joo EJ, Jeong EJ. Simultaneous Comparison of Efficacy and Tolerability of Second-Generation Antipsychotics in Schizophrenia: Mixed-Treatment Comparison Analysis Based on Head-to-Head Trial Data. Psychiatry Investig. 2015;12(1):46–54.
6. Singh J, Cameron D. Summary of AHRQ’s comparative effectiveness review of drug therapy for rheumatoid arthritis (RA) in adults--an update. J Manag Care Pharm. 2012;18(4 Supp C):S1-18.
7. Singh J, Christensen R, Wells G, Suarez-Almazor M, Buchbinder R, Lopez-Olivo M, et al. A network meta-analysis of randomized controlled trials of biologics for rheumatoid arthritis: a Cochrane overview. CMAJ. 2009;181(11):787–96.
8. Singh J, Christensen R, Wells G, Suarez-Almazor M, Buchbinder R, Lopez-Olivo M, et al. Biologics for rheumatoid arthritis: an overview of Cochrane reviews. Cochrane Database Syst Rev. 2009;(4):CD007848. <http://onlinelibrary.wiley.com/doi/10.1002/14651858.CD007848.pub2/abstract>
9. Tricco A, Antony J, Vafaei A, Khan P, Harrington A, Cogo E, et al. Seeking effective interventions to treat complex wounds: an overview of systematic reviews. BMC Med. 2015;13:89. <http://bmcmedicine.biomedcentral.com/articles/10.1186/s12916-015-0288-5>.
10. Wong M, Clarkson J, Glenny A, Lo E, Marinho V, Tsang B, et al. Cochrane reviews on the benefits/risks of fluoride toothpastes. J Dent Res. 2011;90(5):573–9. <http://journals.sagepub.com/doi/10.1177/0022034510393346>.

| **Post-hoc network meta-analysis using published meta-analyses and selected studies** |
| --- |

1. Athanasakis K, Karampli E, Tsounis D, Bilitou A, Kyriopoulos J. Cost-effectiveness of apixaban vs. other new oral anticoagulants for the prevention of stroke: an analysis on patients with non-valvular atrial fibrillation in the Greek healthcare setting. Clin Drug Investig. 2015;35(11):693–705.
2. Cure S, Bianic F, Gavart S, Curtis S, Lee S, Dusheiko G. Cost-effectiveness of telaprevir in combination with pegylated interferon alpha and ribavirin in treatment-experienced chronic hepatitis C genotype 1 patients. J Med Econ. 2014;17(1):77–87. <http://www.tandfonline.com/doi/full/10.3111/13696998.2013.844159>.
3. Cure S, Bianic F, Gavart S, Curtis S, Lee S, Dusheiko G. Cost-effectiveness of telaprevir in combination with pegylated interferon alpha and ribavirin in previously untreated chronic hepatitis C genotype 1 patients. J Med Econ. 2014;17(1):65-76.
4. Diamantopoulos A, Finckh A, Huizinga T, Sungher D, Sawyer L, Neto D, et al. Tocilizumab in the treatment of rheumatoid arthritis: a cost-effectiveness analysis in the UK. Pharmacoeconomics. 2014;32(8):775–87.
5. Gupta T, Kannan S, Ghosh-Laskar S, Agarwal J. Concomitant chemoradiotherapy versus altered fractionation radiotherapy in the radiotherapeutic management of locoregionally advanced head and neck squamous cell carcinoma: An adjusted indirect comparison meta-analysis. Head Neck. 2015;37(5):670–6.
6. Jürgensen J, Ikenberg R, Greiner R, Hösel V. Cost-effectiveness of modern mTOR inhibitor based immunosuppression compared to the standard of care after renal transplantation in Germany. Eur J Heal Econ. 2015;16(4):377–90.
7. Kongnakorn T, Lanitis T, Annemans L, Thijs V, Goethals M, Marbaix S, et al. Stroke and systemic embolism prevention in patients with atrial fibrillation in Belgium: comparative cost effectiveness of new oral anticoagulants and warfarin. Clin Drug Investig. 2015;35(2):109–19.
8. Messori A, Fadda V, Maratea D, Trippoli S, Marinai C. Testing the therapeutic equivalence of novel oral anticoagulants for thromboprophylaxis in orthopedic surgery and for prevention of stroke in atrial fibrillation. Int J Clin Pharmacol Ther. 2015;53(3):211–9.
9. Nazir J, Maman K, Neine M, Briquet B, Odeyemi I, Hakimi Z, et al. Cost-Effectiveness of Mirabegron Compared with Antimuscarinic Agents for the Treatment of Adults with Overactive Bladder in the United Kingdom. Value Health. 2015;18(6):783–90. <http://dx.doi.org/10.1016/j.jval.2015.05.011>.
10. Signorovitch JE, Betts KA, Song Y, Sorg RA, Li J, Behl AS, et al. Comparative efficacy and safety of daclatasvir/asunaprevir versus IFN-based regimens in genotype 1b hepatitis C virus infection. J Comp Eff Res. 2015;4(6):593-605.
11. Tempest M, Sapin C, Beillat M, Robinson P, Treur M. Cost-effectiveness Analysis of Aripiprazole Once-Monthly for the Treatment of Schizophrenia in the UK. J Ment Health Policy Econ. 2015;18(4):185-200.
12. Vellopoulou A, van Agthoven M, van der Kolk A, de Knegt RJ, Berdeaux G, Cure S, et al. Cost utility of telaprevir-PR (peginterferon-ribavirin) versus boceprevir-PR and versus PR alone in chronic hepatitis C in The Netherlands. Appl Health Econ Health Policy. 2014;12(6):647-59.
13. Von Wartburg M, Raymond V, Paradis P. The long-term cost-effectiveness of varenicline (12-week standard course and 12 + 12-week extended course) vs. other smoking cessation strategies in Canada. Int J Clin Pract. 2014;68(5):639–46.
14. Yoo D, Oh C, Hong S, Park W. Analysis of clinical trials of biosimilar infliximab (CT-P13) and comparison against historical clinical studies with the infliximab reference medicinal product. Expert Rev Clin Immunol. 2015;11(Suppl 1):S15-24. <http://www.tandfonline.com/doi/full/10.1586/1744666X.2015.1090314>.
15. Westerhout K, Treur M, Mehnert A, Pascoe K, Ladha I, Belsey J. A cost utility analysis of simeprevir used with peginterferon + ribavirin in the management of genotype 1 hepatitis C virus infection, from the perspective of the UK National Health Service. J Med Econ. 2015;18(10):838-49.

| **Protocol for systematic reviews** |
| --- |

1. Al Khalifah R, Flórez I, Dennis B, Neupane B, Thabane L, Bassilious E. The effectiveness and safety of treatments used for polycystic ovarian syndrome management in adolescents: a systematic review and network meta-analysis protocol. Syst Rev. 2015;4:125. <http://www.pubmedcentral.nih.gov/articlerender.fcgi?artid=4589072&tool=pmcentrez&rendertype=abstract>.
2. Burry L, Hutton B, Guenette M, Williamson D, Mehta S, Egerod I, et al. Comparison of pharmacological and non-pharmacological interventions to prevent delirium in critically ill patients: a protocol for a systematic review incorporating network meta-analyses. Syst Rev. 2016;5(1):153. <http://systematicreviewsjournal.biomedcentral.com/articles/10.1186/s13643-016-0327-0>.
3. Dal Molin A, Faggiano F, Bertoncini F, Buratti G, Busca E, Casarotto R, et al. Bed rest for preventing complications after transfemoral cardiac catheterisation: a protocol of systematic review and network meta-analysis. Syst Rev. 2015;4:47. <http://systematicreviewsjournal.biomedcentral.com/articles/10.1186/s13643-015-0036-0>.
4. DeLozier A, Brown J, Natanegara F, Zhao L, Cui Z, Able S, et al. Study protocol: systematic review and meta-analysis of randomized controlled trials in first-line treatment of squamous non-small cell lung cancer. Syst Rev. 2014;3:102. [http://www.systematicreviewsjournal.com/content/3/1/102%5Cn](http://www.systematicreviewsjournal.com/content/3/1/102\nhttp://ovidsp.ovid.com/ovidweb.cgi?T=JS&PAGE=reference&D=emed12&NEWS=N&AN=2015949940).
5. Dennis B, Bawor M, Paul J, Varenbut M, Daiter J, Plater C, et al. The impact of chronic pain on opioid addiction treatment: a systematic review protocol. Syst Rev. 2015;4:49. <http://www.pubmedcentral.nih.gov/articlerender.fcgi?artid=4403999&tool=pmcentrez&rendertype=abstract>.
6. Dennis B, Naji L, Bawor M, Bonner A, Varenbut M, Daiter J, et al. The effectiveness of opioid substitution treatments for patients with opioid dependence: a systematic review and multiple treatment comparison protocol. Syst Rev. 2014;3:105. <http://systematicreviewsjournal.biomedcentral.com/articles/10.1186/2046-4053-3-105>.
7. Doosti-Irani A, Mansournia M, Rahimi-Foroushani A, Cheraghi Z, Holakouie-Naieni K. Simultaneous Comparison of Efficacy and Adverse Events of Interventions for Patients with Esophageal Cancer: Protocol for a Systematic Review and Bayesian Network Meta-analysis. Asian Pacific J cancer Prev. 2016;17(2):867–72.
8. Furukawa T, Salanti G, Atkinson L, Leucht S, Ruhe H, Turner E, et al. Comparative efficacy and acceptability of first-generation and second-generation antidepressants in the acute treatment of major depression: protocol for a network meta-analysis. BMJ Open. 2016;6(7):e010919. <http://bmjopen.bmj.com/lookup/doi/10.1136/bmjopen-2015-010919>.
9. Gurusamy K, Pissanou T, Pikhart H, Vaughan J, Burroughs A, Davidson B. Methods to decrease blood loss and transfusion requirements for liver transplantation. Cochrane Database Syst Rev. 2011;(12):CD009052. <http://doi.wiley.com/10.1002/14651858.CD009052.pub2>.
10. Jia Y, Leung S. Drug efficacy in treating stable angina pectoris: a protocol for network meta-analysis of randomised controlled trials. BMJ Open. 2014;4(6):e005453.
11. Mulla S, Buckley D, Moulin D, Couban R, Izhar Z, Agarwal A, et al. Management of chronic neuropathic pain: a protocol for a multiple treatment comparison meta-analysis of randomised controlled trials. BMJ Open. 2014;4(11):e006112. <http://bmjopen.bmj.com/lookup/doi/10.1136/bmjopen-2014-006112>.
12. Tricco A, Antony J, Veroniki A, Ashoor H, Hutton B, Hemmelgarn B, et al. Safety and effectiveness of antiretroviral therapies for HIV-infected women and their infants and children: protocol for a systematic review and network meta-analysis. Syst Rev. 2014;3:51. <http://systematicreviewsjournal.biomedcentral.com/articles/10.1186/2046-4053-3-51>.
13. Tricco A, Cogo E, Veroniki A, Soobiah C, Hutton B, Hemmelgarn B, et al. Comparative safety of anti-epileptic drugs among infants and children exposed in utero or during breastfeeding: protocol for a systematic review and network meta-analysis. Syst Rev. 2014;3:68.
14. Zheng H, Chen M, Huang D, Li J, Chen Q, Fang J. Interventions for migraine prophylaxis: protocol of an umbrella systematic review and network meta-analysis. BMJ Open. 2015;5(5):e007594.

| **Published before 2009 (incidence in Zarin et al. (2017))** |
| --- |

1. Goudswaard A, Furlong N, Rutten G, Stolk R, Valk G. Insulin monotherapy versus combinations of insulin with oral hypoglycaemic agents in patients with type 2 diabetes mellitus. Cochrane Database Syst Rev. 2004;(4):CD003418.
2. Macfadyen C, Acuin J, Gamble C. Topical antibiotics without steroids for chronically discharging ears with underlying eardrum perforations. Cochrane Database Syst Rev. 2005;(4):CD004618. <http://doi.wiley.com/10.1002/14651858.CD004618.pub2>.
3. Playford EG, Webster a C, Sorell TC, Craig JC. Antifungal agents for preventing fungal infections in solid organ transplant recipients. Cochrane Database Syst Rev. 2004;(3):CD004291.
4. Pollock A, Baer G, Pomeroy V, Langhorne P. Antifungal agents for preventing fungal infections in solid organ transplant recipients. Cochrane Database Syst Rev. 2007;(3):CD004291.

| **Software report on network meta-analyses** |
| --- |

1. Brown S, Hutton B, Clifford T, Coyle D, Grima D, Wells G, et al. A Microsoft-Excel-based tool for running and critically appraising network meta-analyses—an overview and application of NetMetaXL. Syst Rev. 2014;3(1):110. <http://systematicreviewsjournal.biomedcentral.com/articles/10.1186/2046-4053-3-110>.
2. Chaimani A, Higgins J, Mavridis D, Spyridonos P, Salanti G. raphical tools for network meta-analysis in STATA. PLoS One. 2013;8(10):e76654.
3. Tobías A, Catalá-López F, Roqué M. [Development of an Excel spreadsheet for meta-analysis of indirect and mixed treatment comparisons]. [Article in Spanish]. Rev Esp Salud Publica. 2014;88(1):5–15.

| **Systematic reviews on two interventions** |
| --- |

1. Di Bona D, Plaia A, Leto-Barone M, La Piana S, Di Lorenzo G. Efficacy of Grass Pollen Allergen Sublingual Immunotherapy Tablets for Seasonal Allergic Rhinoconjunctivitis: A Systematic Review and Meta-analysis. JAMA Intern Med. 2015;175(8):1301–9. <http://www.ncbi.nlm.nih.gov/pubmed/26120825>.
2. Nielsen P, Lane D, Rasmussen L, Lip G, Larsen T. Renal function and non-vitamin K oral anticoagulants in comparison with warfarin on safety and efficacy outcomes in atrial fibrillation patients: a systemic review and meta-regression analysis. Clin Res Cardiol. 2015;104(5):418–29. <http://dx.doi.org/10.1007/s00392-014-0797-9>.
3. Wang H, Yuan J, Hu X, Tao K, Liu J, Hu D. The effectiveness and safety of avanafil for erectile dysfunction: a systematic review and meta-analysis. Curr Med Res Opin. 2014;30(8):1565-71. doi: 10.1185/03007995.2014.909391.
4. Wong G, Laugerotte A, Wright J. Blood pressure lowering efficacy of dual alpha and beta blockers for primary hypertension. Cochrane Database Syst Rev. 2015;(8):CD007449. <http://doi.wiley.com/10.1002/14651858.CD007449.pub2>.
5. Zhao H, Li W, Lu Z, Yao Y. Roles of Tregs in development of hepatocellular carcinoma: a meta-analysis. World J Gastroenterol. 2014;20(24):7971–8.

| **Systematic reviews on observational studies** |
| --- |

1. de Bastos M, Stegeman B, Rosendaal F, Van Hylckama Vlieg A, Helmerhorst F, Stijnen T, et al. Combined oral contraceptives: venous thrombosis. Cochrane Database Syst Rev. 2014;(3):CD010813.
2. Mei W, Hu H, Liu Y, Li Z, Wang W. Infliximab is superior to other biological agents for treatment of active ulcerative colitis: A meta-analysis. World J Gastroenterol. 2015;21(19):6044–51. <http://www.pubmedcentral.nih.gov/articlerender.fcgi?artid=4438041&tool=pmcentrez&rendertype=abstract>.
3. Stegeman B, de Bastos M, Rosendaal F, van Hylckama Vlieg, A Helmerhorst F, Stijnen T, Dekkers O. Different combined oral contraceptives and the risk of venous thrombosis: systematic review and network meta-analysis. BMJ. 2013;347:f5298. <http://www.bmj.com/cgi/doi/10.1136/bmj.f5298>.
4. Zhu G, Shi K, You J, Zou H, Lin Y, Wang L, et al. Systematic review with network meta-analysis: adjuvant therapy for resected biliary tract cancer. Aliment Pharmacol Ther. 2014;40(7):759–70.

| **Tutorial on network meta-analysis** |
| --- |

1. Bolaños Díaz R, Calderón Cahua M. [Introduction to traditional meta-analysis]. [Article in Spanish]. Rev Gastroenterol Peru. 2014;34(1):45-51.
2. Bolaños Díaz R, Calderón Cahua M. [Introduction to the indirect meta-analyses]. [Article in Spanish]. Rev Gastroenterol Peru. 2014;34(2):151-4.
3. Catalá-López F, Tobías A, Roqué M. [Basic concepts for network meta-analysis]. [Article in Spanish]. Aten Primaria. 2014;46(10):573–81.
4. Chaiyakunapruk N, Saokaew S, Sruamsiri R, Dilokthornsakul P. Systematic review and network meta-analysis in health technology assessment. J Med Assoc Thai. 2014;97 Suppl 5:S33-42.
5. Cipriani A, Higgins J, Geddes J, Salanti G. Conceptual and technical challenges in network meta-analysis. Ann Intern Med. 2013;159(2):130–7.
6. Foote C, Chaudhry H, Bhandari M, Thabane L, Furukawa T, Petrisor B, et al. Network Meta-analysis: Users’ Guide for Surgeons: Part I - Credibility. Clin Orthop Relat Res. 2015;473(7):2166–71. <http://dx.doi.org/10.1007/s11999-015-4286-x>.
7. García-Perdomo H, Tobías A. [Network meta-analysis: mixed and indirect treatment comparisons. a new method to the service of clinical epidemiology and public health]. [Article in Spanish]. Rev Peru Med Exp Salud Publica. 2016;33(1):149–53.
8. Hartling L, Vandermeer B, Fernandes R. Systematic reviews, overviews of reviews and comparative effectiveness reviews: a discussion of approaches to knowledge synthesis. Evidence-Based Child Heal. 2014;9(2):486–94.
9. Jansen J, Trikalinos T, Cappelleri J, Daw J, Andes S, Eldessouki R, et al. Indirect treatment comparison/network meta-analysis study questionnaire to assess relevance and credibility to inform health care decision making: an ISPOR-AMCP-NPC Good Practice Task Force report. Value Health. 2014;17(2):157–73. <http://dx.doi.org/10.1016/j.jval.2014.01.004>.
10. Kiefer C, Sturtz S, Bender R. Indirect Comparisons and Network Meta-Analyses. Dtsch Ärzteblatt Int. 2015;112(47):803–8. <http://europepmc.org/abstract/med/26634940>.
11. Mills E, Ioannidis J, Thorlund K, Schünemann H, Puhan M, Guyatt G. How to use an article reporting a multiple treatment comparison meta-analysis. JAMA. 2012;308(12):1246–53. <http://jama.jamanetwork.com/article.aspx?doi=10.1001/2012.jama.11228>.
12. Naing C, Reid S, Aung K. Comparing antibiotic treatment for leptospirosis using network meta-analysis: a tutorial. BMC Infect Dis. 2017;17(1):29. <http://bmcinfectdis.biomedcentral.com/articles/10.1186/s12879-016-2145-3>.
13. Ohlssen D, Price K, Xia H, Hong H, Kerman J, Fu H, et al. Guidance on the implementation and reporting of a drug safety Bayesian network meta-analysis. Pharm Stat. 2014;13(1):55–70.
14. Sormani M. Indirect comparisons of treatment effects: Network meta-analyses. Mult Scler J. 2017;23(4):510–2.

| **Valid NMA not feasible (as reported in the publications)** |
| --- |

1. Majumdar A, Roccarina D, Thorburn D, Davidson B, Tsochatzis E, Gurusamy K. Management of people with early- or very early-stage hepatocellular carcinoma: an attempted network meta-analysis. Cochrane Database Syst Rev. 2017;(3):CD011650.
2. Quinn C, Ma Q, Kudlac A, Palmer S, Barber B, Zhao Z. Relative Efficacy of Granulocyte-Macrophage Colony-Stimulating Factor, Dacarbazine, and Glycoprotein 100 in Metastatic Melanoma: An Indirect Treatment Comparison. Adv Ther. 2017;34(2):495–512.
3. Quinn C, Ma Q, Kudlac A, Palmer S, Barber B, Zhao Z. Indirect Treatment Comparison of Talimogene Laherparepvec Compared with Ipilimumab and Vemurafenib for the Treatment of Patients with Metastatic Melanoma. Adv Ther. 2016;33(4):643–57.
