# Supplementary material for: A systematic survey shows that reporting and handling of missing outcome data in networks of interventions is poor
Source: BMC Med Res Methodol. 2018 Oct 24;18:115. doi: 10.1186/s12874-018-0576-9 (PMC6201503; doi:10.1186/s12874-018-0576-9)
Supplement: Supplementary file 2 — Appendix B. List of included articles by year of publication. (DOC 254 kb) [file 12874_2018_576_MOESM2_ESM.doc]

**Appendix B. List of included articles by year of publication**

**2009**

1. Baker W, Baker E, Coleman C. Pharmacologic treatments for chronic obstructive pulmonary disease: a mixed-treatment comparison meta-analysis. Pharmacotherapy. 2009;29(8):891–905. <http://onlinelibrary.wiley.com/o/cochrane/cldare/articles/DARE-12009108053/frame.html>.
2. Bansback N, Sizto S, Sun H, Feldman S, Willian M, Anis A. Efficacy of systemic treatments for moderate to severe plaque psoriasis: systematic review and meta-analysis. Dermatology. 2009;219(3):209–18.
3. Burch J, Paulden M, Conti S, Stock C, Corbett M, Welton N, et al. Antiviral drugs for the treatment of influenza: a systematic review and economic evaluation. Health Technol Assess. 2009;13(58):1–265, iii–iv. <https://www.journalslibrary.nihr.ac.uk/hta/hta13580/>.
4. Cipriani A, Furukawa TA, Salanti G, Geddes JR, Higgins JP, Churchill R, et al. Comparative efficacy and acceptability of 12 new-generation antidepressants: a multiple-treatments meta-analysis. Lancet. 2009;373(9665):746–58. <http://dx.doi.org/10.1016/S0140-6736(09)60046-5>.
5. Edwards SJ, Lind T, Lundell L, Das R. Systematic review: standard- and double-dose proton pump inhibitors for the healing of severe erosive oesophagitis - a mixed treatment comparison of randomized controlled trials. Aliment Pharmacol Ther. 2009;30(6):547–56.
6. Edwards SJ, Clarke MJ, Wordsworth S, Welton NJ. Carbapenems versus other beta-lactams in the treatment of hospitalised patients with infection: a mixed treatment comparison. Curr Med Res Opin. 2009;25(1):251–61.
7. Golfinopoulos V, Pentheroudakis G, Salanti G, Nearchou AD, Ioannidis JPA, Pavlidis N. Comparative survival with diverse chemotherapy regimens for cancer of unknown primary site: multiple-treatments meta-analysis. Cancer Treat Rev. 2009;35(7):570–3. <http://dx.doi.org/10.1016/j.ctrv.2009.05.005>.
8. Hawkins N, Scott DA, Woods BS, Thatcher N. No study left behind: A network meta-analysis in non-small-cell lung cancer demonstrating the importance of considering all relevant data. Value Health. 2009;12(6):996–1003. <http://dx.doi.org/10.1111/j.1524-4733.2009.00541.x>.
9. Hofmeyr GJ, Gülmezoglu AM, Novikova N, Linder V, Ferreira S, Piaggio G. Misoprostol to prevent and treat postpartum haemorrhage: a systematic review and meta-analysis of maternal deaths and dose-related effects. Bull World Health Organ. 2009;87(9):666–77.
10. Jansen JP, Bergman GJD, Huels J, Olson M. Prevention of vertebral fractures in osteoporosis: mixed treatment comparison of bisphosphonate therapies. Curr Med Res Opin. 2009;25(8):1861–8.
11. Kotzé A, Scally A, Howell S. Efficacy and safety of different techniques of paravertebral block for analgesia after thoracotomy: a systematic review and metaregression. Br J Anaesth. 2009;103(5):626–36.
12. Manzoli L, Salanti G, De Vito C, Boccia A, Ioannidis JP, Villari P. Immunogenicity and adverse events of avian influenza A H5N1 vaccine in healthy adults: multiple-treatments meta-analysis. Lancet Infect Dis. 2009;9(8):482–92. <http://dx.doi.org/10.1016/S1473-3099(09)70153-7>.
13. Mills EJ, Perri D, Cooper C, Nachega JB, Wu P, Tleyjeh I, et al. Antifungal treatment for invasive Candida infections: a mixed treatment comparison meta-analysis. Ann Clin Microbiol Antimicrob. 2009;8:23. <http://www.pubmedcentral.nih.gov/articlerender.fcgi?artid=2713200&tool=pmcentrez&rendertype=abstract>.
14. Mills EJ, Rachlis B, O’Regan C, Thabane L, Perri D. Metastatic renal cell cancer treatments: an indirect comparison meta-analysis. BMC Cancer. 2009;9:34.
15. Mills EJ, Wu P, Spurden D, Ebbert JO, Wilson K. Efficacy of pharmacotherapies for short-term smoking abstinance: a systematic review and meta-analysis. Harm Reduct J. 2009;6:25.
16. Puhan MA, Bachmann LM, Kleijnen J, Ter Riet G, Kessels AG. Inhaled drugs to reduce exacerbations in patients with chronic obstructive pulmonary disease: a network meta-analysis. BMC Med. 2009;7:2.
17. Quilici S, Chancellor J, Löthgren M, Simon D, Said G, Le TK, et al. Meta-analysis of duloxetine vs. pregabalin and gabapentin in the treatment of diabetic peripheral neuropathic pain. BMC Neurol. 2009;9:6. <http://bmcneurol.biomedcentral.com/articles/10.1186/1471-2377-9-6>.
18. Strassmann R, Bausch B, Spaar A, Kleijnen J, Braendli O, Puhan MA. Smoking cessation interventions in COPD: A networkmeta-analysis of randomised trials. Eur Respir J. 2009;34(3):634–40.
19. Trikalinos TA, Alsheikh-Ali AA, Tatsioni A, Nallamothu BK, Kent DM. Percutaneous coronary interventions for non-acute coronary artery disease: a quantitative 20-year synopsis and a network meta-analysis. Lancet. 2009;373(9667):911–8. <http://dx.doi.org/10.1016/S0140-6736(09)60319-6>.
20. van der Valk R, Webers CAB, Lumley T, Hendrikse F, Prins MH, Schouten JSAG. A network meta-analysis combined direct and indirect comparisons between glaucoma drugs to rank effectiveness in lowering intraocular pressure. J Clin Epidemiol. 2009;62(12):1279–83. <http://dx.doi.org/10.1016/j.jclinepi.2008.04.012>.
21. Welton NJ, Caldwell DM, Adamopoulos E, Vedhara K. Mixed treatment comparison meta-analysis of complex interventions: psychological interventions in coronary heart disease. Am J Epidemiol. 2009;169(9):1158–65.

**2010**

1. Bergman GJD, Hochberg MC, Boers M, Wintfeld N, Kielhorn A, Jansen JP. Indirect comparison of tocilizumab and other biologic agents in patients with rheumatoid arthritis and inadequate response to disease-modifying antirheumatic drugs. Semin Arthritis Rheum. 2010;39(6):425–41. <http://dx.doi.org/10.1016/j.semarthrit.2009.12.002>.
2. Dakin H, Fidler C, Harper C. Mixed treatment comparison meta-analysis evaluating the relative efficacy of nucleos(t)ides for treatment of nucleos(t)ide-naive patients with chronic hepatitis B. Value Health. 2010;13(8):934–45. <http://dx.doi.org/10.1111/j.1524-4733.2010.00777.x>.
3. Delahoy P, Thompson S, Marschner IC. Pregabalin versus gabapentin in partial epilepsy: a meta-analysis of dose-response relationships. BMC Neurol. 2010;10:104. <http://www.biomedcentral.com/1471-2377/10/104>.
4. Häuser W, Petzke F, Sommer C. Comparative efficacy and harms of duloxetine, milnacipran, and pregabalin in fibromyalgia syndrome. J Pain. 2010;11(6):505–21.
5. McDaid C, Maund E, Rice S, Jenkins B, Woolacott N, Wright K, et al. Paracetamol and selective and non-selective non-steroidal anti-inflammatory drugs (NSAIDs) for the reduction of morphine-related side effects after major surgery: a systematic review. Health Technol Assess. 2010;14(17):1–153, iii–iv.
6. McKenna C, Burch J, Suekarran S, Walker S, Bakhai A, Witte K, et al. A systematic review and economic evaluation of the clinical effectiveness and cost-effectiveness of aldosterone antagonists for postmyocardial infarction heart failure. Health Technol Assess. 2010;14(24):1–162.
7. Middleton LJ, Champaneria R, Daniels JP, Bhattacharya S, Cooper KG, Hilken NH, et al. Hysterectomy, endometrial destruction, and levonorgestrel releasing intrauterine system (Mirena) for heavy menstrual bleeding: systematic review and meta-analysis of data from individual patients. BMJ. 2010;341:c3929. <http://www.bmj.com/content/341/bmj.c3929.long>.
8. Orme M, Collins S, Dakin H, Kelly S, Loftus J. Mixed treatment comparison and meta-regression of the efficacy and safety of prostaglandin analogues and comparators for primary open-angle glaucoma and ocular hypertension. Curr Med Res Opin. 2010;26(3):511–28. <http://www.tandfonline.com/doi/full/10.1185/03007990903498786>.
9. Owen A. Antithrombotic treatment for the primary prevention of stroke in patients with non valvular atrial fibrillation: a reappraisal of the evidence and network meta analysis. Int J Cardiol. 2010;142(3):218–23. <http://dx.doi.org/10.1016/j.ijcard.2009.11.045>.
10. Phung OJ, Scholle JM, Talwar M, Coleman C. Effect of noninsulin antidiabetic drugs added to metformin therapy on glycemic control, weight gain, and hypoglycemia in type 2 diabetes. JAMA. 2010;303(14):1410–8.
11. Piscione F, Piccolo R, Cassese S, Galasso G, De Rosa R, D'Andrea C, et al. Effect of drug-eluting stents in patients with acute ST-segment elevation myocardial infarction undergoing percutaneous coronary intervention: a meta-analysis of randomised trials and an adjusted indirect comparison. EuroIntervention. 2010;5(7):853-60.
12. Riemsma R, Forbes CA, Kessels A, Lykopoulos K, Amonkar MM, Rea DW, et al. Systematic review of aromatase inhibitors in the first-line treatment for hormone sensitive advanced or metastatic breast cancer. Breast Cancer Res Treat. 2010;123(1):9–24.
13. Roskell NS, Lip GYH, Noack H, Clemens A, Plumb JM. Treatments for stroke prevention in atrial fibrillation: a network meta-analysis and indirect comparisons versus dabigatran etexilate. Thromb Haemost. 2010;104(6):1106–15.
14. Uthman OA, Abdulmalik J. Comparative efficacy and acceptability of pharmacotherapeutic agents for anxiety disorders in children and adolescents: a mixed treatment comparison meta-analysis. Curr Med Res Opin. 2010;26(1):53–9.
15. Vissers D, Stam W, Nolte T, Lenre M, Jansen J. Efficacy of intranasal fentanyl spray versus other opioids for breakthrough pain in cancer. Curr Med Res Opin. 2010;26(5):1037–45.
16. Wandel S, Juni P, Tendal B, Nuesch E, Villiger PM, Welton NJ, et al. Effects of glucosamine, chondroitin, or placebo in patients with osteoarthritis of hip or knee: network meta-analysis. BMJ. 2010;341:c4675. <http://www.bmj.com/cgi/doi/10.1136/bmj.c4675>.
17. Wang H, Huang T, Jing J, Jin J, Wang P, Yang M, et al. Effectiveness of different central venous catheters for catheter-related infections: a network meta-analysis. J Hosp Infect. 2010;76(1):1–11. <http://dx.doi.org/10.1016/j.jhin.2010.04.025>.
18. Woo G, Tomlinson G, Nishikawa Y, Kowgier M, Sherman M, Wong DKH, et al. Tenofovir and entecavir are the most effective antiviral agents for chronic hepatitis B: a systematic review and Bayesian meta-analyses. Gastroenterology. 2010;139(4):1218–29. <http://dx.doi.org/10.1053/j.gastro.2010.06.042>.

**2011**

1. Anothaisintawee T, Attia J, Nickel J, Thammakraisorn S, Numthavaj P, McEvoy M, et al. Management of chronic prostatitis/chronic pelvic pain syndrome: a systematic review and network meta-analysis. JAMA. 2011;305(1):78–86.
2. Baldwin D, Woods R, Lawson R, Taylor D. Efficacy of drug treatments for generalised anxiety disorder: systematic review and meta-analysis. BMJ. 2011;342:d1199. <http://www.bmj.com/cgi/doi/10.1136/bmj.d1199>.
3. Bangalore S, Kumar S, Kjeldsen SE, Makani H, Grossman E, Wetterslev J, et al. Antihypertensive drugs and risk of cancer: network meta-analyses and trial sequential analyses of 324,168 participants from randomised trials. Lancet Oncol. 2011;12(1):65–82. <http://dx.doi.org/10.1016/S1470-2045(10)70260-6>.
4. Bekkering GE, Soares-Weiser K, Reid K, Kessels a G, Dahan A, Treede RD, et al. Can morphine still be considered to be the standard for treating chronic pain? A systematic review including pair-wise and network meta-analyses. Curr Med Res Opin. 2011;27(7):1477–91.
5. Bhattacharya S, Middleton LJ, Tsourapas A, Lee AJ, Champaneria R, Daniels JP, et al. Hysterectomy, endometrial ablation and Mirena® for heavy menstrual bleeding: a systematic review of clinical effectiveness and cost-effectiveness analysis. Heal Technol Assess. 2011;15(19):iii–xvi, 1-252.
6. Blanchard P, Hill C, Guihenneuc-Jouyaux C, Baey C, Bourhis J, Pignon JP. Mixed treatment comparison meta-analysis of altered fractionated radiotherapy and chemotherapy in head and neck cancer. J Clin Epidemiol. 2011;64(9):985–92. <http://dx.doi.org/10.1016/j.jclinepi.2010.10.016>.
7. Bottomley JM, Taylor R, Ryttov J. The effectiveness of two-compound formulation calcipotriol and betamethasone dipropionate gel in the treatment of moderately severe scalp psoriasis: a systematic review of direct and indirect evidence. Curr Med Res Opin. 2011;27(1):251–68. <http://www.tandfonline.com/doi/full/10.1185/03007995.2010.541023>.
8. Choy E, Marshall D, Gabriel ZL, Mitchell SA, Gylee E, Dakin HA. A systematic review and mixed treatment comparison of the efficacy of pharmacological treatments for fibromyalgia. Semin Arthritis Rheum. 2011;41(3):335–345.e6. <http://dx.doi.org/10.1016/j.semarthrit.2011.06.003>.
9. Cipriani A, Barbui C, Salanti G, Rendell J, Brown R, Stockton S, et al. Comparative efficacy and acceptability of antimanic drugs in acute mania: a multiple-treatments meta-analysis. Lancet. 2011;378(9799):1306–15. <http://dx.doi.org/10.1016/S0140-6736(11)60873-8>.
10. Cope S, Capkun-Niggli G, Gale R, Jardim JR, Jansen JP. Comparative efficacy of indacaterol 150 μg and 300 μg versus fixed-dose combinations of formoterol + budesonide or salmeterol + fluticasone for the treatment of chronic obstructive pulmonary disease - a network meta-analysis. Int J Chron Obs Pulmon Dis. 2011;6:329–44.
11. Costa J, Fareleira F, Ascena̧ão R, Borges M, Sampaio C, Vaz-Carneiro A. Clinical comparability of the new antiepileptic drugs in refractory partial epilepsy: a systematic review and meta-analysis. Epilepsia. 2011;52(7):1280–91.
12. Devine EB, Alfonso-Cristancho R, Sullivan SD. Effectiveness of biologic therapies for rheumatoid arthritis: an indirect comparisons approach. Pharmacotherapy. 2011;31(1):39–51. <http://doi.wiley.com/10.1592/phco.31.1.39>.
13. Freemantle N, Lafuente-Lafuente C, Mitchell S, Eckert L, Reynolds M. Mixed treatment comparison of dronedarone, amiodarone, sotalol, flecainide, and propafenone, for the management of atrial fibrillation. Europace. 2011;13(3):329–45.
14. Freemantle N, Tharmanathan P, Herbrecht R. Systematic review and mixed treatment comparison of randomized evidence for empirical, pre-emptive and directed treatment strategies for invasive mould disease. J Antimicrob Chemother. 2011;66(Suppl 1):i25-35.
15. Gross JL, Kramer CK, Leitão CB, Hawkins N, Viana L V., Schaan BD, et al. Effect of antihyperglycemic agents added to metformin and a sulfonylurea on glycemic control and weight gain in type 2 diabetes: a network meta-analysis. Ann Intern Med. 2011;154(10):672–9.
16. Guyot P, Taylor P, Christensen R, Pericleous L, Poncet C, Lebmeier M, et al. Abatacept with methotrexate versus other biologic agents in treatment of patients with active rheumatoid arthritis despite methotrexate: a network meta-analysis. Arthritis Res Ther. 2011;13(6):R204. <http://arthritis-research.biomedcentral.com/articles/10.1186/ar3537>.
17. Hartling L, Fernandes RM, Bialy L, Milne A, Johnson D, Plint A, et al. Steroids and bronchodilators for acute bronchiolitis in the first two years of life: systematic review and meta-analysis. BMJ. 2011;342:d1714.
18. Hopkins RB, Goeree R, Pullenayegum E, Adachi JD, Papaioannou A, Xie F, et al. The relative efficacy of nine osteoporosis medications for reducing the rate of fractures in post-menopausal women. BMC Musculoskelet Disord. 2011;12:209. <http://www.biomedcentral.com/1471-2474/12/209>.
19. Ibrahim T, Qureshi A, Sutton AJ, Dias JJ. Surgical versus nonsurgical treatment of acute minimally displaced and undisplaced scaphoid waist fractures: pairwise and network meta-analyses of randomized controlled trials. J Hand Surg Am. 2011;36(11):1759–1768.e1. <http://dx.doi.org/10.1016/j.jhsa.2011.08.033>.
20. Jalota L, Kalira V, George E, Shi Y-Y, Hornuss C, Radke O, et al. Prevention of pain on injection of propofol: systematic review and meta-analysis. BMJ. 2011;342(29):d1110.
21. Jansen JP, Bergman GJD, Huels J, Olson M. The efficacy of bisphosphonates in the prevention of vertebral, hip, and nonvertebral-nonhip fractures in osteoporosis: a network meta-analysis. Semin Arthritis Rheum. 2011;40(4):275-284-2. <http://dx.doi.org/10.1016/j.semarthrit.2010.06.001>.
22. Jones LJ, Craven PD, Attia J, Thakkinstian A, Wright I. Network meta-analysis of indomethacin versus ibuprofen versus placebo for PDA in preterm infants. Arch Dis Child - Fetal Neonatal Ed. 2011;96(1):F45-52. <http://fn.bmj.com/cgi/doi/10.1136/adc.2009.168682>.
23. Klemp M, Tvete IF, Skomedal T, Gaasemyr J, Natvig B, Aursnes I. A review and Bayesian meta-analysis of clinical efficacy and adverse effects of 4 atypical neuroleptic drugs compared with haloperidol and placebo. J Clin Psychopharmacol. 2011;31(6):698–704.
24. Launois R, Avouac B, Berenbaum F, Blin O, Bru I, Fautrel B, et al. Comparison of certolizumab pegol with other anticytokine agents for treatment of rheumatoid arthritis: a multiple-treatment Bayesian metaanalysis. J Rheumatol. 2011;38(5):835–45.
25. Makani H, Bangalore S, Romero J, Wever-Pinzon O, Messerli FH. Effect of renin-angiotensin system blockade on calcium channel blocker-associated peripheral edema. Am J Med. 2011;124(2):128–35. <http://dx.doi.org/10.1016/j.amjmed.2010.08.007>.
26. Maund E, McDaid C, Rice S, Wright K, Jenkins B, Woolacott N. Paracetamol and selective and non-selective non-steroidal anti-inflammatory drugs for the reduction in morphine-related side-effects after major surgery: a systematic review. Br J Anaesth. 2011;106(3):292–7.
27. Mills EJ, Wu P, Chong G, Ghement I, Singh S, Akl EA, et al. Efficacy and safety of statin treatment for cardiovascular disease: a network meta-analysis of 170,255 patients from 76 randomized trials. QJM. 2011;104(2):109–24.
28. Mills EJ, Druyts E, Ghement I, Puhan MA. Pharmacotherapies for chronic obstructive pulmonary disease: a multiple treatment comparison meta-analysis. Clin Epidemiol. 2011;3(1):107–29.
29. Padwal R, Klarenbach S, Wiebe N, Birch D, Karmali S, Manns B, et al. Bariatric surgery: a systematic review and network meta-analysis of randomized trials. Obes Rev. 2011;12(8):602–21.
30. Phung OJ, Sood NA, Sill BE, Coleman CI. Oral anti-diabetic drugs for the prevention of Type 2 diabetes. Diabet Med. 2011;28(8):948–64.
31. Phung OJ, Kahn SR, Cook DJ, Murad MH. Dosing frequency of unfractionated heparin thromboprophylaxis: a meta-analysis. Chest. 2011;140(2):374–81. <http://dx.doi.org/10.1378/chest.10-3084>.
32. Riemsma R, Forbes C, Harker J, Worthy G, Misso K, Schäfer M, et al. Systematic review of tapentadol in chronic severe pain. Curr Med Res Opin. 2011;27(10):1907–30.
33. Rodgers M, Yang H, Craig D, Fonseca T, Myers L, Woolacott N, et al. Etanercept, infliximab and adalimumab for the treatment of psoriatic arthritis: a systematic review and economic evaluation. Health Technol Assess. 2011;15(10):i–xxi, 1-329.
34. Salliot C, Finckh A, Katchamart W, Lu Y, Sun Y, Bombardier C, et al. Indirect comparisons of the efficacy of biological antirheumatic agents in rheumatoid arthritis in patients with an inadequate response to conventional disease-modifying antirheumatic drugs or to an anti-tumour necrosis factor agent: a meta-analysis. Ann Rheum Dis. 2011;70(2):266–71. <http://ard.bmj.com/cgi/doi/10.1136/ard.2010.132134>.
35. Sanches ACC, Correr CJ, Venson R, Pontarolo R. Revisiting the efficacy of long-acting insulin analogues on adults with type 1 diabetes using mixed-treatment comparisons. Diabetes Res Clin Pract. 2011;94(3):333–9.
36. Sciarretta S, Palano F, Tocci G, Baldini R, Volpe M. Antihypertensive treatment and development of heart failure in hypertension: a Bayesian network meta-analysis of studies in patients with hypertension and high cardiovascular risk. Arch Intern Med. 2011;171(5):384–94. <http://www.ncbi.nlm.nih.gov/pubmed/21059964>.
37. Smith B, Peterson K, Fu R, McDonagh M, Thakurta S. Drugs for fibromyalgia. Final original report. Drug Class Reviews. 2011.
38. Squires H, Simpson E, Meng Y, Harnan S, Stevens J, Wong R, et al. A systematic review and economic evaluation of cilostazol, naftidrofuryl oxalate, pentoxifylline and inositol nicotinate for the treatment of intermittent claudication in people with peripheral arterial disease. Health Technol Assess. 2011;15(40):1–210.
39. Stowe R, Ives N, Clarke CE, Handley K, Furmston A, Deane K, et al. Meta-analysis of the comparative efficacy and safety of adjuvant treatment to levodopa in later Parkinson’s disease. Mov Disord. 2011;26(4):587–98.
40. Trelle S, Reichenbach S, Wandel S, Hildebrand P, Tschannen B, Villiger PM, et al. Cardiovascular safety of non-steroidal anti-inflammatory drugs: network meta-analysis. BMJ. 2011;342:c7086. <http://www.bmj.com/cgi/doi/10.1136/bmj.c7086>.
41. Tropeano A, Saleh N, Hawajri N, Macquin-Mavier I, Maison P. Do all antihypertensive drugs improve carotid intima-media thickness? A network meta-analysis of randomized controlled trials. Fundam Clin Pharmacol. 2011;25(3):395–404.
42. Turkstra E, Ng S-K, Scuffham PA. A mixed treatment comparison of the short-term efficacy of biologic disease modifying anti-rheumatic drugs in established rheumatoid arthritis. Curr Med Res Opin. 2011;27(10):1885–97. <http://www.tandfonline.com/doi/full/10.1185/03007995.2011.608655>.
43. van de Kerkhof P, de Peuter R, Ryttov J, Jansen JP. Mixed treatment comparison of a two-compound formulation (TCF) product containing calcipotriol and betamethasone dipropionate with other topical treatments in psoriasis vulgaris. Curr Med Res Opin. 2011;27(1):225–38.
44. Van den Bruel A, Gailly J, Devriese S, Welton NJ, Shortt AJ, Vrijens F. The protective effect of ophthalmic viscoelastic devices on endothelial cell loss during cataract surgery: a meta-analysis using mixed treatment comparisons. Br J Ophthalmol. 2011;95(1):5–10.
45. Vieira MC, Kumar RN, Jansen JP. Comparative effectiveness of efavirenz, protease inhibitors, and raltegravir-based regimens as first-line treatment for HIV-infected adults: a mixed treatment comparison. HIV Clin Trials. 2011;12(4):175–89. <http://www.tandfonline.com/doi/full/10.1310/hct1204-175>.
46. Virgili G, Novielli N, Menchini F, Murro V, Giacomelli G. Pharmacological treatments for neovascular age-related macular degeneration: can mixed treatment comparison meta-analysis be useful? Curr Drug Targets. 2011;12(2):212–20.
47. Wolff RF, Bala MM, Westwood M, Kessels AG, Kleijnen J. 5% lidocaine-medicated plaster vs other relevant interventions and placebo for post-herpetic neuralgia (PHN): a systematic review. Acta Neurol Scand. 2011;123(5):295–309.
48. Ziogas DC, Voulgarelis M, Zintzaras E. A network meta-analysis of randomized controlled trials of induction treatments in acute myeloid leukemia in the elderly. Clin Ther. 2011;33(3):254–79. <http://dx.doi.org/10.1016/j.clinthera.2011.04.004>.

**2012**

1. Alberton M, Wu P, Druyts E, Briel M, Mills EJ. Adverse events associated with individual statin treatments for cardiovascular disease: an indirect comparison meta-analysis. QJM. 2012;105(2):145–57.
2. Asseburg C, Peura P, Oksanen T, Turunen J, Purmonen T, Martikainen J. Cost-effectiveness of oral triptans for acute migraine: mixed treatment comparison. Int J Technol Assess Health Care. 2012;28(4):382–9. <http://www.journals.cambridge.org/abstract_S0266462312000517>.
3. Baker EL, Coleman CI, Reinhart KM, Phung OJ, Kugelman L, Chen W, et al. Effect of Biologic Agents on Non-PASI Outcomes in Moderate-to-Severe Plaque Psoriasis: Systematic Review and Meta-Analyses. Dermatol Ther. 2012;2(1):9.
4. Bally M, Dendukuri N, Sinclair A, Ahern SP, Poisson M, Brophy J. A network meta-analysis of antibiotics for treatment of hospitalised patients with suspected or proven meticillin-resistant Staphylococcus aureus infection. Int J Antimicrob Agents. 2012;40(6):479–95.
5. Bangalore S, Kumar S, Fusaro M, Amoroso N, Attubato MJ, Feit F, et al. Short-and long-term outcomes with drug-eluting and bare-metal coronary stents: a mixed-treatment comparison analysis of 117 762 patient-years of follow-up from randomized trials. Circulation. 2012;125(23):2873–91.
6. Bangalore S, Kumar S, Fusaro M, Amoroso N, Kirtane AJ, Byrne RA, et al. Outcomes with various drug eluting or bare metal stents in patients with diabetes mellitus: mixed treatment comparison analysis of 22,844 patient years of follow-up from randomised trials. BMJ. 2012;345(8):e5170. <http://www.pubmedcentral.nih.gov/articlerender.fcgi?artid=PMC3415955>.
7. Bash LD, Buono JL, Davies GM, Martin A, Fahrbach K, Phatak H, et al. Systematic review and meta-analysis of the efficacy of cardioversion by vernakalant and comparators in patients with atrial fibrillation. Cardiovasc Drugs Ther. 2012;26(2):167–79.
8. Buser N, Ivic S, Kessler TM, Kessels AGH, Bachmann LM. Efficacy and adverse events of antimuscarinics for treating overactive bladder: network meta-analyses. Eur Urol. 2012;62(6):1040–60.
9. Chang KV, Chen SY, Chen WS, Tu YK, Chien KL. Comparative effectiveness of focused shock wave therapy of different intensity levels and radial shock wave therapy for treating plantar fasciitis: a systematic review and network meta-analysis. Arch Phys Med Rehabil. 2012;93(7):1259–68.
10. Cohen A, Drost P, Marchant N, Mitchell S, Orme M, Rublee D, et al. The efficacy and safety of pharmacological prophylaxis of venous thromboembolism following elective knee or hip replacement: systematic review and network meta-analysis. Clin Appl Thromb. 2012;18(6):611–27.
11. Cope S, Zhang J, Williams J, Jansen JP. Efficacy of once-daily indacaterol 75 μg relative to alternative bronchodilators in COPD: a study level and a patient level network meta-analysis. BMC Pulm Med. 2012;12(1):29. <http://bmcpulmmed.biomedcentral.com/articles/10.1186/1471-2466-12-29>.
12. Daniels JP, Middleton LJ, Champaneria R, Khan KS, Cooper K, Mol BWJ, et al. Second generation endometrial ablation techniques for heavy menstrual bleeding: network meta-analysis. BMJ. 2012;344:e2564. <http://www.bmj.com/content/344/bmj.e2564.full>.
13. Del Santo F, Maratea D, Fadda V, Trippoli S, Messori A. Treatments for relapsing-remitting multiple sclerosis: summarising current information by network meta-analysis. Eur J Clin Pharmacol. 2012;68(4):441–8.
14. Dequen P, Lorigan P, Jansen J, van Baardewijk M, Ouwens M, Kotapati S. Systematic Review and Network Meta-Analysis of Overall Survival Comparing 3 mg/kg Ipilimumab With Alternative Therapies in the Management of Pretreated Patients With Unresectable Stage III or IV Melanoma. Oncologist. 2012;17(11):1376–85.
15. Dumville JC, Soares MO, O’Meara S, Cullum N. Systematic review and mixed treatment comparison: dressings to heal diabetic foot ulcers. Diabetologia. 2012;55(7):1902–10.
16. Dunkley AJ, Charles K, Gray LJ, Camosso-Stefinovic J, Davies MJ, Khunti K. Effectiveness of interventions for reducing diabetes and cardiovascular disease risk in people with metabolic syndrome: systematic review and mixed treatment comparison meta-analysis. Diabetes, Obes Metab. 2012;14(7):616–25.
17. Ernest PJG, Viechtbauer W, Schouten JSAG, Beckers HJM, Hendrikse F, Prins MH, et al. The influence of the assessment method on the incidence of visual field progression in glaucoma: a network meta-analysis. Acta Ophthalmol. 2012;90(1):10–9.
18. Fox BD, Kahn SR, Langleben D, Eisenberg MJ, Shimony A. Efficacy and safety of novel oral anticoagulants for treatment of acute venous thromboembolism: direct and adjusted indirect meta-analysis of randomised controlled trials. BMJ. 2012;345:e7498. <http://www.bmj.com/cgi/doi/10.1136/bmj.e7498>.
19. Fretheim A, Odgaard-Jensen J, Brørs O, Madsen S, Njølstad I, Norheim OF, et al. Comparative effectiveness of antihypertensive medication for primary prevention of cardiovascular disease: systematic review and multiple treatments meta-analysis. BMC Med. 2012;10(1):33. <http://bmcmedicine.biomedcentral.com/articles/10.1186/1741-7015-10-33>.
20. Gallego-Galisteo M, Villa-Rubio A, Alegre-Del Rey E, Márquez-Fernández E, Ramos-Báez JJ. Indirect comparison of biological treatments in refractory rheumatoid arthritis. J Clin Pharm Ther. 2012;37(3):301–7.
21. Gómez-Outes A, Terleira-Fernández AI, Suárez-Gea ML, Vargas-Castrillón E. Dabigatran, rivaroxaban, or apixaban versus enoxaparin for thromboprophylaxis after total hip or knee replacement: systematic review, meta-analysis, and indirect treatment comparisons. BMJ. 2012;344:e3675. <http://www.pubmedcentral.nih.gov/articlerender.fcgi?artid=PMC3375207>.
22. Guyot P, Taylor PC, Christensen R, Pericleous L, Drost P, Eijgelshoven I, et al. Indirect treatment comparison of abatacept with methotrexate versus other biologic agents for active rheumatoid arthritis despite methotrexate therapy in the United Kingdom. J Rheumatol. 2012;39(6):1198–206.
23. Haas DM, Caldwell DM, Kirkpatrick P, McIntosh JJ, Welton NJ. Tocolytic therapy for preterm delivery: systematic review and network meta-analysis. BMJ. 2012;345:e6226.
24. Harenberg J, Marx S, Dahl OE, Marder VJ, Schulze A, Wehling M, et al. Interpretation of endpoints in a network meta-analysis of new oral anticoagulants following total hip or total knee replacement surgery. Thromb Haemost. 2012;108(5):903–12.
25. Knottnerus BJ, Grigoryan L, Geerlings SE, Moll van Charante EP, Verheij TJM, Kessels AGH, et al. Comparative effectiveness of antibiotics for uncomplicated urinary tract infections: network meta-analysis of randomized trials. Fam Pract. 2012;29(6):659–70.
26. Lang SH, Manning N, Armstrong N, Misso K, Allen a., Di Nisio M, et al. Treatment with tirofiban for acute coronary syndrome (ACS): a systematic review and network analysis. Curr Med Res Opin. 2012;28(3):351–70.
27. Lin VW, Ringold S, Devine EB. Comparison of Ustekinumab With Other Biological Agents for the Treatment of Moderate to Severe Plaque Psoriasis: A Bayesian Network Meta-analysis. Arch Dermatol. 2012;148(12):1403–10.
28. Littlewood KJ, Higashi K, Jansen JP, Capkun-Niggli G, Balp MM, Doering G, et al. A network meta-analysis of the efficacy of inhaled antibiotics for chronic Pseudomonas infections in cystic fibrosis. J Cyst Fibros. 2012;11(5):419–26. <http://dx.doi.org/10.1016/j.jcf.2012.03.010>.
29. Liu SC, Tu YK, Chien MN, Chien KL. Effect of antidiabetic agents added to metformin on glycaemic control, hypoglycaemia and weight change in patients with type 2 diabetes: a network meta-analysis. Diabetes, Obes Metab. 2012;14(9):810–20.
30. Mak K-H. Coronary and mortality risk of novel oral antithrombotic agents: a meta-analysis of large randomised trials. BMJ Open. 2012;2(5):e001592.
31. Martyn-St James M, Glanville J, McCool R, Duffy S, Cooper J, Hugel P, et al. The efficacy and safety of retigabine and other adjunctive treatments for refractory partial epilepsy: a systematic review and indirect comparison. Seizure. 2012;21(9):665–78. <http://dx.doi.org/10.1016/j.seizure.2012.07.011>.
32. Mills EJ, Wu P, Lockhart I, Thorlund K, Puhan M, Ebbert JO. Comparisons of high-dose and combination nicotine replacement therapy, varenicline, and bupropion for smoking cessation: a systematic review and multiple treatment meta-analysis. Ann Med. 2012;44(6):588–97.
33. Murad MH, Drake MT, Mullan RJ, Mauck KF, Stuart LM, Lane MA, et al. Comparative effectiveness of drug treatments to prevent fragility fractures: a systematic review and network meta-analysis. J Clin Endocrinol Metab. 2012;97(6):1871–80.
34. Palmerini T, Biondi-Zoccai G, Riva D Della, Stettler C, Sangiorgi D, D’Ascenzo F, et al. Stent thrombosis with drug-eluting and bare-metal stents: evidence from a comprehensive network meta-analysis. Lancet. 2012;379(9824):1393–402. <http://dx.doi.org/10.1016/S0140-6736(12)60324-9>.
35. Pichenot M, Deuffic-Burban S, Cuzin L, Yazdanpanah Y. Efficacy of new antiretroviral drugs in treatment-experienced HIV-infected patients: a systematic review and meta-analysis of recent randomized controlled trials. HIV Med. 2012;13(3):148–55.
36. Ramsberg J, Asseburg C, Henriksson M. Effectiveness and cost-effectiveness of antidepressants in primary care: a multiple treatment comparison meta-analysis and cost-effectiveness model. PLoS One. 2012;7(8):e42003.
37. Reich K, Burden AD, Eaton JN, Hawkins NS. Efficacy of biologics in the treatment of moderate to severe psoriasis: a network meta-analysis of randomized controlled trials. Br J Dermatol. 2012;166(1):179–88.
38. Roskell NS, Zimovetz EA, Rycroft CE, Eckert BJ, Tyas DA. Annualized relapse rate of first-line treatments for multiple sclerosis: a meta-analysis, including indirect comparisons versus fingolimod. Curr Med Res Opin. 2012;28(5):767–80.
39. Schmitz S, Adams R, Walsh CD, Barry M, FitzGerald O. A mixed treatment comparison of the efficacy of anti-TNF agents in rheumatoid arthritis for methotrexate non-responders demonstrates differences between treatments: a Bayesian approach. Ann Rheum Dis. 2012;71(2):225–30. <http://ard.bmj.com/lookup/doi/10.1136/annrheumdis-2011-200228>.
40. Stam W, Jansen J, Taylor S. Efficacy of etoricoxib, celecoxib, lumiracoxib, non-selective NSAIDs, and acetaminophen in osteoarthritis: a mixed treatment comparison. Open Rheumatol J. 2012;6(1):6–20. <http://benthamopen.com/ABSTRACT/TORJ-6-6>.
41. Stevens JW, Simpson E, Harnan S, Squires H, Meng Y, Thomas S, et al. Systematic review of the efficacy of cilostazol, naftidrofuryl oxalate and pentoxifylline for the treatment of intermittent claudication. Br J Surg. 2012;99(12):1630–8.
42. Sun F, Yu K, Wu S, Zhang Y, Yang Z, Shi L, et al. Cardiovascular safety and glycemic control of glucagon-like peptide-1 receptor agonists for type 2 diabetes mellitus: a pairwise and network meta-analysis. Diabetes Res Clin Pract. 2012;98(3):386–95.
43. Sun F, Yu K, Yang Z, Wu S, Zhang Y, Shi L, et al. Impact of GLP-1 receptor agonists on major gastrointestinal disorders for type 2 diabetes mellitus: a mixed treatment comparison meta-analysis. Exp Diabetes Res. 2012;2012:230624.
44. Tang DH, Malone DC. A network meta-analysis on the efficacy of serotonin type 3 receptor antagonists used in adults during the first 24 hours for postoperative nausea and vomiting prophylaxis. Clin Ther. 2012;34(2):282–94. <http://dx.doi.org/10.1016/j.clinthera.2012.01.007>.
45. Thakkinstian A, Attia J, Anothaisintawee T, Nickel JC. α-blockers, antibiotics and anti-inflammatories have a role in the management of chronic prostatitis/chronic pelvic pain syndrome. BJU Int. 2012;110(7):1014–22.
46. Thorlund K, Druyts E, Avina-Zubieta JA, Mills EJ. Anti-tumor necrosis factor (TNF) drugs for the treatment of psoriatic arthritis: an indirect comparison meta-analysis. Biologics. 2012;6:417–27.
47. van der Mark LB, Lyklema PE, Geskus RB, Mohrs J, Bindels PJ, van Aalderen WM, et al. A systematic review with attempted network meta-analysis of asthma therapy recommended for five to eighteen year olds in GINA steps three and four. BMC Pulm Med. 2012;12(1):63. <http://bmcpulmmed.biomedcentral.com/articles/10.1186/1471-2466-12-63>.
48. Vejakama P, Thakkinstian A, Lertrattananon D, Ingsathit A, Ngarmukos C, Attia J. Reno-protective effects of renin-angiotensin system blockade in type 2 diabetic patients: a systematic review and network meta-analysis. Diabetologia. 2012;55(3):566–78.
49. Wolff RF, Aune D, Truyers C, Hernandez A V., Misso K, Riemsma R, et al. Systematic review of efficacy and safety of buprenorphine versus fentanyl or morphine in patients with chronic moderate to severe pain. Curr Med Res Opin. 2012;28(5):833–45. <http://www.tandfonline.com/doi/full/10.1185/03007995.2012.678938>.
50. Zagmutt FJ, Tarrants ML. Indirect comparisons of adverse events and dropout rates in early Parkinson’s disease trials of pramipexole, ropinirole, and rasagiline. Int J Neurosci. 2012;122(7):345–53.

**2013**

1. Akshintala VS, Hutfless SM, Colantuoni E, Kim KJ, Khashab MA, Li T, et al. Systematic review with network meta-analysis: pharmacological prophylaxis against post-ERCP pancreatitis. Aliment Pharmacol Ther. 2013;38(11–12):1325–37.
2. Assiri A, Al-Majzoub O, Kanaan AO, Donovan JL, Silva M. Mixed treatment comparison meta-analysis of aspirin, warfarin, and new anticoagulants for stroke prevention in patients with nonvalvular atrial fibrillation. Clin Ther. 2013;35(7):967–984.e2. <http://dx.doi.org/10.1016/j.clinthera.2013.05.011>.
3. Bangalore S, Toklu B, Amoroso N, Fusaro M, Kumar S, Hannan EL, et al. Bare metal stents, durable polymer drug eluting stents, and biodegradable polymer drug eluting stents for coronary artery disease: mixed treatment comparison meta-analysis. BMJ. 2013;347:f6625. <http://www.bmj.com/cgi/doi/10.1136/bmj.f6625>.
4. Bangalore S, Amoroso N, Fusaro M, Kumar S, Feit F. Outcomes with various drug-eluting or bare metal stents in patients with ST-segment-elevation myocardial infarction: a mixed treatment comparison analysis of trial level data from 34 068 patient-years of follow-up from randomized trials. Circ Cardiovasc Interv. 2013;6(4):378–90.
5. Barth J, Munder T, Gerger H, Nüesch E, Trelle S, Znoj H, et al. Comparative efficacy of seven psychotherapeutic interventions for patients with depression: a network meta-analysis. PLoS Med. 2013;10(5):e1001454.
6. Biondi-Zoccai G, Malavasi V, D’Ascenzo F, Abbate A, Agostoni P, Lotrionte M, et al. Comparative effectiveness of novel oral anticoagulants for atrial fibrillation: evidence from pair-wise and warfarin-controlled network meta-analyses. HSR Proc Intensive Care Cardiovasc Anesth. 2013;5:40–54.
7. Braun SR, Gregor B, Tran US. Comparing bona fide psychotherapies of depression in adults with two meta-analytical approaches. PLoS One. 2013;8(6):e68135.
8. Brown T, Pilkington G, Bagust A, Boland A, Oyee J, Tudur-Smith C, et al. Clinical effectiveness and costeffectiveness of first-line chemotherapy for adult patients with locally advanced or metastatic non-small cell lung cancer: a systematic review and economic evaluation. Health Technol Assess. 2013;17(31):1–278.
9. Carroll C, Hummel S, Leaviss J, Ren S, Stevens JW, Everson-Hock E, et al. Clinical effectiveness and cost-effectiveness of minimally invasive techniques to manage varicose veins: a systematic review and economic evaluation. Health Technol Assess. 2013;17(48):i–xvi, 1-141.
10. Castellucci LA, Cameron C, Le Gal G, Rodger MA, Coyle D, Wells PS, et al. Efficacy and safety outcomes of oral anticoagulants and antiplatelet drugs in the secondary prevention of venous thromboembolism: systematic review and network meta-analysis. BMJ. 2013;347:f5133. <http://dx.doi.org/doi:10.1136/bmj.f5133>.
11. Cawston H, Davie A, Paget MA, Skljarevski V, Happich M. Efficacy of duloxetine versus alternative oral therapies: an indirect comparison of randomised clinical trials in chronic low back pain. Eur Spine J. 2013;22(9):1996–2009.
12. Chatterjee S, Biondi-Zoccai G, Abbate A, D’Ascenzo F, Castagno D, Van Tassell B, et al. Benefits of blockers in patients with heart failure and reduced ejection fraction: network meta-analysis. BMJ. 2013;346:f55. <http://www.bmj.com/cgi/doi/10.1136/bmj.f55>.
13. Chatterjee S, Sardar P, Biondi-Zoccai G, Kumbhani DJ. New oral anticoagulants and the risk of intracranial hemorrhage: traditional and Bayesian meta-analysis and mixed treatment comparison of randomized trials of new oral anticoagulants in atrial fibrillation. JAMA Neurol. 2013;70(12):1486–90. <http://archneur.jamanetwork.com/article.aspx?doi=10.1001/jamaneurol.2013.4021>.
14. Cooper C, Lester R, Thorlund K, Druyts E, El Khoury AC, Yaya S, et al. Direct-acting antiviral therapies for hepatitis C genotype 1 infection: a multiple treatment comparison meta-analysis. QJM. 2013;106(2):153–63.
15. Cope S, Donohue JF, Jansen JP, Kraemer M, Capkun-Niggli G, Baldwin M, et al. Comparative efficacy of long-acting bronchodilators for COPD: a network meta-analysis. Respir Res. 2013;14(1):100. [http://dx.doi.org/10.1186/1465-9921-14-100](http://www.ncbi.nlm.nih.gov/pubmed/24093477\nhttp://dx.doi.org/10.1186/1465-9921-14-100).
16. Cope S, Ouwens MJNM, Jansen JP, Schmid P. Progression-free survival with fulvestrant 500 mg and alternative endocrine therapies as second-line treatment for advanced breast cancer: a network meta-analysis with parametric survival models. Value Health. 2013;16(2):403–17. <http://dx.doi.org/10.1016/j.jval.2012.10.019>.
17. Corbett MS, Rice SJC, Madurasinghe V, Slack R, Fayter DA, Harden M, et al. Acupuncture and other physical treatments for the relief of pain due to osteoarthritis of the knee: network meta-analysis. Osteoarthr Cartil. 2013;21(9):1290–8. <http://dx.doi.org/10.1016/j.joca.2013.05.007>.
18. Dong Y-H, Lin H-H, Shau W-Y, Wu Y-C, Chang C-H, Lai M-S. Comparative safety of inhaled medications in patients with chronic obstructive pulmonary disease: systematic review and mixed treatment comparison meta-analysis of randomised controlled trials. Thorax. 2013;68(1):48–56. <http://thorax.bmj.com/lookup/doi/10.1136/thoraxjnl-2012-201926>.
19. Dumville J, McFarlane E, Edwards P, Lipp A, Holmes A, Liu Z. Preoperative skin antiseptic for prevention of surgical wound infections after clean surgery. Cochrane Database Syst Rev. 2013;(3):CD003949.
20. Edwards SJ, Hamilton V, Nherera L, Trevor N. Lithium or an atypical antipsychotic drug in the management of treatment-resistant depression: a systematic review and economic evaluation. Health Technol Assess. 2013;17(54):1–190.
21. Fang Y, Ding Y, Guo Q, Xing J, Long Y, Zong Z. Radioiodine therapy for patients with differentiated thyroid cancer after thyroidectomy: direct comparison and network meta-analyses. J Endocrinol Invest. 2013;36(10):896–902.
22. Filippini G, Del Giovane C, Vacchi L, D’Amico R, Di Pietrantonj C, Beecher D, et al. Immunomodulators and immunosuppressants for multiple sclerosis: a network meta-analysis. Cochrane Database Syst Rev. 2013;(6):CD008933. <http://doi.wiley.com/10.1002/14651858.CD008933.pub2>.
23. Freemantle N, Cooper C, Diez-Perez A, Gitlin M, Radcliffe H, Shepherd S, et al. Results of indirect and mixed treatment comparison of fracture efficacy for osteoporosis treatments: a meta-analysis. Osteoporos Int. 2013;24(1):209–17.
24. Gagne JJ, Bykov K, Choudhry NK, Toomey TJ, Connolly JG, Avorn J. Effect of smoking on comparative efficacy of antiplatelet agents: systematic review, meta-analysis, and indirect comparison. BMJ. 2013;347(7):f5307. <http://www.pubmedcentral.nih.gov/articlerender.fcgi?artid=PMC3775704>.
25. Gao L, Xia L, Zhao FL, Li SC. Clinical efficacy and safety of the newer antiepileptic drugs as adjunctive treatment in adults with refractory partial-onset epilepsy: a meta-analysis of randomized placebo-controlled trials. Epilepsy Res. 2013;103(1):31–44. <http://dx.doi.org/10.1016/j.eplepsyres.2012.06.005>.
26. Goralczyk AD, Cameron S, Amanzada A. Treatment of chronic HCV genotype 1 infection with telaprevir: a Bayesian mixed treatment comparison of fixed-length and response-guided treatment regimens in treatment-naive and -experienced patients. BMC Gastroenterol. 2013;13:148.
27. Gupta AK, Paquet M. Network meta-analysis of the outcome “participant complete clearance” in nonimmunosuppressed participants of eight interventions for actinic keratosis: a follow-up on a Cochrane review. Br J Dermatol. 2013;169(2):250–9.
28. Hadjigeorgiou GM, Doxani C, Miligkos M, Ziakas P, Bakalos G, Papadimitriou D, et al. A network meta-analysis of randomized controlled trials for comparing the effectiveness and safety profile of treatments with marketing authorization for relapsing multiple sclerosis. J Clin Pharm Ther. 2013;38(6):433–9.
29. Hoaglin DC, Filonenko A, Glickman ME, Wasiak R, Gidwani R. Use of mixed-treatment-comparison methods in estimating efficacy of treatments for heavy menstrual bleeding. Eur J Med Res. 2013;18(1):17. <http://eurjmedres.biomedcentral.com/articles/10.1186/2047-783X-18-17>.
30. Howell N, Senanayake E, Freemantle N, Pagano D. Putting the record straight on aprotinin as safe and effective: results from a mixed treatment meta-analysis of trials of aprotinin. J Thorac Cardiovasc Surg. 2013;145(1):234–40. <http://dx.doi.org/10.1016/j.jtcvs.2012.07.018>.
31. Jonas D, Cusack K, Forneris C, Wilkins T, Sonis J, Middleton J, et al. Psychological and Pharmacological Treatments for Adults With Posttraumatic Stress Disorder (PTSD). Psychological and Pharmacological Treatments for Adults With Posttraumatic Stress Disorder (PTSD). Comparative Effectiveness Review No. 92. 2013.
32. Karabis A, Lindner L, Mocarski M, Huisman E, Greening A. Comparative efficacy of aclidinium versus glycopyrronium and tiotropium, as maintenance treatment of moderate to severe COPD patients: a systematic review and network meta-analysis. Int J Chron Obs Pulm Dis. 2013;8:405–23.
33. Khan N, Shah D, Tongbram V, Verdian L, Hawkins N. The efficacy and tolerability of perampanel and other recently approved anti-epileptic drugs for the treatment of refractory partial onset seizure: a systematic review and Bayesian network meta-analysis. Curr Med Res Opin. 2013;29(8):1001–13. <http://www.tandfonline.com/doi/full/10.1185/03007995.2013.803461>.
34. Krogh TP, Bartels EM, Ellingsen T, Stengaard-Pedersen K, Buchbinder R, Fredberg U, et al. Comparative effectiveness of injection therapies in lateral epicondylitis: a systematic review and network meta-analysis of randomized controlled trials. Am J Sports Med. 2013;41(6):1435–46. <http://journals.sagepub.com/doi/10.1177/0363546512458237>.
35. Kwak HJ, Kim JY, Kim YB, Min SK, Moon BK, Kim JY. Pharmacological prevention of rocuronium-induced injection pain or withdrawal movements: a meta-analysis. J Anesth. 2013;27(5):742–9.
36. Kwok CS, Pradhan S, Yeong JKY, Loke YK. Relative effects of two different enoxaparin regimens as comparators against newer oral anticoagulants: meta-analysis and adjusted indirect comparison. Chest. 2013;144(2):593–600. <http://dx.doi.org/10.1378/chest.12-2634>.
37. Landoni G, Greco T, Biondi-Zoccai G, Nigro Neto C, Febres D, Pintaudi M, et al. Anaesthetic drugs and survival: a Bayesian network meta-analysis of randomized trials in cardiac surgery. Br J Anaesth. 2013;111(6):886–96.
38. Leucht S, Cipriani A, Spineli L, Mavridis D, Örey D, Richter F, et al. Comparative efficacy and tolerability of 15 antipsychotic drugs in schizophrenia: a multiple-treatments meta-analysis. Lancet. 2013;382(9896):951–62.
39. Liao WC, Chien KL, Lin YL, Wu MS, Lin JT, Wang HP, et al. Adjuvant treatments for resected pancreatic adenocarcinoma: a systematic review and network meta-analysis. Lancet Oncol. 2013;14(11):1095–103. <http://dx.doi.org/10.1016/S1470-2045(13)70388-7>.
40. Liu J, Dong J, Wang L, Su Y, Yan P, Sun S. Comparative efficacy and acceptability of antidepressants in Parkinson’s disease: a network meta-analysis. PLoS One. 2013;8(10):e76651.
41. Malloy RJ, Kanaan AO, Silva MA, Donovan JL. Evaluation of antiplatelet agents for secondary prevention of stroke using mixed treatment comparison meta-analysis. Clin Ther. 2013;35(10):1490–500. <http://dx.doi.org/10.1016/j.clinthera.2013.09.004>.
42. Mealing S, Barcena L, Hawkins N, Clark J, Eaton V, Hirji I, et al. The relative efficacy of imatinib, dasatinib and nilotinib for newly diagnosed chronic myeloid leukemia: a systematic review and network meta-analysis. Exp Hematol Oncol. 2013;2(1):5. <http://ehoonline.biomedcentral.com/articles/10.1186/2162-3619-2-5>.
43. Meissner K, Fässler M, Rücker G, Kleijnen J, Hróbjartsson A, Schneider A, et al. Differential effectiveness of placebo treatments: a systematic review of migraine prophylaxis. JAMA Intern Med. 2013;173(21):1941–51. <http://archinte.jamanetwork.com/article.aspx?doi=10.1001/jamainternmed.2013.10391>.
44. Migliore A, Broccoli S, Massafra U, Cassol M, Frediani B. Ranking antireabsorptive agents to prevent vertebral fractures in postmenopausal osteoporosis by mixed treatment comparison meta-analysis. Eur Rev Med Pharmacol Sci. 2013;17(5):658–67.
45. Naci H, Brugts JJ, Fleurence R, Ades AE. Comparative effects of statins on major cerebrovascular events: a multiple-treatments meta-analysis of placebo-controlled and active-comparator trials. QJM. 2013;106(4):299–306.
46. Naci H, Brugts JJ, Fleurence R, Ades AE. Dose-comparative effects of different statins on serum lipid levels: a network meta-analysis of 256,827 individuals in 181 randomized controlled trials. Eur J Prev Cardiol. 2013;20(4):658–70.
47. Naci H, Brugts JJ, Fleurence R, Tsoi B, Toor H, Ades AE. Comparative benefits of statins in the primary and secondary prevention of major coronary events and all-cause mortality: a network meta-analysis of placebo-controlled and active-comparator trials. Eur J Prev Cardiol. 2013;20(4):641–57.
48. Navarese EP, Tandjung K, Claessen B, Andreotti F, Kowalewski M, Kandzari DE, et al. Safety and efficacy outcomes of first and second generation durable polymer drug eluting stents and biodegradable polymer biolimus eluting stents in clinical practice: comprehensive network meta-analysis. BMJ]. 2013;347:f6530. <http://www.pubmedcentral.nih.gov/articlerender.fcgi?artid=3819044&tool=pmcentrez&rendertype=abstract>.
49. Navarese EP, Buffon A, Andreotti F, Kozinski M, Welton N, Fabiszak T, et al. Meta-analysis of impact of different types and doses of statins on new-onset diabetes mellitus. Am J Cardiol. 2013;111(8):1123–30. <http://dx.doi.org/10.1016/j.amjcard.2012.12.037>.
50. Nüesch E, Häuser W, Bernardy K, Barth J, Jüni P. Comparative efficacy of pharmacological and non-pharmacological interventions in fibromyalgia syndrome: network meta-analysis. Ann Rheum Dis. 2013;72(6):955–62. <http://ard.bmj.com/lookup/doi/10.1136/annrheumdis-2011-201249>.
51. Palmerini T, Biondi-Zoccai G, Della Riva D, Mariani A, Sabaté M, Valgimigli M, et al. Clinical outcomes with drug-eluting and bare-metal stents in patients with ST-segment elevation myocardial infarction: evidence from a comprehensive network meta-analysis. J Am Coll Cardiol. 2013;62(6):496–504. <http://dx.doi.org/10.1016/j.jacc.2013.05.022>.
52. Palmieri C, Fullarton JR, Brown J. Comparative efficacy of bisphosphonates in metastatic breast and prostate cancer and multiple myeloma: a mixed-treatment meta-analysis. Clin Cancer Res. 2013;19(24):6863–72.
53. Pandor A, Gomersall T, Stevens JW, Wang J, Al-Mohammad A, Bakhai A, et al. Remote monitoring after recent hospital discharge in patients with heart failure: a systematic review and network meta-analysis. Heart. 2013;99(23):1717–26. <http://heart.bmj.com/cgi/doi/10.1136/heartjnl-2013-303811>.
54. Rashiq S, Vandermeer B, Abou-Setta AM, Beaupre LA, Jones CA, Dryden DM. Efficacy of supplemental peripheral nerve blockade for hip fracture surgery: multiple treatment comparison. Can J Anesth. 2013;60(3):230–43.
55. Ribeiro RA, Ziegelmann PK, Duncan BB, Stella SF, da Costa Vieira JL, Restelatto LMF, et al. Impact of statin dose on major cardiovascular events: a mixed treatment comparison meta-analysis involving more than 175,000 patients. Int J Cardiol. 2013;166(2):431–9. <http://dx.doi.org/10.1016/j.ijcard.2011.10.128>.
56. Rotta I, Ziegelmann PK, Otuki MF, Riveros BS, Bernardo NLMC, Correr CJ. Efficacy of topical antifungals in the treatment of dermatophytosis: a mixed-treatment comparison meta-analysis involving 14 treatments. JAMA Dermatology. 2013;149(3):341–9. <http://archderm.jamanetwork.com/article.aspx?doi=10.1001/jamadermatol.2013.1721>.
57. Scott D, Boye K, Timlin L, Clark J, Best J. A network meta-analysis to compare glycaemic control in patients with type 2 diabetes treated with exenatide once weekly or liraglutide once daily in comparison with insulin glargine, exenatide twice daily or placebo. Diabetes Obes Metab. 2013;15(3):213–23.
58. Shi KQ, Liu WY, Pan ZZ, Lin XF, Chen SL, Chen YP, et al. Secondary prophylaxis of variceal bleeding for cirrhotic patients: a multiple-treatments meta-analysis. Eur J Clin Invest. 2013;43(8):844–54.
59. Shu T, Chen GH, Rong L, Feng F, Yang B, Chen R, et al. Indirect comparison of anti-TNF-α agents for active ankylosing spondylitis: mixed treatment comparison of randomized controlled trials. Clin Exp Rheumatol. 2013;31(5):717–22.
60. Skoetz N, Trelle S, Rancea M, Haverkamp H, Diehl V, Engert A, et al. Effect of initial treatment strategy on survival of patients with advanced-stage Hodgkin’s lymphoma: a systematic review and network meta-analysis. Lancet Oncol. 2013;14(10):943–52. <http://dx.doi.org/10.1016/S1470-2045(13)70341-3>.
61. Terasawa T, Trikalinos NA, Djulbegovic B, Trikalinos TA. Comparative efficacy of first-line therapies for advanced-stage chronic lymphocytic leukemia: a multiple-treatment meta-analysis. Cancer Treat Rev. 2013;39(4):340–9. <http://dx.doi.org/10.1016/j.ctrv.2012.05.007>.
62. Uthman OA, van der Windt DA, Jordan JL, Dziedzic KS, Healey EL, Peat GM, et al. Exercise for lower limb osteoarthritis: systematic review incorporating trial sequential analysis and network meta-analysis. BMJ. 2013;347:f5555. <http://www.bmj.com/cgi/doi/10.1136/bmj.f5555>.
63. Wiens A, Lenzi L, Venson R, Correr CJ, Rotta I, Pedroso ML, et al. Comparative efficacy of oral nucleoside or nucleotide analog monotherapy used in chronic hepatitis B: a mixed-treatment comparison meta-analysis. Pharmacotherapy. 2013;33(2):144–51.
64. Wu H-Y, Huang J-W, Lin H-J, Liao W-C, Peng Y-S, Hung K-Y, et al. Comparative effectiveness of renin-angiotensin system blockers and other antihypertensive drugs in patients with diabetes: systematic review and bayesian network meta-analysis. BMJ. 2013;347:f6008. <http://www.bmj.com/cgi/doi/10.1136/bmj.f6008>.
65. Wu MS, Tan SC, Xiong T. Indirect comparison of randomised controlled trials: comparative efficacy of dexlansoprazole vs. esomeprazole in the treatment of gastro-oesophageal reflux disease. Aliment Pharmacol Ther. 2013;38(2):190–201.

**2014**

1. Afghani E, Akshintala V, Khashab M, Law J, Hutfless S, Kim K, et al. 5-Fr vs. 3-Fr pancreatic stents for the prevention of post-ERCP pancreatitis in high-risk patients: a systematic review and network meta-analysis. Endoscopy. 2013;46(7):573–80.
2. Ashby RL, Gabe R, Ali S, Saramago P, Chuang LH, Adderley U, et al. VenUS IV (Venous leg Ulcer Study IV) - compression hosiery compared with compression bandaging in the treatment of venous leg ulcers: a randomised controlled trial, mixed-treatment comparison and decision-analytic model. Health Technol Assess. 2014;18(57):1–293, v–vi.
3. Baji P, Péntek M, Czirják L, Szekanecz Z, Nagy G, Gulácsi L, et al. Efficacy and safety of infliximab-biosimilar compared to other biological drugs in rheumatoid arthritis: a mixed treatment comparison. Eur J Heal Econ. 2014;15(Suppl 1):S53-64.
4. Baji P, Péntek M, Szántó S, Géher P, Gulácsi L, Balogh O, et al. Comparative efficacy and safety of biosimilar infliximab and other biological treatments in ankylosing spondylitis: systematic literature review and meta-analysis. Eur J Heal Econ. 2014;15(Suppl 1):S45-52.
5. Bangalore S, Toklu B, Feit F. Outcomes with coronary artery bypass graft surgery versus percutaneous coronary intervention for patients with diabetes mellitus: can newer generation drug-eluting stents bridge the gap? Circ Cardiovasc Interv. 2014;7(4):518–25.
6. Bangalore S, Toklu B, Kotwal A, Volodarskiy A, Sharma S, Kirtane AJ, et al. Anticoagulant therapy during primary percutaneous coronary intervention for acute myocardial infarction: a meta-analysis of randomized trials in the era of stents and P2Y12 inhibitors. BMJ. 2014;349:g6419. <http://www.bmj.com/content/349/bmj.g6419.long>.
7. Biondi-Zoccai G, Lotrionte M, Thomsen HS, Romagnoli E, D’Ascenzo F, Giordano A, et al. Nephropathy after administration of iso-osmolar and low-osmolar contrast media: Evidence from e network meta-analysis. Int J Cardiol. 2014;172(2):375–80. <http://dx.doi.org/10.1016/j.ijcard.2014.01.075>.
8. Cameron C, Coyle D, Richter T, Kelly S, Gauthier K, Steiner S, et al. Systematic review and network meta-analysis comparing antithrombotic agents for the prevention of stroke and major bleeding in patients with atrial fibrillation. BMJ Open. 2014;4(6):e004301. <http://bmjopen.bmj.com/cgi/doi/10.1136/bmjopen-2013-004301>.
9. Carroll C, Hummel S, Leaviss J, Ren S, Stevens JW, Cantrell A, et al. Systematic review, network meta-analysis and exploratory cost-effectiveness model of randomized trials of minimally invasive techniques versus surgery for varicose veins. Br J Surg. 2014;101(9):1040–52.
10. Caruba T, Katsahian S, Schramm C, Nelson AC, Durieux P, Bégué D, et al. Treatment for stable coronary artery disease: a network meta-analysis of cost-effectiveness studies. PLoS One. 2014;9(6):e98371.
11. Castellucci LA, Cameron C, Le Gal G, Rodger MA, Coyle D, Wells PS, et al. Clinical and safety outcomes associated with treatment of acute venous thromboembolism: a systematic review and meta-analysis. JAMA. 2014;312(11):1122–35. <http://jama.jamanetwork.com/article.aspx?doi=10.1001/jama.2014.10538>.
12. Chan K, Shah K, Lien K, Coyle D, Lam H, Ko YJ. A Bayesian meta-analysis of multiple treatment comparisons of systemic regimens for advanced pancreatic cancer. PLoS One. 2014;9(10):e108749.
13. Chen Y, Zhang Y, Tang Y, Huang X, Xie Y. Long-term clinical efficacy and safety of adding cilostazol to dual antiplatelet therapy for patients undergoing PCI: a meta-analysis of randomized trials with adjusted indirect comparisons. Curr Med Res Opin. 2014;30(1):37–49. <http://www.ncbi.nlm.nih.gov/pubmed/24083626>.
14. Craddy P, Palin H-J, Johnson KI. Comparative effectiveness of dipeptidylpeptidase-4 inhibitors in type 2 diabetes: a systematic review and mixed treatment comparison. Vol. 5, Diabetes Therapy. 2014. 1-41 p. <http://link.springer.com/10.1007/s13300-014-0061-3>.
15. Cucherat M, Stalmans I, Rouland J-F. Relative efficacy and safety of preservative-free latanoprost (T2345) for the treatment of open-angle glaucoma and ocular hypertension: an adjusted Indirect comparison meta-analysis of randomized clinical trials. J Glaucoma. 2014;23(1):e69-75. <http://content.wkhealth.com/linkback/openurl?sid=WKPTLP:landingpage&an=00061198-201401000-00024>.
16. Cui J, Wu B, Liu C, Li Z. A systematic review and adjusted indirect comparison of oral anticoagulants. Orthopedics. 2014;37(11):763–71.
17. Dai N, Xu D, Zhang J, Wei Y, Li W, Fan B, et al. Different β-blockers and initiation time in patients undergoing noncardiac surgery: a meta-analysis. Am J Med Sci. 2014;347(3):235–44.
18. Dai X, Wang H, Jing Z, Fu P. The effect of a dual combination of noninsulin antidiabetic drugs on lipids: a systematic review and network meta-analysis. Curr Med Res Opin. 2014;30(9):1777–86.
19. Dal Molin A, Allara E, Montani D, Milani S, Frassati C, Cossu S, et al. Flushing the central venous catheter: is heparin necessary? J Vasc Access. 2014;15(4):241–8.
20. Danese S, Fiorino G, Peyrin-biroulet L, Lucenteforte E, Virgili G, Moja L, et al. Biological agents for moderately to severely active ulcerative colitis: a systematic review and network meta-analysis. Ann Intern Med. 2014;150(10):704–11.
21. Désaméricq G, Schurhoff F, Meary A, Szöke A, Macquin-Mavier I, Bachoud-Lévi AC, et al. Long-term neurocognitive effects of antipsychotics in schizophrenia: a network meta-analysis. Eur J Clin Pharmacol. 2014;70(2):127–34.
22. Dogliotti A, Paolasso E, Giugliano RP. Current and new oral antithrombotics in non-valvular atrial fibrillation: a network meta-analysis of 79 808 patients. Heart. 2014;100(5):396–405. <http://heart.bmj.com/lookup/doi/10.1136/heartjnl-2013-304347>.
23. Dong J, Gao L, Lu W, Xu Z, Zheng J. Pharmacological interventions for acceleration of the onset time of rocuronium: a meta-analysis. PLoS One. 2014;9(12):e114231.
24. Dooley C, Kaur R, Sobieraj DM. Comparison of the efficacy and safety of low molecular weight heparins for venous thromboembolism prophylaxis in medically ill patients. Curr Med Res Opin. 2014;30(3):367–80. <http://www.tandfonline.com/doi/full/10.1185/03007995.2013.837818>.
25. Dranitsaris G, Ellis AK. Sublingual or subcutaneous immunotherapy for seasonal allergic rhinitis: an indirect analysis of efficacy, safety and cost. J Eval Clin Pract. 2014;20(3):225–38.
26. Ebrahim S, Mollon B, Bance S, Busse JW, Bhandari M. Low-intensity pulsed ultrasonography versus electrical stimulation for fracture healing: a systematic review and network meta-analysis. Can J Surg. 2014;57(3):E105-118.
27. Ellis AG, Reginster J-Y, Luo X, Cappelleri JC, Chines A, Sutradhar S, et al. Bazedoxifene versus oral bisphosphonates for the prevention of nonvertebral fractures in postmenopausal women with osteoporosis at higher risk of fracture: a network meta-analysis. Value Health. 2014;17(4):424–32. <http://linkinghub.elsevier.com/retrieve/pii/S1098301514000163>.
28. Ellis AG, Reginster J-Y, Luo X, G. Bushmakin A, Williams R, Sutradhar S, et al. Indirect comparison of bazedoxifene vs oral bisphosphonates for the prevention of vertebral fractures in postmenopausal osteoporotic women. Curr Med Res Opin. 2014;30(8):1617–26. <http://www.tandfonline.com/doi/full/10.1185/03007995.2014.908279>.
29. Fournier M, Germe M, Theobald K, Scholz GH, Lehmacher W. Indirect comparison of lixisenatide versus neutral protamine Hagedorn insulin as add-on to metformin and sulphonylurea in patients with type 2 diabetes mellitus. Ger Med Sci. 2014;12:Doc14.
30. Furukawa TA, Noma H, Caldwell DM, Honyashiki M, Shinohara K, Imai H, et al. Waiting list may be a nocebo condition in psychotherapy trials: a contribution from network meta-analysis. Acta Psychiatr Scand. 2014;130(3):181–92.
31. Gerger H, Munder T, Gemperli A, Nüesch E, Trelle S, Jüni P, et al. Integrating fragmented evidence by network meta-analysis: relative effectiveness of psychological interventions for adults with post-traumatic stress disorder. Psychol Med. 2014;44(15):3151–64. <http://www.journals.cambridge.org/abstract_S0033291714000853>.
32. González-Vacarezza N, Alemán A, González G, Pérez A. Rituximab and tocilizumab for the treatment of rheumatoid arthritis. Int J Technol Assess Health Care. 2014;30(3):282–8. <http://www.journals.cambridge.org/abstract_S0266462314000221>.
33. Goring S, Hawkins N, Wygant G, Roudaut M, Townsend R, Wood I, et al. Dapagliflozin compared with other oral anti-diabetes treatments when added to metformin monotherapy: a systematic review and network meta-analysis. Diabetes Obes Metab. 2014;16(5):433–42.
34. Graudal N, Hubeck-Graudal T, Tarp S, Christensen R, Jürgens G. Effect of combination therapy on joint destruction in rheumatoid arthritis: a network meta-analysis of randomized controlled trials. PLoS One. 2014;9(9):e10640.
35. Gresham GK, Wells GA, Gill S, Cameron C, Jonker DJ. Chemotherapy regimens for advanced pancreatic cancer: a systematic review and network meta-analysis. BMC Cancer. 2014;14(1):471. <http://bmccancer.biomedcentral.com/articles/10.1186/1471-2407-14-471>.
36. Gupta AK, Daigle D, Lyons DCA. Network meta-analysis of treatments for chronic plaque psoriasis in Canada. J Cutan Med Surg. 2014;18(6):371–8.
37. Gupta AK, Charrette A. The efficacy and safety of 5α-reductase inhibitors in androgenetic alopecia: a network meta-analysis and benefit-risk assessment of finasteride and dutasteride. J Dermatolog Treat. 2014;25(2):156–61.
38. Hutchinson M, Fox RJ, Havrdova E, Kurukulasuriya NC, Sarda SP, Agarwal S, et al. Efficacy and safety of BG-12 (dimethyl fumarate) and other disease-modifying therapies for the treatment of relapsing-remitting multiple sclerosis: a systematic review and mixed treatment comparison. Curr Med Res Opin. 2014;30(4):613–27.
39. Jansen JP, Buckley F, Dejonckheere F, Ogale S. Comparative efficacy of biologics as monotherapy and in combination with methotrexate on patient reported outcomes (PROs) in rheumatoid arthritis patients with an inadequate response to conventional DMARDs – a systematic review and network meta-analysis. Health Qual Life Outcomes. 2014;12(1):102. <http://hqlo.biomedcentral.com/articles/10.1186/1477-7525-12-102>.
40. Johnston BC, Kanters S, Bandayrel K, Wu P, Naji F, Siemieniuk RA, et al. Comparison of weight loss among named diet programs in overweight and obese adults: a meta-analysis. JAMA. 2014;312(9):923–33. <http://jama.jamanetwork.com/article.aspx?doi=10.1001/jama.2014.10397>.
41. Kang N, Sobieraj DM. Indirect treatment comparison of new oral anticoagulants for the treatment of acute venous thromboembolism. Thromb Res. 2014;133(6):1145–51. <http://dx.doi.org/10.1016/j.thromres.2014.03.035>.
42. Katsanos K, Spiliopoulos S, Karunanithy N, Krokidis M, Sabharwal T, Taylor P. Bayesian network meta-analysis of nitinol stents, covered stents, drug-eluting stents, and drug-coated balloons in the femoropopliteal artery. J Vasc Surg. 2014;59(4):1123–1133.e8. <http://dx.doi.org/10.1016/j.jvs.2014.01.041>.
43. Kew K, Dias S, Cates C. Long-acting inhaled therapy (beta-agonists, anticholinergics and steroids) for COPD: a networkmeta-analysis. Cochrane Database Syst Rev. 2014;(3):CD010844.
44. Kriston L, Von Wolff A, Westphal A, Hölzel LP, Härter M. Efficacy and acceptability of acute treatments for persistent depressive disorder: a network meta-analysis. Depress Anxiety. 2014;31(8):621–30
45. Kunitomi T, Hashiguchi M, Mochizuki M. Indirect comparison analysis of efficacy and safety between olanzapine and aripiprazole for schizophrenia. Br J Clin Pharmacol. 2014;77(5):767–76.
46. Laporte S, Chapelle C, Bertoletti L, Lega JC, Cucherat M, Zufferey PJ, et al. Indirect comparison meta-analysis of two enoxaparin regimens in patients undergoing major orthopaedic surgery: impact on the interpretation of thromboprophylactic effects of new anticoagulant drugs. Thromb Haemost. 2014;112(3):503–10.
47. Leaviss J, Sullivan W, Ren S, Everson-Hock E, Stevenson M, Stevens JW, et al. What is the clinical effectiveness and cost-effectiveness of cytisine compared with varenicline for smoking cessation? A systematic review and economic evaluation. Health Technol Assess. 2014;18(33):1–120.
48. Li L, Tian J, Tian H, Sun R, Wang Q, Yang K. The efficacy and safety of different kinds of laparoscopic cholecystectomy: a network meta analysis of 43 randomized controlled trials. PLoS One. 2014;9(2):e90313.
49. Liang W, Wu X, Fang W, Zhao Y, Yang Y, Hu Z, et al. Network meta-analysis of erlotinib, gefitinib, afatinib and icotinib in patients with advanced non-small-cell lung cancer harboring EGFR mutations. PLoS One. 2014;9(2):e85245.
50. Llorca P-M, Lançon C, Brignone M, Rive B, Salah S, Ereshefsky L, et al. Relative efficacy and tolerability of vortioxetine versus selected antidepressants by indirect comparisons of similar clinical studies. Curr Med Res Opin. 2014;30(12):2589–606. <http://www.tandfonline.com/doi/full/10.1185/03007995.2014.969566>.
51. Loke YK, Pradhan S, Yeong JK, Kwok CS. Comparative coronary risks of apixaban, rivaroxaban and dabigatran: a meta-analysis and adjusted indirect comparison. Br J Clin Pharmacol. 2014;78(4):707–17.
52. Loymans RJB, Gemperli A, Cohen J, Rubinstein SM, Sterk PJ, Reddel HK, et al. Comparative effectiveness of long term drug treatment strategies to prevent asthma exacerbations: network meta-analysis. BMJ. 2014;348:g3009. <http://www.bmj.com/cgi/doi/10.1136/bmj.g3009>.
53. Maman K, Aballea S, Nazir J, Desroziers K, Neine ME, Siddiqui E, et al. Comparative efficacy and safety of medical treatments for the management of overactive bladder: a systematic literature review and mixed treatment comparison. Eur Urol. 2014;65(4):755–65. <http://dx.doi.org/10.1016/j.eururo.2013.11.010>.
54. Mealing S, Ghement I, Hawkins N, Scott DA, Lescrauwaet B, Watt M, et al. The importance of baseline viral load when assessing relative efficacy in treatment-naïve HBeAg-positive chronic hepatitis B: a systematic review and network meta-analysis. Syst Rev. 2014;3(1):21. <http://www.pubmedcentral.nih.gov/articlerender.fcgi?artid=4015714&tool=pmcentrez&rendertype=abstract>.
55. Messori A, Fadda V, Maratea D, Trippoli S, Gatto R, De Rosa M, et al. Biological drugs for the treatment of rheumatoid arthritis by the subcutaneous route: interpreting efficacy data to assess statistical equivalence. Ther Adv Musculoskelet Dis. 2014;6(6):207–16. <http://journals.sagepub.com/doi/10.1177/1759720X14554792>.
56. Mills EJ, Thorlund K, Eapen S, Wu P, Prochaska JJ. Cardiovascular events associated with smoking cessation pharmacotherapies: a network meta-analysis. Circulation. 2014;129(1):28–41.
57. Myers J, Wielage RC, Han B, Price K, Gahn J, Paget M-A, et al. The efficacy of duloxetine, non-steroidal anti-inflammatory drugs, and opioids in osteoarthritis: a systematic literature review and meta-analysis. BMC Musculoskelet Disord. 2014;15(1):76. <http://bmcmusculoskeletdisord.biomedcentral.com/articles/10.1186/1471-2474-15-76>.
58. Nagayama A, Hayashida T, Jinno H, Takahashi M, Seki T, Matsumoto A, et al. Comparative effectiveness of neoadjuvant therapy for HER2-positive breast cancer: a network meta-analysis. J Natl Cancer Inst. 2014;106(9):dju203.
59. Oba Y, Lone NA. Comparative efficacy of inhaled corticosteroid and long-acting beta agonist combinations in preventing COPD exacerbations: a Bayesian network meta-analysis. Int J Chron Obs Pulmon Dis. 2014;9:469–79.
60. Oba Y, Lone NA. Mortality benefit of vasopressor and inotropic agents in septic shock: a Bayesian network meta-analysis of randomized controlled trials. J Crit Care. 2014;29(5):706–10. <http://dx.doi.org/10.1016/j.jcrc.2014.04.011>.
61. Orme M, Fenici P, Lomon I, Wygant G, Townsend R, Roudaut M. A systematic review and mixed-treatment comparison of dapagliflozin with existing anti-diabetes treatments for those with type 2 diabetes mellitus inadequately controlled by sulfonylurea monotherapy. Diabetol Metab Syndr. 2014;6:73. <http://dmsjournal.biomedcentral.com/articles/10.1186/1758-5996-6-73>.
62. Palmer S, Saglimbene V, Mavridis D, Salanti G, Craig J, Tonelli M, et al. Erythropoiesis-stimulating agents for anaemia in adults with chronic kidney disease : a network meta-analysis. Cochrane Database Syst Rev. 2014;(12):CD010590.
63. Palmerini T, Biondi-Zoccai G, Della Riva D, Mariani A, Sabaté M, Smits PC, et al. Clinical outcomes with bioabsorbable polymer- Versus durable polymer-based drug-eluting and bare-metal stents: evidence from a comprehensive network meta-analysis. J Am Coll Cardiol. 2014;63(4):299–307. <http://dx.doi.org/10.1016/j.jacc.2013.09.061>.
64. Patel DA, Snedecor SJ, Tang WY, Sudharshan L, Lim JW, Cuffe R, et al. 48-Week efficacy and safety of dolutegravir relative to commonly used third agents in treatment-naive HIV-1-Infected patients: a systematic review and network meta-analysis. PLoS One. 2014;9(9):e105653.
65. Pechlivanoglou P, Le HH, Daenen S, Snowden JA, Postma MJ. Mixed treatment comparison of prophylaxis against invasive fungal infections in neutropenic patients receiving therapy for haematological malignancies: a systematic review. J Antimicrob Chemother. 2014;69(1):1–11.
66. Piccolo R, Galasso G, Piscione F, Esposito G, Trimarco B, Dangas GD, et al. Meta-analysis of randomized trials comparing the effectiveness of different strategies for the treatment of drug-eluting stent restenosis. Am J Cardiol. 2014;114(9):1339–46. <http://linkinghub.elsevier.com/retrieve/pii/S0002914914015902>.
67. Pollock A, Baer G, Campbell P, Choo P, Forster A, Morris J, et al. Physical rehabilitation approaches for recovery of function, balance and walking after stroke. Cochrane Database Syst Rev. 2014;(4):CD001920.
68. Popat S, Mok T, Yang JCH, Wu YL, Lungershausen J, Stammberger U, et al. Afatinib in the treatment of EGFR mutation-positive NSCLC - a network meta-analysis. Lung Cancer. 2014;85(2):230–8. <http://dx.doi.org/10.1016/j.lungcan.2014.05.007>.
69. Régnier S, Malcolm W, Allen F, Wright J, Bezlyak V. Efficacy of anti-VEGF and laser photocoagulation in the treatment of visual impairment due to diabetic macular edema: a systematic review and network meta-analysis. PLoS One. 2014;9(7):e102309.
70. Rochwerg B, Alhazzani W, Sindi A, Heels-Ansdell D, Thabane L, Fox-Robichaud A, et al. Fluid resuscitation in sepsis: a systematic review and network meta-analysis. Ann Intern Med. 2014;161(5):347–55.
71. Rollins BM, Silva MA, Donovan JL, Kanaan AO. Evaluation of oral anticoagulants for the extended treatment of venous thromboembolism using a mixed-treatment comparison, meta-analytic approach. Clin Ther. 2014;36(10):1454–64.e3. <http://dx.doi.org/10.1016/j.clinthera.2014.06.033>.
72. Roskell NS, Setyawan J, Zimovetz E, Hodgkins P. Systematic evidence synthesis of treatments for ADHD in children and adolescents: indirect treatment comparisons of lisdexamfetamine with methylphenidate and atomoxetine. Curr Med Res Opin. 2014;30(8):1673–85.
73. Schwingshackl L, Missbach B, Dias S, König J, Hoffmann G. Impact of different training modalities on glycaemic control and blood lipids in patients with type 2 diabetes: a systematic review and network meta-analysis. Diabetologia. 2014;57(9):1789–97.
74. Singh S, Garg S, Pardi D, Wang Z, Murad M, Loftus EJ. Comparative efficacy of biologic therapy in biologic-naïve patients with Crohn disease: a systematic review and network meta-analysis. Mayo Clin Protoc. 2014;89(12):1621–35.
75. Stagg HR, Zenner D, Harris RJ, Muñoz L, Lipman MC, Abubakar I. Treatment of latent tuberculosis infection a network meta-analysis. Ann Intern Med. 2014;161(6):419–28.
76. Stidham RW, Lee TCH, Higgins PDR, Deshpande AR, Sussman DA, Singal AG, et al. Systematic review with network meta-analysis: the efficacy of anti-TNF agents for the treatment of Crohn’s disease. Aliment Pharmacol Ther. 2014;39(12):1349–62. <http://doi.wiley.com/10.1111/apt.12749>.
77. Stidham RW, Lee TCH, Higgins PDR, Deshpande AR, Sussman DA, Singal AG, et al. Systematic review with network meta-analysis: the efficacy of anti-tumour necrosis factor-alpha agents for the treatment of ulcerative colitis. Aliment Pharmacol Ther. 2014;39(7):660–71. <http://doi.wiley.com/10.1111/apt.12749>.
78. Tian SY, Feldman BM, Beyene J, Brown PE, Uleryk EM, Silverman ED. Immunosuppressive therapies for the induction treatment of proliferative lupus nephritis: a systematic review and network metaanalysis. J Rheumatol. 2014;41(10):1998–2007.
79. Vegter S, Tolley K. A network meta-analysis of the relative efficacy of treatments for actinic keratosis of the face or scalp in Europe: response to comments from Prof Dirschka. PLoS One. 2014;9(6):e96829.
80. Wang H, Yuan J, Hu X, Tao K, Liu J, Hu D. The effectiveness and safety of avanafil for erectile dysfunction: a systematic review and meta-analysis. Curr Med Res Opin. 2014;30(8):1565–71. <http://www.tandfonline.com/doi/full/10.1185/03007995.2014.909391>.
81. Wang J-C, Tian J-H, Ge L, Gan Y-H, Yang K-H. Which is the best Chinese herb injection based on the FOLFOX regimen for gastric cancer? A network meta- analysis of randomized controlled trials. Asian Pac J Cancer Prev. 2014;15(12):4795–800.
82. Wang X, Wang X, Li S, Meng Z, Liu T, Zhang X. Comparative effectiveness of oral drug therapies for lower urinary tract symptoms due to benign prostatic hyperplasia: a systematic review and network meta-Analysis. PLoS One. 2014;9(9):e107593.
83. Wertli MM, Kessels AGH, Perez RSGM, Bachmann LM, Brunner F. Rational pain management in complex regional pain syndrome 1 (CRPS 1)--a network meta-analysis. Pain Med. 2014;15(9):1575–89.
84. Windecker S, Stortecky S, Stefanini GG, da Costa BR, Rutjes AW, Di Nisio M, et al. Revascularisation versus medical treatment in patients with stable coronary artery disease: network meta-analysis. BMJ. 2014;348:g3859. <http://www.bmj.com/cgi/doi/10.1136/bmj.g3859>.
85. Xiong T, Turner RM, Wei Y, Neal DE, Lyratzopoulos G, Higgins JPT. Comparative efficacy and safety of treatments for localised prostate cancer: an application of network meta-analysis. BMJ Open. 2014;4(5):e004285. <http://bmjopen.bmj.com/lookup/doi/10.1136/bmjopen-2013-004285>.
86. Yang B, Shi J, Chen X, Ma B, Sun H. Efficacy and safety of therapies for acute ischemic Stroke in China: a network meta-analysis of 13289 patients from 145 randomized controlled trials. PLoS One. 2014;9(2):e88440.
87. Yang Z, Ye X, Wu Q, Wu K, Fan D. A network meta-analysis on the efficacy of 5-aminosalicylates, immunomodulators and biologics for the prevention of postoperative recurrence in Crohn’s disease. Int J Surg. 2014;12(5):516–22. <http://dx.doi.org/10.1016/j.ijsu.2014.02.010>.
88. Zeng C, Li H, Yang T, Deng ZH, Yang Y, Zhang Y, et al. Effectiveness of continuous and pulsed ultrasound for the management of knee osteoarthritis: a systematic review and network meta-analysis. Osteoarthr Cartil. 2014;22(8):1090–9.
89. Zhang W, Zhang Y, Pan H, Wei F, Zhang Y, Shao Y, et al. Chemotherapy for patients with gastric cancer after complete resection: a network meta-analysis. World J Gastroenterol. 2014;20(2):584–92.
90. Zhu Z, Zhang J, Liu Y, Chen M, Guo P, Li K. Efficacy and toxicity of external-beam radiation therapy for localised prostate cancer: a network meta-analysis. Br J Cancer. 2014;110(10):2396–404. <http://www.nature.com/doifinder/10.1038/bjc.2014.197>.
91. Ziakas PD, Zervou FN, Zacharioudakis IM, Mylonakis E. Graft-versus-host disease prophylaxis after transplantation: a network meta-analysis. PLoS One. 2014;9(12):e114735.
92. Zintzaras E, Miligkos M, Ziakas P, Balk EM, Mademtzoglou D, Doxani C, et al. Assessment of the relative effectiveness and tolerability of treatments of type 2 diabetes mellitus: a network meta-analysis. Clin Ther. 2014;36(10):1443–53.e9. <http://dx.doi.org/10.1016/j.clinthera.2014.06.035>.

**2015**

1. Alfirevic Z, Keeney E, Dowswell T, Welton NJ, Dias S, Jones L V, et al. Labour induction with prostaglandins: a systematic review and network meta-analysis. BMJ. 2015;350:h217.
2. Bateman ED, Esser D, Chirila C, Fernandez M, Fowler A, Moroni-Zentgraf P, et al. Magnitude of effect of asthma treatments on Asthma Quality of Life Questionnaire and Asthma Control Questionnaire scores: systematic review and network meta-analysis. J Allergy Clin Immunol. 2015;136(4):914–22. <http://dx.doi.org/10.1016/j.jaci.2015.03.023>.
3. Benedetto U, Raja SG, Albanese A, Amrani M, Biondi-Zoccai G, Frati G. Searching for the second best graft for coronary artery bypass surgery: a network meta-analysis of randomized controlled trials. Eur J Cardio-thoracic Surg. 2015;47(1):59–65.
4. Blann AD, Skjoth F, Rasmussen LH, Larsen TB, Lip GYH. Edoxaban versus placebo, aspirin, or aspirin plus clopidogrel for stroke prevention in atrial fibrillation: an indirect comparison analysis. Thromb Haemost. 2015;114(2):403–9.
5. Bonsu KO, Reidpath DD, Kadirvelu A. Effects of Statin Treatment on Inflammation and Cardiac Function in Heart Failure: An Adjusted Indirect Comparison Meta-Analysis of Randomized Trials. Cardiovasc Ther. 2015;33(6):338–46.
6. Bow EJ, Vanness DJ, Slavin M, Cordonnier C, Cornely OA, Marks DI, et al. Systematic review and mixed treatment comparison meta-analysis of randomized clinical trials of primary oral antifungal prophylaxis in allogeneic hematopoietic cell transplant recipients. BMC Infect Dis. 2015;15(1):128. <http://www.pubmedcentral.nih.gov/articlerender.fcgi?artid=4374298&tool=pmcentrez&rendertype=abstract>.
7. Brigo F, Nardone R, Tezzon F, Trinka E. A Common Reference-Based Indirect Comparison Meta-Analysis of Buccal versus Intranasal Midazolam for Early Status Epilepticus. CNS Drugs. 2015;29(9):741–57.
8. Buckley F, Finckh A, Huizinga T, Dejonckheere F, Jansen J. Comparative Efficacy of Novel DMARDs as Monotherapy and in Combination with Methotrexate in Rheumatoid Arthritis Patients with Inadequate Response to Conventional DMARDs: A Network Meta-Analysis. J Manag Care Spec Pharm. 2015;21(5):409–23.
9. Bulluck H, Kwok CS, Ryding AD, Loke YK. Safety of short-term dual antiplatelet therapy after drug-eluting stents: An updated meta-analysis with direct and adjusted indirect comparison of randomized control trials. Int J Cardiol. 2015;181:331–9. <http://dx.doi.org/10.1016/j.ijcard.2014.12.037>.
10. Chen L, Staubli SEL, Schneider MP, Kessels AG, Ivic S, Bachmann LM, et al. Phosphodiesterase 5 inhibitors for the treatment of erectile dysfunction: a trade-off network meta-analysis. Eur Urol. 2015;68(4):674–80. <http://dx.doi.org/10.1016/j.eururo.2015.03.031>.
11. Chen YP, Wang ZX, Chen L, Liu X, Tang LL, Mao YP, et al. A Bayesian network meta-analysis comparing concurrent chemoradiotherapy followed by adjuvant chemotherapy, concurrent chemoradiotherapy alone and radiotherapy alone in patients with locoregionally advanced nasopharyngeal carcinoma. Ann Oncol. 2015;26(1):205–11.
12. Dong W, Goost H, Lin X-B, Burger C, Paul C, Wang Z-L, et al. Treatments for shoulder impingement syndrome: a PRISMA systematic review and network meta-analysis. Medicine (Baltimore). 2015;94(10):e510. <http://insights.ovid.com/crossref?an=00005792-201503020-00001>.
13. Dumville J, McFarlane E, Edwards P, Lipp A, Holmes A, Liu Z. Preoperative skin antiseptic for prevention of surgical wound infections after clean surgery. Cochrane Database Syst Rev. 2013;(4):CD003949.
14. Galván-Banqueri M, Vega-Coca MD, Castillo-Muñoz MA, Beltrán Calvo C, Molina López T. Indirect comparison for Anti-TNF drugs in moderate to severe ulcerative colitis. Farm Hosp. 2015;39(2):80–91.
15. Giacoppo D, Gargiulo G, Aruta P, Capranzano P, Tamburino C, Capodanno D. Treatment strategies for coronary in-stent restenosis: systematic review and hierarchical Bayesian network meta-analysis of 24 randomised trials and 4880 patients. BMJ. 2015;351:h5392. <http://www.bmj.com/content/351/bmj.h5392.full.pdf+html>.
16. Greco T, Calabrò MG, Covello RD, Greco M, Pasin L, Morelli A, et al. A Bayesian network meta-analysis on the effect of inodilatory agents on mortality. Br J Anaesth. 2015;114(5):746–56.
17. Gu S, Shi J, Tang Z, Sawhney M, Hu H, Shi L, et al. Comparison of glucose lowering effect of Metformin and acarbose in type 2 diabetes mellitus: a meta-analysis. PLoS One. 2015;10(5):e012670. <http://dx.doi.org/10.1371/journal.pone.0126704>.
18. Guo J-B, Zhu Y, Chen B-L, Xie B, Zhang W-Y, Yang Y-J, et al. Surgical versus non-surgical treatment for vertebral compression fracture with osteopenia: a systematic review and meta-analysis. PLoS One. 2015;10(5):e0127145. <http://dx.plos.org/10.1371/journal.pone.0127145>.
19. Hazlewood GS, Rezaie A, Borman M, Panaccione R, Ghosh S, Seow CH, et al. Comparative effectiveness of immunosuppressants and biologics for inducing and maintaining remission in Crohn’s disease: a network meta-analysis. Gastroenterology. 2015;148(2):344–54. <http://dx.doi.org/10.1053/j.gastro.2014.10.011>.
20. Huai ZY, Feng Xian W, Chang Jiang L, Xi Chen W. Submucosal injection solution for endoscopic resection in gastrointestinal tract: a traditional and network meta-analysis. Gastroenterol Res Pract. 2015;2015:702768. <http://www.hindawi.com/journals/grp/2015/702768/>.
21. Hüttner FJ, Tenckhoff S, Jensen K, Uhlmann L, Kulu Y, Büchler MW, et al. Meta-analysis of reconstruction techniques after low anterior resection for rectal cancer. Br J Surg. 2015;102(7):735–45.
22. Korobelnik J-F, Kleijnen J, Lang SH, Birnie R, Leadley RM, Misso K, et al. Systematic review and mixed treatment comparison of intravitreal aflibercept with other therapies for diabetic macular edema (DME). BMC Ophthalmol. 2015;15:52. <http://bmcophthalmol.biomedcentral.com/articles/10.1186/s12886-015-0035-x>.
23. Kumagai K, Rouvelas I, Tsai JA, Mariosa D, Lind PA, Lindblad M, et al. Survival benefit and additional value of preoperative chemoradiotherapy in resectable gastric and gastro-oesophageal junction cancer: a direct and adjusted indirect comparison meta-analysis. Eur J Surg Oncol. 2015;41(3):282–94.
24. Kunitomi T, Hashiguchi M, Mochizuki M. Effect of common comparators in indirect comparison analysis of the effectiveness of different inhaled Corticosteroids in the treatment of asthma. PLoS One. 2015;10(3):e0120836. <http://dx.doi.org/10.1371/journal.pone.0120836>.
25. Lee SWH, Chaiyakunapruk N, Chong HY, Liong ML. Comparative effectiveness and safety of various treatment procedures for lower pole renal calculi: a systematic review and network meta-analysis. BJU Int. 2015;116(2):252–64.
26. Lee Y, Song G. Relative efficacy and safety of tacrolimus, mycophenolate mofetil, and cyclophosphamide as induction therapy for lupus nephritis: a Bayesian network meta-analysis of randomized controlled trials. Lupus. 2015;24(14):1520–8. <http://www.scopus.com/inward/record.url?eid=2-s2.0-84947274484&partnerID=40&md5=ee8738f54284f2873599a33ce6c915f2>.
27. Lhermusier T, Lipinski MJ, Tantry US, Escarcega RO, Baker N, Bliden KP, et al. Meta-analysis of direct and indirect comparison of ticagrelor and prasugrel effects on platelet reactivity. Am J Cardiol. 2015;115(6):716–23. <http://dx.doi.org/10.1016/j.amjcard.2014.12.029>.
28. Linde K, Kriston L, Rücker G, Jamil S, Schumann I, Meissner K, et al. Efficacy and acceptability of pharmacological treatments for depressive disorders in primary care: systematic review and network meta-analysis. Ann Fam Med. 2015;13(1):69–79.
29. Loveman E, Copley VR, Scott DA, Colquitt JL, Clegg AJ, O’Reilly KM. Comparing new treatments for idiopathic pulmonary fibrosis - a network meta-analysis. BMC Pulm Med. 2015;15:37.
30. Mantha S, Ansell J. Indirect comparison of dabigatran, rivaroxaban, apixaban and edoxaban for the treatment of acute venous thromboembolism. J Thromb Thrombolysis. 2015;39(2):155–65.
31. Messori A, Trippoli S, Fadda V, Maratea D, Marinai C. Subcutaneous Biological Treatments for Moderate to Severe Psoriasis: Interpreting Safety Data by Network Meta-Analysis. Drugs - Real World Outcomes. 2015;2(1):23–7.
32. Messori A, Fadda V, Maratea D, Trippoli S. First-line treatments for chronic lymphocytic leukaemia: interpreting efficacy data by network meta-analysis. Ann Hematol. 2015;94(6):1003–9.
33. Moja L, Danese S, Fiorino G, Del Giovane C, Bonovas S. Systematic review with network meta-analysis: comparative efficacy and safety of budesonide and mesalazine (mesalamine) for Crohn’s disease. Aliment Pharmacol Ther. 2015;41(11):1055–65.
34. Nassar AP, Zampieri FG, Ranzani OT, Park M. Protocolized sedation effect on post-ICU posttraumatic stress disorder prevalence: A systematic review and network meta-analysis. J Crit Care. 2015;30(6):1278–82. <http://dx.doi.org/10.1016/j.jcrc.2015.07.023>.
35. Nelson H, Cartier S, Allen-Ramey F, Lawton S, Calderon MA. Network meta-analysis shows commercialized subcutaneous and sublingual grass products have comparable efficacy. J Allergy Clin Immunol Pr. 2015;3(2):256–266.e3. <http://dx.doi.org/10.1016/j.jaip.2014.09.018>.
36. Oba Y, Lone N. Comparative efficacy of long-acting muscarinic antagonists in preventing COPD exacerbations: a network meta-analysis and meta-regression. Ther Adv Respir Dis. 2015;9(1):3–15. <http://dx.doi.org/10.1177/1753465814565624>.
37. Peruzzi M, De Luca L, Thomsen HS, Romagnoli E, D’Ascenzo F, Mancone M, et al. A network meta-analysis on randomized trials focusing on the preventive effect of statins on contrast-induced nephropathy. Biomed Res Int. 2014;2014:213239.
38. Phan K, Xie A, Kumar N, Wong S, Medi C, La Meir M, et al. Comparing energy sources for surgical ablation of atrial fibrillation: a Bayesian network meta-analysis of randomized, controlled trials. Eur J Cardiothorac Surg. 2015;48(2):201–11. <http://ejcts.oxfordjournals.org/content/48/2/201.long>.
39. Potts JE, Gray LJ, Brady EM, Khunti K, Davies MJ, Bodicoat DH. The effect of glucagon-like peptide 1 receptor agonists on weight loss in type 2 diabetes: A systematic review and mixed treatment comparison meta-analysis. PLoS One. 2015;10(6):e0126769. <http://dx.doi.org/10.1371/journal.pone.0126769>.
40. Rochwerg B, Alhazzani W, Gibson A, Ribic CM, Sindi A, Heels-Ansdell D, et al. Fluid type and the use of renal replacement therapy in sepsis: a systematic review and network meta-analysis. Intensive Care Med. 2015;41(9):1561–71.
41. Schmid MK, Bachmann LM, Fäs L, Kessels AG, Job OM, Thiel MA. Efficacy and adverse events of aflibercept, ranibizumab and bevacizumab in age-related macular degeneration: a trade-off analysis. Br J Ophthalmol. 2015;99(2):141–6. <http://bjo.bmj.com/lookup/doi/10.1136/bjophthalmol-2014-305149>.
42. Scott DA, Woods B, Thompson JC, Clark JF, Hawkins N, Chambers M, et al. Mortality and drug therapy in patients with chronic obstructive pulmonary disease: a network meta-analysis. BMC Pulm Med. 2015;15:145. <http://www.pubmedcentral.nih.gov/articlerender.fcgi?artid=PMC4642642>.
43. Sekine L, Morais V, Lima K, Onsten T, Ziegelmann P, Ribeiro R. Conventional and high-dose daunorubicin and idarubicin in acute myeloid leukaemia remission induction treatment: a mixed treatment comparison meta-analysis of 7258 patients. Hematol Oncol. 2015;33(4):212–9.
44. Sheng Z, Zhang Y. EGFR-TKIs combined with chemotherapy versus EGFR-TKIs single agent as first-line treatment for molecularly selected patients with non-small cell lung cancer. Med Oncol. 2015;32(1):420.
45. Signorovitch JE, Betts KA, Yan YS, Lereun C, Sundaram M, Wu EQ, et al. Comparative efficacy of biological treatments for moderate-to-severe psoriasis: a network meta-analysis adjusting for cross-trial differences in reference arm response. Br J Dermatol. 2015;172(2):504–12.
46. Singh S, Garg S, Pardi D, Wang Z, Murad M, Loftus EJ. Comparative efficacy of pharmacologic interventions in preventing relapse of Crohn’s disease after surgery: a systematic review and network meta-analysis. Gastroenterology. 2015;148(1):64–76.e2.
47. Sobieraj DM, Coleman CI, Pasupuleti V, Deshpande A, Kaw R, Hernandez A V. Comparative efficacy and safety of anticoagulants and aspirin for extended treatment of venous thromboembolism: A network meta-analysis. Thromb Res. 2015;135(5):888–96. <http://dx.doi.org/10.1016/j.thromres.2015.02.032>.
48. Sohn HS, Kwon JW, Shin S, Kim HS, Kim H. Effect of smoking status on progression-free and overall survival in non-small cell lung cancer patients receiving erlotinib or gefitinib: a meta-analysis. J Clin Pharm Ther. 2015;40(6):661–71.
49. Song G-M, Tian X, Shuai T, Yi L-J, Zeng Z, Liu S, et al. Treatment of Adults With Treatment-Resistant Depression: Electroconvulsive Therapy Plus Antidepressant or Electroconvulsive Therapy Alone? Evidence From an Indirect Comparison Meta-Analysis. Medicine. 2015;94(26):e1052. <http://content.wkhealth.com/linkback/openurl?sid=WKPTLP:landingpage&an=00005792-201507010-00035>.
50. Stynes G, Svedsater H, Wex J, Lettis S, Leather D, Castelnuovo E, et al. Once-daily fluticasone furoate/vilanterol 100/25 mcg versus twice daily combination therapies in COPD - mixed treatment comparisons of clinical efficacy. Respir Res. 2015;16:25. <http://www.pubmedcentral.nih.gov/articlerender.fcgi?artid=PMC4339422>.
51. Sun F, Chai S, Li L, Yu K, Yang Z, Wu S, et al. Effects of glucagon-like peptide-1 receptor agonists on weight loss in patients with type 2 diabetes: a systematic review and network meta-analysis. J Diabetes Res. 2015;2015:157201.
52. Sun F, Chai S, Yu K, Quan X, Yang Z, Wu S, et al. Gastrointestinal adverse events of glucagon-like peptide-1 receptor agonists in patients with type 2 diabetes: a systematic review and network meta-analysis. Diabetes Technol Ther. 2015;17(1):35–42. <http://www.pubmedcentral.nih.gov/articlerender.fcgi?artid=PMC4290796>.
53. Sun F, Wu S, Guo S, Yu K, Yang Z, Li L, et al. Effect of GLP-1 receptor agonists on waist circumference among type 2 diabetes patients: a systematic review and network meta-analysis. Endocrine. 2015;48(3):794–803.
54. Sun F, Wu S, Wang J, Guo S, Chai S, Yang Z, et al. Effect of glucagon-like peptide-1 receptor agonists on lipid profiles among type 2 diabetes: a systematic review and network meta-analysis. Clin Ther. 2015;37(1):225–41. <http://dx.doi.org/10.1016/j.clinthera.2014.11.008>.
55. Thakur D, Dickerson S, Bhutani MK, Junor R. Impact of prolonged-release oxycodone/naloxone on outcomes affecting patients’ daily functioning in comparison with extended-release tapentadol: a systematic review. Clin Ther. 2015;37(1):212–24. <http://dx.doi.org/10.1016/j.clinthera.2014.12.001>.
56. Tolley K, Hutchinson M, You X, Wang P, Sperling B, Taneja A, et al. A Network Meta-Analysis of Efficacy and Evaluation of Safety of Subcutaneous Pegylated Interferon Beta-1a versus Other Injectable Therapies for the Treatment of Relapsing-Remitting Multiple Sclerosis. PLoS One. 2015;10(6):e012796. <http://dx.doi.org/10.1371/journal.pone.0127960>.
57. Tramacere I, Del Giovane C, Salanti G, D’Amico R, Filippini G. Immunomodulators and immunosuppressants for relapsing-remitting multiple sclerosis: a network meta-analysis. Cochrane Database Syst Rev. 2015;(9):CD011381.
58. Tvete IF, Natvig B, Gåsemyr J, Meland N, Røine M, Klemp M. Comparing Effects of Biologic Agents in Treating Patients with Rheumatoid Arthritis: A Multiple Treatment Comparison Regression Analysis. PLoS One. 2015;10(9):e0137258.
59. Wang C, Guo L, Chi C, Wang X, Guo L, Wang W, et al. Mechanical ventilation modes for respiratory distress syndrome in infants: a systematic review and network meta-analysis. Crit Care. 2015;19:108. <http://www.pubmedcentral.nih.gov/articlerender.fcgi?artid=4391657&tool=pmcentrez&rendertype=abstract>.
60. Wang L, Baser O, Kutikova L, Page JH, Barron R. The impact of primary prophylaxis with granulocyte colony-stimulating factors on febrile neutropenia during chemotherapy: a systematic review and meta-analysis of randomized controlled trials. Support Care Cancer. 2015;23(11):3131–40.
61. Wang Z, Qiao D, Lu Y, Curtis D, Wen X, Yao Y, et al. Systematic literature review and network meta-analysis comparing bone-targeted agents for the prevention of skeletal-related events in cancer patients with bone metastasis. Oncologist. 2015;20(4):440–9.
62. Yamaguchi N, Fujii T, Aoi S, Kozuch PS, Hortobagyi GN, Blum RH. Comparison of cardiac events associated with liposomal doxorubicin, epirubicin and doxorubicin in breast cancer: a Bayesian network meta-analysis. Eur J Cancer. 2015;51(16):2314–20. <http://dx.doi.org/10.1016/j.ejca.2015.07.031>.
63. Yildiz A, Nikodem M, Vieta E, Correll CU, Baldessarini RJ. A network meta-analysis on comparative efficacy and all-cause discontinuation of antimanic treatments in acute bipolar mania. Psychol Med. 2015;45(2):299–317. <http://www.journals.cambridge.org/abstract_S0033291714001305>.
64. Yu Q, Zhu Z, Liu Y, Zhang J, Li K. Efficacy and Safety of HER2-Targeted Agents for Breast Cancer with HER2-Overexpression: A Network Meta-Analysis. PLoS One. 2015;10(5):e012740. <http://dx.doi.org/10.1371/journal.pone.0127404>.
65. Zeng C, Li H, Yang T, Deng ZH, Yang Y, Zhang Y, et al. Electrical stimulation for pain relief in knee osteoarthritis: systematic review and network meta-analysis. Osteoarthr Cartil. 2015;23(2):189–202.
66. Zhang L, Pang Y, Shi Y, Xu M, Xu X, Zhang J, et al. Indirect comparison of teriparatide, denosumab, and oral bisphosphonates for the prevention of vertebral and nonvertebral fractures in postmenopausal women with osteoporosis. Menopause. 2015;22(9):1021–5.
67. Zhu GQ, Shi KQ, Yu HJ, He SY, Braddock M, Zhou MT, et al. Optimal adjuvant therapy for resected hepatocellular carcinoma: a systematic review with network meta-analysis. Oncotarget. 2015;6(20):18151–61.
68. Zhu GQ, Shi KQ, Huang S, Wang LR, Lin YQ, Huang GQ, et al. Systematic review with network meta-analysis: the comparative effectiveness and safety of interventions in patients with overt hepatic encephalopathy. Aliment Pharmacol Ther. 2015;41(7):624–35.

**2016**

1. Canestaro WJ, Forrester SH, Raghu G, Ho L, Devine BE. Drug Treatment of Idiopathic Pulmonary Fibrosis: Systematic Review and Network Meta-Analysis. Chest. 2016;149(3):756–66. <http://dx.doi.org/10.1016/j.chest.2015.11.013>.
2. Elborn JS, Vataire A-L, Fukushima A, Aballea S, Khemiri A, Moore C, et al. Comparison of Inhaled Antibiotics for the Treatment of Chronic Pseudomonas aeruginosa Lung Infection in Patients With Cystic Fibrosis: Systematic Literature Review and Network Meta-analysis. Clin Ther. 2016;38(10):2204–26. <http://dx.doi.org/10.1016/j.clinthera.2016.08.014>.
3. Firwana B, Sonbol MB, Diab M, Raza S, Hasan R, Yousef I, et al. Tyrosine kinase inhibitors as a first-line treatment in patients with newly diagnosed chronic myeloid leukemia in chronic phase: A mixed-treatment comparison. Int J Cancer. 2016;138(6):1545–53.
4. Freemantle N, Ginsberg DA, Mccool R, Fleetwood K, Arber M, Khalaf K, et al. Comparative assessment of onabotulinumtoxinA and mirabegron for overactive bladder: an indirect treatment comparison. BMJ Open. 2016;6(2):e009122.
5. Kang M, Jeong CW, Kwak C, Kim HH, Ku JH. Single, immediate postoperative instillation of chemotherapy in non-muscle invasive bladder cancer: a systematic review and network meta-analysis of randomized clinical trials using different drugs. Oncotarget. 2016;7(29):45479–88.
6. Kuhr K, Wirth D, Srivastava K, Lehmacher W, Hellmich M. First-line therapy for non-transplant eligible patients with multiple myeloma: direct and adjusted indirect comparison of treatment regimens on the existing market in Germany. Eur J Clin Pharmacol. 2016;72(3):257–65.
7. Li Y, Gao J, He S, Zhang Y, Wang Q. An Evaluation on the Efficacy and Safety of Treatments for Attention Deficit Hyperactivity Disorder in Children and Adolescents: a Comparison of Multiple Treatments. Mol Neurobiol. 2016; doi: 10.1007/s12035-016-0179-6. <http://dx.doi.org/10.1007/s12035-016-0179-6>.
8. Li Z, Chen P, Wang J, Mao Q, Xiang H, Wang X, et al. The impact of surgical treatments for lower urinary tract symptoms/benign prostatic hyperplasia on male erectile function: A systematic review and network meta-analysis. Medicine. 2016;95(24):e3862.
9. Lipinski MJ, Lee RC, Gaglia MA, Torguson R, Garcia-Garcia HM, Pichard AD, et al. Comparison of heparin, bivalirudin, and different glycoprotein IIb/IIIa inhibitor regimens for anticoagulation during percutaneous coronary intervention: A network meta-analysis. Cardiovasc Revascularization Med. 2016;17(8):535–45. <http://dx.doi.org/10.1016/j.carrev.2016.09.011>.
10. Migliore A, Bizzi E, Petrella L, Bruzzese V, Cassol M, Integlia D. The Challenge of Treating Early-Stage Rheumatoid Arthritis: The Contribution of Mixed Treatment Comparison to Choosing Appropriate Biologic Agents. BioDrugs. 2016;30(2):105–15.
11. Miligkos M, Papamichael K, Casteele N Vande, Mantzaris GJ, Gils A, Levesque BG, et al. Efficacy and Safety Profile of Anti-tumor Necrosis Factor-α Versus Anti-integrin Agents for the Treatment of Crohn’s Disease: A Network Meta-analysis of Indirect Comparisons. Clin Ther. 2016;38(6):1342–58.e6. <http://dx.doi.org/10.1016/j.clinthera.2016.03.018>.
12. Park CH, Jung YS, Nam E, Eun CS, Park D Il, Han DS. Comparison of Efficacy of Prophylactic Endoscopic Therapies for Postpolypectomy Bleeding in the Colorectum: A Systematic Review and Network Meta-Analysis. Am J Gastroenterol. 2016;111(9):1230–43.
13. Svedsater H, Stynes G, Wex J, Frith L, Leather D, Castelnuovo E, et al. Once-daily fluticasone furoate/vilanterol 100/25 mcg versus twice daily combination therapies in COPD - mixed treatment comparisons of clinical efficacy. Asthma Res Pr. 2016;2:4. <http://www.pubmedcentral.nih.gov/articlerender.fcgi?artid=PMC4339422>.
14. Xiao J, Wu W, Ye Y, Lin W, Wang L. A Network Meta-analysis of Randomized Controlled Trials Focusing on Different Allergic Rhinitis Medications. Am J Ther. 2015;23(6):e1568–78.

**2017**

1. Chen J, Zhao L, Liu Y, Fan S, Xie P. Comparative efficacy and acceptability of electroconvulsive therapy versus repetitive transcranial magnetic stimulation for major depression: A systematic review and multiple-treatments meta-analysis. Behav Brain Res. 2017;320:30–6. <http://linkinghub.elsevier.com/retrieve/pii/S0166432816308051>.
2. Choi M, Hyun MK, Choi S, Tchoe HJ, Lee SY, Son KM, et al. Comparative efficacy of biological agents in methotrexate-refractory rheumatoid arthritis patients: a Bayesian mixed treatment comparison. Korean J Intern Med. 2017;32(3):536–47. <http://kjim.org/journal/view.php?doi=10.3904/kjim.2015.135>.
3. Devji T, Levine O, Neupane B, Beyene J, Xie F. Systemic Therapy for Previously Untreated Advanced BRAF-Mutated Melanoma: A Systematic Review and Network Meta-Analysis of Randomized Clinical Trials. JAMA Oncol. 2017;3(3):366–73. <http://oncology.jamanetwork.com/article.aspx?doi=10.1001/jamaoncol.2016.4877>.
4. Htike ZZ, Zaccardi F, Papamargaritis D, Webb DR, Khunti K, Davies MJ. Efficacy and safety of glucagon-like peptide-1 receptor agonists in type 2 diabetes: A systematic review and mixed-treatment comparison analysis. Diabetes, Obes Metab. 2017;19(4):524–36.
5. Kim SJ, Shin IS, Eun SJ, Whangbo TK, Kim JW, Cho YS, et al. Evidence Is Enough?: A Systematic Review and Network Meta-Analysis of the Efficacy of Tamsulosin 0.2 mg and Tamsulosin 0.4 mg as an Initial Therapeutic Dose in Asian Benign Prostatic Hyperplasia Patients. Int Neurourol J. 2017;21(1):29–37.
6. Nelson AD, Camilleri M, Chirapongsathorn S, Vijayvargiya P, Valentin N, Shin A, et al. Comparison of efficacy of pharmacological treatments for chronic idiopathic constipation: a systematic review and network meta-analysis. Gut. 2017;66(9):1611–22.
7. Obloza A, Kirby J, Yates DJ, Toozs-Hobson P. Indirect treatment comparison (ITC) of medical therapies for an overactive bladder. Neurourol Urodyn. 2017;36(7):1824–31.
8. Rihn J, Radcliff K, Norvell D, Eastlack R, Phillips F, Berland D, et al. Comparative Effectiveness of Treatments for Chronic Low Back Pain: A Multiple Treatment Comparison Analysis. Clin Spine Surg. 2017;30(5):204–25.
9. Singh JA, Hossain A, Mudano AS, Tanjong Ghogomu E, Suarez-Almazor ME, Buchbinder R, et al. Biologics or tofacitinib for people with rheumatoid arthritis unsuccessfully treated with biologics: a systematic review and network meta-analysis. Cochrane Database Syst Rev. 2017;3:CD012591.
10. Wang X, Zheng H, Shou T, Tang C, Miao K, Wang P. Effectiveness of multi-drug regimen chemotherapy treatment in osteosarcoma patients: a network meta-analysis of randomized controlled trials. J Orthop Surg Res. 2017;12(1):52. <http://josr-online.biomedcentral.com/articles/10.1186/s13018-017-0544-9>.
11. Zhang T, Zhang S, Yang F, Wang L, Zhu S, Qiu B, et al. Efficacy Comparison of Six Chemotherapeutic Combinations for Osteosarcoma and Ewing’s Sarcoma Treatment: A Network Meta-Analysis. J Cell Biochem. 2017;doi: 10.1002/jcb.25976.
