# Supplementary material for: A systematic survey shows that reporting and handling of missing outcome data in networks of interventions is poor
Source: BMC Med Res Methodol. 2018 Oct 24;18:115. doi: 10.1186/s12874-018-0576-9 (PMC6201503; doi:10.1186/s12874-018-0576-9)
Supplement: Supplementary file 3 — Appendix C. List of verbatim definitions of missing outcome data. (DOCX 23 kb) [file 12874_2018_576_MOESM3_ESM.docx]

**Appendix C.** List of verbatim definitions of missing outcome data^†^

| **ID** | **Found in** | **Quotation** |
| --- | --- | --- |
| 1 | **Outcome measures** (*p. 747*) | ‘[...] we defined treatment discontinuation (acceptability) as the number of patients who terminated the study early for any reason during the first 8 weeks of treatment (dropouts).’ |
| 2 | **Study Selection** (*p. 893*) | ‘[…] the proportion of patients withdrawing from the trial. This included, but was not limited to, withdrawals due to adverse events, treatment failure, and disease progression.’ |
| 3 | **Discussion** (*p. 431*) | ‘All trials of biologic agents allowed patients who did not respond to placebo to withdraw before the primary endpoint (week 24).’ |
| 4 | **Data collection and study quality assessment** (*p. 4*) | ‘[no] completeness of follow-up, […]’ |
| 5 | **Systematic review of the literature** (*p. 267*) | ‘[…] patients who discontinued therapy for any reason or received rescue therapies were considered as non-responders.’ |
| 6 | **Abstract** (*p. 128*) | ‘[…] or withdrawal of patients because of edema […]’ |
| 7 | **Primary outcomes** (*p. 1281*) | ‘[…] withdrawal rate (proportion withdrawing from treatment during treatment period).’ |
| 8 | **Outcome measures** (*p. 1307*) | ‘Treatment discontinuation (acceptability) was defined as the number of patients who left the study early for any reason during the first 3 weeks of treatment of the total number of patients randomly assigned to each treatment group.’ |
| 9 | **Primary outcomes** (*p. 18*) | ‘Treatment discontinuation was used to assess acceptability and was measured by the dropout rate, that is the proportion of participants who were lost to follow-up or definitely discontinued treatment (withdrawals) but completed follow-up, out of the total number of participants randomly assigned to each treatment arm.’ |
| 10 | **Meta-analysis**  (*p. 4*) | ‘[…] patients that dropped out before the first or second visit […] – Protocol^‡^ |

**^†^**The remaining systematic reviews used a similar definition to describe missing outcome data.

^‡^Kriston L, von Wolff A, Hölzel L. Effectiveness of psychotherapeutic, pharmacological, and combined treatments for chronic depression: a systematic review (METACHRON). BMC Psychiatry 2010;10:95.

**References**

1. Cipriani A, Furukawa TA, Salanti G, Geddes JR, Higgins JP, Churchill R, et al. Comparative efficacy and acceptability of 12 new-generation antidepressants: a multiple-treatments meta-analysis. Lancet 2009;373:746-58.
2. Baker WL, Baker EL, Coleman CI. Pharmacologic treatments for chronic obstructive pulmonary disease: a mixed-treatment comparison meta-analysis. Pharmacotherapy 2009;29:891-905.
3. Bergman GJ, Hochberg MC, Boers M, Wintfeld N, Kielhorn A, Jansen JP. Indirect comparison of tocilizumab and other biologic agents in patients with rheumatoid arthritis and inadequate response to disease-modifying antirheumatic drugs. Semin Arthritis Rheum 2010;39:425-41.
4. Middleton LJ, Champaneria R, Daniels JP, Bhattacharya S, Cooper KG, Hilken NH, et al. Hysterectomy, endometrial destruction, and levonorgestrel releasing intrauterine system (Mirena) for heavy menstrual bleeding: systematic review and meta-analysis of data from individual patients. BMJ 2010;341:c3929.
5. Salliot C, Finckh A, Katchamart W, Lu Y, Sun Y, Bombardier C, et al. Indirect comparisons of the efficacy of biological antirheumatic agents in rheumatoid arthritis in patients with an inadequate response to conventional disease-modifying antirheumatic drugs or to an anti-tumour necrosis factor agent: a meta-analysis. Ann Rheum Dis 2011;70:266-71.
6. Makani H, Bangalore S, Romero J, Wever-Pinzon O, Messerli FH. Effect of renin-angiotensin system blockade on calcium channel blocker-associated peripheral edema. Am J Med 2011;124:128-35.
7. Costa J, Fareleira F, Ascenção R, Borges M, Sampaio C, Vaz-Carneiro A. Clinical comparability of the new antiepileptic drugs in refractory partial epilepsy: a systematic review and meta-analysis. Epilepsia 2011;52:1280-91.
8. Cipriani A, Barbui C, Salanti G, Rendell J, Brown R, Stockton S, et al. Comparative efficacy and acceptability of antimanic drugs in acute mania: a multiple-treatments meta-analysis. Lancet 2011;378:1306-15.
9. Filippini G, Del Giovane C, Vacchi L, D'Amico R, Di Pietrantonj C, Beecher D, et al. Immunomodulators and immunosuppressants for multiple sclerosis: a network meta-analysis. Cochrane Database Syst Rev 2013;(6):CD008933.
10. Kriston L, von Wolff A, Westphal A, Hölzel LP, Härter M. Efficacy and acceptability of acute treatments for persistent depressive disorder: a network meta-analysis. Depress Anxiety 2014;31:621-30.
