# Supplementary material for: A systematic survey shows that reporting and handling of missing outcome data in networks of interventions is poor
Source: BMC Med Res Methodol. 2018 Oct 24;18:115. doi: 10.1186/s12874-018-0576-9 (PMC6201503; doi:10.1186/s12874-018-0576-9)
Supplement: Supplementary file 4 — Appendix D. Description of intention-to-treat analyses according to reports in the reviews. (DOCX 35 kb) [file 12874_2018_576_MOESM4_ESM.docx]

**Appendix D.** Description of intention-to-treat analyses according to reports in reviews

| **PMID** | **Year** | **Quotation/explanation** |
| --- | --- | --- |
| **Data extracted *as reported* in the trials** | | |
| 20847017 | 2010 | 'Whenever possible, we used results of intention to treat analysis including all randomised patients'. |
| 21097801 | 2011 | 'All trials performed an ITT analysis and patients who discontinued therapy for any reason or received rescue therapies were considered as non-responders'. |
| 21224324 | 2011 | 'Whenever possible we used results from intention to treat analysis of the longest follow-up available'. |
| 21398351 | 2011 | 'Analyses were performed on data that were explicitly reported in the individual papers, with no imputations for data that were not reported. When available, we analysed the intention to treat population; when this was not possible, we used data from the last observation carried forward'. |
| 22999811 | 2012 | 'Whenever possible, we used results from intention-to-treat analysis'. |
| 23013610 | 2012 | 'Results from intention-to-treat (ITT) analyses were preferred'. |
| 23102749 | 2012 | 'The primary efficacy endpoint was clinical success at test of cure in the following intention-to-treat (ITT) populations: the modified ITT (MITT) population […]'. |
| 22739992 | 2013 | 'Whenever available we used results from intention-to-treat analyses'. |
| 23325883 | 2013 | 'They extracted key characteristics of studies and patients, including the following outcomes, reported at the longest available follow-up according to intention to treat principles'. |
| 23334780 | 2013 | Appendix B indicates that the authors seem to have collected the data as intention-to-treat, where possible. |
| 23718547 | 2013 | 'All analyses were based on the intention-to-treat (ITT) data reported in the manuscripts published for the included RCTs'. |
| 23723055 | 2013 | 'Outcomes were extracted preferentially by intention-to-treat method'. |
| 23723742 | 2013 | 'Results from intention-to-treat analysis were preferred over results from completer analyses'. |
| 23734288 | 2013 | 'Key study and patient characteristics were extracted, including the following outcomes, reported at the longest available follow-up according to intention-to-treat principles […]'. |
| 23810019 | 2013 | 'Intention-to-treat datasets were used whenever available (for continuous and binary outcomes)'. |
| 24043936 | 2013 | 'For the dichotomous outcomes (% of SGRQ and TDI responders), the number of responders was extracted, if reported, or calculated, based on the reported percentage and the ITT population'. |
| 24055922 | 2013 | 'If possible we used the intention to treat population for analyses'. |
| 24166666 | 2013 | 'The occurrence of the following end point(s) was abstracted according to the intention-to-treat population for individual trials and separately for the study drug and control drug(s)'. |
| 23768246 | 2014 | 'ITT values were also used whenever possible'. |
| 24009224 | 2014 | 'We used data from the intention-to-treat populations, unless otherwise specified'. |
| 24444390 | 2014 | Table 1 indicates that the authors seem to have collected the data as intention-to-treat. |
| 24506179 | 2014 | 'Of note, for each study, we used the number of patients randomised for the intention-to-treat sample size'. |
| 24576593 | 2014 | 'Whenever possible we used results from intention-to-treat analysis'. |
| 24587319 | 2014 | 'Outcomes were extracted preferentially by intention to treat method'. |
| 24671923 | 2014 | 'When possible, we used data from intention-to-treat (ITT) analyses from all randomly assigned participants'. |
| 24892649 | 2014 | 'Studies needed to report intention to treat (ITT) or full analysis set (FAS) data, not only per-protocol (PP) data'. |
| 25006351 | 2014 | 'All meta-analyses were conducted on a modified intent-to-treat population, which is defined as the set of patients who were randomised and received at least 1 dose of study medication'. |
| 25029255 | 2014 | In the legend of Table 1 it is stated that 'Analysis is based on the intention-to-treat population of each study' and in the Risk of Bias assessment all trials are reported to have applied intention-to-treat analysis. |
| 25066766 | 2014 | '[…] and results from intention-to-treat (ITT) analyses that included all randomized patients took precedence over results from analyses that excluded patients'. |
| 25234478 | 2014 | 'Whenever available, we used results from the intention-to-treat analysis'. |
| 25348757 | 2014 | 'The data extracted included […] and intention-to-treat end-point rates'. |
| 25361361 | 2014 | 'Only data from the intention-to-treat analysis of each study were included in this analysis'. |
| 25435923 | 2014 | 'The information about the endpoint achievement was aimed to reflect the intention-to-treat population. However, there were some occasional post-randomization exclusions in some trials, and so the clinical material actually adopted the so-called modified intention-to-treat population'. |
| 25036226 | 2015 | 'Intention-to-treat (ITT) datasets were used whenever available'. |
| 25263803 | 2015 | 'The denominator used in all trials was based on a modified intention-to-treat (mITT) analysis, that is, only data on patients who had at least one endoscopic and/or clinical assessment on follow-up was extracted'. |
| 25271911 | 2015 | 'Results from intention to treat analysis were considered wherever possible'. |
| 25448924 | 2015 | 'Data were extracted on a basis of intention-to-treat analysis'. |
| 25491892 | 2015 | 'Survival data were requested for the intention-to-treat population recruited from each trial'. |
| 25652947 | 2015 | 'Whenever possible we used results from intention-to-treat population'. |
| 26537292 | 2015 | 'Data used in this meta-analysis were intention to treat. Most of the included trials did not report as treated results'. |
| 26559138 | 2015 | 'The base case analysis included all RCTs meeting our inclusion criteria using the intention to treat (ITT) results from these studies […]'. |
| 26455714 | 2016 | 'The number of events in each trial was extracted, when available, on the basis of the intention-to-treat approach'. |
| 26671239 | 2016 | '[…] data on benefits and risks must be extractable according to the intention-to-treat (ITT) principle'. |
| 27692977 | 2016 | 'Data for both continuous and binary outcomes were extracted based on the number of patients included in the analysis. Where possible, reported findings were based on the full analysis set'. |
| 27981757 | 2017 | 'Data were extracted according to the intention-to-treat principle'. |
| 28361519 | 2017 | '[…] intention-to-treat (ITT) analysis were performed in RCTs'. |
| **Intention-to-treat analysis applied *de novo*** | | |
| 19185342 | 2009 | 'Responders to treatment were calculated on an intention-to-treat basis […]'. |
| 21040531 | 2010 | 'Imputed data for the base-case analysis was derived from the intention-to-treat (ITT) population and used the last observation carried forward (LOCF) method'. |
| 19965836 | 2011 | 'When not available, the number of patients post-surgery was assumed to be identical to the number of patients at baseline, i.e. assuming there was no loss to follow up'. |
| 21123111 | 2011 | 'The intention-to-treat meta-analysis was done in line with recommendations from the Cochrane Collaboration […]'. |
| 21227948 | 2011 | 'Data were analysed using an intention-to-treat (ITT) approach, and were based on all data on patients allocated to an intervention, including patients who withdrew from the trial and those who did not complete treatment'. |
| 21535970 | 2011 | 'Statistical analysis will be carried out on all the patients ever randomised, and will be based on the ITT principle'. |
| 21851976 | 2011 | 'Analysis was done by intention to treat (using the all missing failures assumption).' |
| 22020356 | 2011 | 'We calculated the rate of responders in each study arm as the number of patients with a certain degree of improvement divided by the total number of patients in the group (intention-to-treat analysis) […]'. |
| 22516128 | 2012 | '[…] one in which patients lost to follow-up were considered cures and one in which they were considered failures'. |
| 22586281 | 2012 | 'An intention-to-treat meta-analysis was performed in line with recommendations from the Cochrane Collaboration […]'. |
| 22700784 | 2012 | 'We carried out […] on an intention to treat basis, according to PRISMA […]'. |
| 23529608 | 2013 | 'All analyses were based on the total number of randomly assigned participants regardless of whether the study authors perform intention-to-treat analyses'. |
| 23658937 | 2013 | 'We calculated rates using the number of all randomized patients as the denominator to reflect a true intention-to-treat analysis'. |
| 23744561 | 2013 | '[…] we assumed that the treated- and control-group participants who dropped out and were not included in the study analysis both had the outcome (relapse or disability progression)'. |
| 23996149 | 2013 | 'Outcomes were allocated according to the intention to treat principle'. |
| 24118976 | 2013 | 'We included data on all randomized patients, regardless of treatment completion. Data concerning only those patients who completed the assigned treatment (“per protocol”) was not available for most included studies and could therefore not be evaluated'. |
| 24212107 | 2013 | 'We did intention to treat direct comparison meta-analysis in line with recommendations from the Preferred Reporting Items for Systematic Reviews […]'. |
| 24618328 | 2014 | 'Missing stiffness subscale scores were imputed by substituting the mean of those reported for that treatment'. |
| 24697518 | 2014 | 'Intention-to-treat analyses were based on the total number of randomly assigned participants, irrespective of how the original study investigators analysed the data, by assuming all drop-outs to be non-responders'. |
| 24736585 | 2014 | 'The number of patients was calculated on an intention-to-treat basis: the analysis of efficacy data was based on the total number of randomly assigned participants, regardless of how the investigators of the original study analysed the data'. |
| 24831822 | 2014 | 'Drop-outs or those patients lost to follow-up are treated as continuing smokers'. |
| 24939927 | 2014 | 'Intention-to-treat direct comparison meta-analysis was performed in line with recommendations from the PRISMA […]'. |
| 25188312 | 2014 | '[…] (intention-to-treat [ITT] populations, Missing/Non-Completers = Failure)'. |
| 25226478 | 2014 | 'Outcomes were allocated according to the intention-to-treat principle'. |
| 25242076 | 2014 | 'Analyses were conducted following the principles of intention to treat, with all events analysed according to the participant’s original randomised treatment allocation, irrespective of deviation based on non-concordance. |
| 25389143 | 2014 | 'Intention-to-treat meta-analyses were performed in line with recommendations from the Cochrane Collaboration […]'. |
| 25441399 | 2014 | 'The denominator used in all trials was based on intention-to-treat analysis (i.e., all dropouts were assumed to be treatment failures), when available'. |
| 25583895 | 2015 | 'Patients with missing data were considered non-responders or non-remitters. In cases where responder and remission data were not reported, we imputed it from available score data'. |
| 25864873 | 2015 | 'The intention-to-treat principle was adopted: analysis was based on the total number of randomly assigned participants, irrespective of how the original study investigators analysed the data'. |
| 27876667 | 2017 | 'To perform a clinically sound analysis, we used a worst-case scenario analysis of drop-out patients, assuming all such patients failed to respond to treatment'. |
| **Combination of *as reported* and *de novo* intention-to-treat analysis** | | |
| 19558609 | 2009 | '[…] extracted and re-calculated if not analysed by intention-to-treat.' (For some trial, the outcomes were reported as intention-to-treat, and for other, they were re-calculated as intention-to-treat). |
| 20934984 | 2011 | 'Where studies did not report intent-to-treat, we analyzed outcomes as all-patients randomized'. |
| 21487451 | 2011 | 'We extracted data on the number of exacerbations per patient arm and calculated total patient years at risk as reported in the article, or if unavailable, by the intention to treat principal […]'. |
| 21678632 | 2011 | 'We recorded intention-to-treat results when reported. If true intention-to-treat results were not reported, but loss to follow-up was very small, we considered these results to be intention-to-treat results. In cases where only per protocol results were reported, we calculated intention-to-treat results if the data for these calculations were available'. |
| 21920996 | 2012 | '[…] according to the number of events reported in the original studies or sub-studies intent-to-treat analyses. Where studies did not report intent to treat, we analyzed outcomes as all-patients randomized'. |
| 22107456 | 2012 | 'We used data from intention-to-treat analyses, […] (where ITT data were not available) missing values were imputed as virological failure and no increase in CD4 cell count from baseline'. |
| 23020934 | 2012 | '[…] according to the number of events reported in the original studies or sub-studies intent-to-treat analysis. Where studies did not report intent-to-treat, we analyzed outcomes as all-patients randomized'. |
| 23365557 | 2012 | '[…] according to the number of events reported in the original studies or sub-studies intent-to-treat analysis. Where studies did not report intent-to-treat, we analyzed outcomes as all-patients randomized'. |
| 24284258 | 2013 | 'Data from intention-to-treat (ITT) analyses were extracted. […] Should a trial not report ITT data then missing data were treated as treatment failures to allow the analysis to conform to an ITT analysis'. |
| 24448972 | 2014 | 'For trials including participants with missing data, intention-to-treat (ITT) estimates were used as reported by the trial authors. If ITT estimates were unavailable, participants with missing data were considered non-responders'. |
| 25115635 | 2015 | 'For studies that did not report intention to treat, we analyzed outcomes as all patients randomized'. |
| 25554560 | 2015 | 'For studies that did not report intention-to-treat analyses, outcomes were analyzed as all-patients randomized'. |
| 25688373 | 2015 | 'Where studies did not report intent-to-treat, we analyzed outcomes as all-patients randomized'. |
| **Unclear classification** | | |
| 19954682 | 2009 | 'Analyses were carried out for the intention to treat (ITT)'. |
| 21295192 | 2011 | 'All analyses were performed using the intention-to-treat principle'. |
| 21831256 | 2012 | 'All the results of the included studies were analysed on an intention-to-treat (ITT) basis'. |
| 22387582 | 2012 | 'The analyses were conducted on an intent-to-treat (ITT) basis for the outcomes of bleeding'. |
| 22885395 | 2012 | 'In addition, all analyses were performed with an intention to treat principle'. |
| 23042705 | 2013 | 'We used the intention-to-treat analysis'. |
| 23519234 | 2013 | 'The main analysis was on an ITT'. |
| 23550994 | 2013 | '[…] we analysed the data based on intention-to-treat analyses, when possible'. |
| 23886301 | 2013 | '[…] relative treatment effects for OS, PFS, TTP and survival risk at years 1 and 2 were estimated using a standard meta-analysis for head-to-head comparisons between interventions based on intention-to-treat analyses.' (No information on whether intention-to-treat is as reported or de novo). |
| 23899710 | 2013 | 'Our analysis was *based on* intention-to-treat principle.' (No information on whether intention-to-treat is as reported or de novo). |
| 24196498 | 2013 | 'Data were analysed according to the intention to treat principle'. |
| 24617578 | 2014 | 'The main analysis was on an intention to treat basis'. |
| 24661896 | 2014 | ' […] outcomes […] analysed strictly on an intention-to-treat basis'. |
| 24842416 | 2014 | 'The outcome measures […] calculated in accordance with the intention-to-treat principle'. |
| 24929780 | 2014 | 'The appropriate data for the primary comparison of PFS were considered to be the total intention-to-treat EGFR mutation-positive population (total population)' (no information on whether they extracted the data as reported or they did intention-to-treat de novo). |
| 25068763 | 2014 | 'Data were analyzed using an intention to treat model'. |
| 25555272 | 2015 | 'The main analysis was on intention to treat basis […]'. |
| 25993646 | 2015 | 'The intent-to-treat analysis was used for the evaluation of the OSR, ORR and side-effects'. |
| 26293745 | 2015 | 'For each outcome, an intention-to-treat primary analysis was made to include all patients in the treatment group to which they were allocated, irrespective of the treatment they actually received'. |
| 26356639 | 2015 | 'We wanted […] to follow the intention to treat principle'. |
