# Supplementary material for: A systematic survey shows that reporting and handling of missing outcome data in networks of interventions is poor
Source: BMC Med Res Methodol. 2018 Oct 24;18:115. doi: 10.1186/s12874-018-0576-9 (PMC6201503; doi:10.1186/s12874-018-0576-9)
Supplement: Supplementary file 5 — Appendix E. Tools used for the risk of bias assessment in 387 systematic reviews. (DOCX 49 kb) [file 12874_2018_576_MOESM5_ESM.docx]

**Appendix E. Tools used for the risk of bias assessment in 387 systematic reviews**

1. **Critical Appraisal Skills Programme (CASP)**

Critical Appraisal Skills Programme (2017). CASP Checklist. Available at: <http://www.casp-uk.net/checklists>.

1. **Cochrane Risk of Bias Tool (CRoB)**

Higgins JP, Altman DG, Gøtzsche PC, Jüni P, Moher D, Oxman AD, Savovic J, Schulz KF, Weeks L, Sterne JA; Cochrane Bias Methods Group; Cochrane Statistical Methods Group. The Cochrane Collaboration's tool for assessing risk of bias in randomised trials. BMJ. 2011;343:d5928.

1. **Centre for Reviews and Dissemination (CRD)**

Centre for Reviews and Dissemination. Undertaking systematic reviews of research on effectiveness: CRD’s guidance for those carrying out or commissioning reviews, 2nd edn. York: CRD, University of York, 2001.

Centre for Reviews and Dissemination (CRD). Manual for selecting reviews and writing abstracts for the Database of Abstracts of Reviews of Effects (DARE). 2008. URL: [www.crd.york.ac.uk/crdweb/html/help.htm](http://www.crd.york.ac.uk/crdweb/html/help.htm).

Centre for Reviews and Dissemination (CRD). Systematic reviews: CRD’s guidance for undertaking reviews in health care. 3rd edn. London: CRD; 2009.

1. **The Delphi list (Delphi)**

Verhagen AP, de Vet HC, de Bie RA, et al. The Delphi list: a criteria list for quality assessment of randomized clinical trials for conducting systematic reviews developed by Delphi consensus. J Clin Epidemiol 1998;51:1235–41.

1. **Evidence-Based Gastroenterology Steering Group (EBGSG)**

Schoenfeld P, Cook D, Hamilton F, Laine L, Morgan D, Peterson W. An evidence-based approach to gastroenterology therapy. Evidence-Based Gastroenterology Steering Group. Gastroenterology 1998; 114: 1318–25.

1. **Institut für Qualität und Wirtschaftlichkeit im Gesundheitswesen (IQWiG)**

Institut für Qualität und Wirtschaftlichkeit im Gesundheitswesen (IQWiG). Allgemeine Methoden (Version 4.0); 2011.

1. **Jadad scale**

Jadad AR, Moore RA, Carroll D, Jenkinson C, Reynolds DJ, Gavaghan DJ, McQuay HJ. Assessing the quality of reports of randomized clinical trials: is blinding necessary? Control Clin Trials. 1996;17(1):1-12.

1. **Quality of controlled clinical trials (Jüni)**

Juni P, Altman DG, Egger M. Systematic reviews in health care: assessing the quality of controlled clinical trials. BMJ 2001;323:42–6.

1. **National Institute for Health and Care Excellence (NICE)**

Centre for Reviews and Dissemination. Systematic reviews. CRD’s guidance for undertaking reviews in health care. York: Centre for Reviews and Dissemination; 2008. Available at: <http://www.york.ac.uk/inst/crd/pdf/Systematic_Reviews.pdf>. Accessed September 4, 2012.

Single technology appraisal (STA): specification for manufacturer/sponsor submission of evidence. London: National Institute For Health And Clinical Excellence; 2009.

The National Institute for Health and Care Excellence (2012) The Guidelines Manual 2012. Available: <http://www.nice.org.uk/aboutnice/howwework/developingniceclinicalguidelines/clinicalguidelinedevelopmentmethods/clinical_guideline_development_methods.jsp>.

National Institute for Health and Care Excellence. Single technology appraisal. Specification for manufacturer/sponsor submission of evidence; June 2012. nsor submission of evidence. Accessed August 12, 2012.

National Institute for Health and Care Excellence (NICE). Process and methods guides. The guidelines manual: appendices B -I. Published: 30 November 2012. Appendix C: Methodological Checklist: randomised controlled trials.

Mirabegron for treating symptoms of overactive bladder. NICE technology appraisal guidance 290. 2013. guidance.nice.org.uk/ta290. (accessed 4 April 2015).

1. **Empirical evidence of bias (Schulz)**

Schulz KF, Chalmers I, Hayes RJ, Altman DG. Empirical evidence of bias. Dimensions of methodological quality associated with estimates of treatment effects in controlled trials. JAMA 1995; 273:408–12.

1. **Scottish Intercollegiate Guidelines Network (SIGN50)**

Harbour R, Miller J. A new system for grading recommendations in evidence based guidelines. BMJ 2001, 323:334-336.

Scottish Intercollegiate Guidelines Network (SIGN). SIGN 50: a guideline developer’s handbook. Edinburgh: SIGN; 2014. (SIGN publication no. 50). [October 2014]. Available from URL: <http://www.sign.ac.uk>.

1. **Other tools (Other)**

Armijo-Olivo, S., Stiles, C.R., Hagen, N.A., Biondo, P.D., Cummings, G.G. Assessment of study quality for systematic reviews: a comparison of the Cochrane Collaboration Risk of Bias Tool and the Effective Public Health Practice Project Quality Assessment tool: methodological research. J Eval Clin Pract, 2012;18(1):12-8.

Australian Pharmaceutical Benefits Advisory Committee (PBAC) Indirect Comparisons Working Group. Report of the indirect comparisons working group to the pharmaceutical benefits advisory committee: assessing indirect comparisons. Canberra: Australian Department of Health and Ageing, PBAC, 2009.

G-BA. The benefit assessment of pharmaceuticals in accordance with the German Social Code, Book Five (SGB V), section 35a. 2014.

HAS. Analysis of the literature and recommendations grading guide. 2000.

Risk of bias assessment criteria for a surgical RCT: Parker MJ, Gurusamy K. Arthroplasties (with and without bone cement) for proximal femoral fractures in adults. Cochrane Database Syst Rev. 2004;(2):CD001706.

van Tulder MW, Furlan A, Bombardier C, Bouter L; Editorial Board of the Cochrane Collaboration Back Review Group. Updated method guidelines for systematic reviews in the cochrane collaboration back review group. Spine (Phila Pa 1976). 2003;28(12):1290-9.

Heart Collaborative Review Group: Villanueva EV, Wasiak J, Petherick ES. Percutaneous transluminal rotational atherectomy for coronary artery disease. Cochrane Database Syst Rev. 2003;(4):CD003334.

Viswanathan M, Ansari MT, Berkman ND, et al. Assessing the Risk of Bias of Individual Studies in Systematic Reviews of Health Care Interventions Methods Guide for Comparative Effectiveness Reviews. AHRQ Publication No. 12-EHC047-EF. Rockville, MD: Agency for Healthcare Research and Quality; March 2012. [www.effectivehealthcare.ahrq.gov/](http://www.effectivehealthcare.ahrq.gov/).

Wright JG, Swiontkowski MF, Heckman JD. Introducing levels of evidence to the journal. J Bone Joint Surg Am. 2003;85-A:1–3.

Woolacott N, Hawkins N, Mason A, et al. Etanercept and efalizumab for the treatment of psoriasis: a systematic review: Appendix 2. Health Technol Assess 2006;10(46):1-233, i-iv.

Wood L, Egger M, Gluud LL, Schulz KF, Jüni P, Altman DG, Gluud C, Martin RM, Wood AJ, Sterne JA (2008). Empirical evidence of bias in treatment effect estimates in controlled trials with different interventions and outcomes: meta-epidemiological study. British Medical Journal 336, 601–605.

1. **Combination of tool tools**

Combination of ‘Jadad scale’ and ‘Cochrane Risk of Bias Tool’.

Combination of ‘Jadad scale’ and ‘NICE Single technology appraisal’.

Combination of ‘Quality of controlled clinical trials (Jüni, 2001)’ and ‘Wood L, Egger M, Gluud LL, Schulz KF, Jüni P, Altman DG, Gluud C, Martin RM, Wood AJ, Sterne JA (2008). Empirical evidence of bias in treatment effect estimates in controlled trials with different interventions and outcomes: meta-epidemiological study. British Medical Journal 336, 601–605’.

Combination of ‘Jadad scale’ with ‘HAS. Analysis of the literature and recommendations grading guide. 2000’; ‘G-BA. The benefit assessment of pharmaceuticals in accordance with the German Social Code, Book Five (SGB V), section 35a. 2014’ and ‘NICE Single technology appraisal’.

Combination of ‘Jadad scale’ with ‘NICE assessment criteria’; ‘Cochrane Risk of Bias Tool’ and ‘IQWiG guidelines’.

1. **Wrong tool**

Shea BJ, Grimshaw JM, Wells GA, Boers M, Andersson N, et al. (2007) Development of AMSTAR: a measurement tool to assess the methodological quality of systematic reviews. BMC medical research methodology 7: 10.
