# Supplementary material for: A systematic survey shows that reporting and handling of missing outcome data in networks of interventions is poor
Source: BMC Med Res Methodol. 2018 Oct 24;18:115. doi: 10.1186/s12874-018-0576-9 (PMC6201503; doi:10.1186/s12874-018-0576-9)
Supplement: Supplementary file 6 — Appendix F. Examples of systematic reviews with explicit, implicit and unclear judgments. (DOCX 57 kb) [file 12874_2018_576_MOESM6_ESM.docx]

**Appendix F. Examples of systematic reviews with explicit, implicit and unclear judgments**


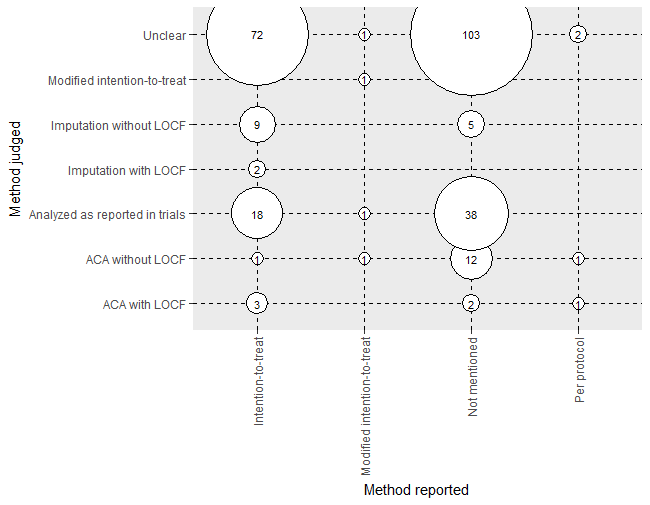


**Figure.** Bubble chart of 273 selected network meta-analyses by method reported and method judged.

**Table.** Examples of systematic reviews with explicit, implicit and unclear judgments

| **PMID** | **Year** | **Quotations/Explanations** |
| --- | --- | --- |
| **Explicit judgment of the actual method** | | |
| ***Available case analysis with or without LOCF*** | | |
| 20223500 | 2010 | 'If not otherwise stated, imputation for nonresponse was assumed to be through last observation carried forward'. |
| 20713583 | 2010 | 'There was a general lack of true intention to treat analysis, with some studies stating that an intention to treat had been performed yet analysing only those women who had received treatment. For four studies that reported per protocol analyses, intention to treat analyses were undertaken with the available data on individual patients, although it was not always clear if patients who deviated from protocol were followed up correctly in these cases'. |
| 21040531 | 2010 | They consider as intention-to-treat the use of LOCF: 'Imputed data for the base-case analysis was derived from the intention-to-treat (ITT) population and used the last observation carried forward (LOCF) method'. |
| 20887240 | 2011 | 'We observe that, for all but EOP 1003 and EOP 1004 studies, one important limitation was that we could extract data reported with the Last Observation Carried Forward (LOCF) technique to take into account missing data, although we attempted an available case extraction from material on FDA website yielding incomplete data collection'. (Table 1 presents the sample analyzed and the missing outcome data per arm). |
| 21177401 | 2011 | '[...] the main analysis was conducted on an intention-to-treat basis in seven cases (with five of these reporting the use of a modified intention-to-treat population, which included only patients that received at least one dose of the study drug). The population included in the analysis was unclear in three cases'. |
| 21398351 | 2011 | 'Analyses were performed on data that were explicitly reported in the individual papers, with no imputations for data that were not reported. When available, we analysed the intention to treat population; when this was not possible, we used data from the last observation carried forward'. |
| 22036276 | 2011 | 'The loss to follow-up varied from 7% to 26%, and the presented analyses do not take account of how the censored datasets may have skewed the outcomes'. |
| 22582102 | 2012 | Information on randomised is provided but not on the analysed; the reviewers explicitly mention that they analyzed the last available measurement (‘[…] reported at the last available follow-up measurement of the double-blind randomized period of the RCTs’). |
| 23718547 | 2013 | 'All analyses were based on the intention-to-treat (ITT) data reported in the manuscripts published for the included RCTs'. By comparing Appendix B (it gives information on randomized and completers sample) with Table 1, totals in latter are smaller in all studies. |
| 25006351 | 2014 | 'All meta-analyses were conducted on a modified intent-to-treat population, which is defined as the set of patients who were randomised and received at least 1 dose of study medication'. Table 2 provides information on the total randomized sample per trials, whereas Figure 3 provides information on the analyzed sample. It is obvious that none of the trials has been analyzed as intention-to-treat but either as modified intention-to-treat or available case analysis, instead. |
| 25036226 | 2015 | 'Since all included studies but one (Kakkar et al. 2009) employed an intention-to-treat-based last observation carried forward (LOCF) approach, we did not require sensitivity analysis by exclusion of studies not analyzed on an ITT basis'. However, it is not mentioned how missing outcome data at baseline where handled. |
| ***Imputation with or without LOCF*** | | |
| 19185342 | 2009 | 'Responders to treatment were calculated on an intention-to-treat basis […] imputed outcomes for the missing participants assuming that they did not respond to treatment'. |
| 21097801 | 2011 | Comparing Table 2 with Table w2 (Appendix) the number analyzed are the number randomized. They specify also the percentage completers in each trial which indicates that missing outcome data might have been indeed imputed but without information on LOCF. The reviewers reported that they used intention-to-treat without further clarifications, though. No information on how each trial handled missing outcome data. |
| 21295192 | 2011 | By comparing the totals in the efficacy outcome with those in the dropout outcome, we might infer that intention-to-treat with imputation has been done. The reviewers reported that they planned intention-to-treat analysis. |
| 21851976 | 2011 | Some trials had already done LOCF and the reviewers used intention-to-treat as all missing cases are failures for the completely missing outcome data. |
| 21831256 | 2012 | The reviewers report that they employed intention-to-treat analysis. In addition, it has been explicitly reported that each trial applied intention-to-treat analysis. |
| 22107456 | 2012 | No information available on the analyzed but only on the randomized sample for each trial and no information on how MOD have been handled in each trial. The reviewers, though, reported that they did intention-to-treat analysis with clarifications (all missing cases are failures). |
| 23042705 | 2013 | 'We used the intention-to-treat analysis'. Table S2 includes information on percentage withdrawal and percentage lost to follow-up in addition to number of subjects per arm, whereas data are provided directly as intention-to-treat. Intention-to-treat might be feasible. |
| 23744561 | 2013 | The authors explicitly reported that intention-to-treat analysis was applied and by comparing totals reported in the Table of Characteristics with those in Analysis 1.1, we conclude that intention-to-treat analysis might have been indeed applied. |
| 24284258 | 2013 | 'Data from intention-to-treat (ITT) analyses were extracted. […] Should a trial not report ITT data then missing data were treated as treatment failures to allow the analysis to conform to an ITT analysis'. Totals in Figure 2 (primary analysis) agree with totals on dropout (Figure 6). |
| 24448972 | 2014 | 'For trials including participants with missing data, intention-to-treat (ITT) estimates were used as reported by the trial authors. If ITT estimates were unavailable, participants with missing data were considered nonresponders'. Table e4 (information on number randomized) agrees with Table e2 (efficacy outcome) and Table e4 (acceptability) in terms of total analyzed. |
| 25583895 | 2015 | 'Patients with missing data were considered nonresponders or nonremitters. In cases where responder and remission data were not reported, we imputed it from available score data'. By comparing the totals in the efficacy outcome (Figure S1) with those in the dropout outcome (Figure S2), it seems that intention-to-treat must have been employed. |
| ***Combination of Imputation and Available case analysis*** | | |
| 21678632 | 2011 | Reviewers reported that 'If true intention-to-treat results were not reported, but loss to follow-up was very small, we considered these results to be intention-to treat results'. |
| 21920996 | 2012 | 'Where studies did not report intent to treat, we analyzed outcomes as all-patients randomized'. There is also information on how missing outcome data have been handled in each trial. |
| 23020934 | 2012 | 'The summary statistics on the relevant outcomes were presented according to the number of events reported in the original studies or sub-studies intent-to-treat analysis. Where studies did not report intent-to-treat, we analyzed outcomes as all-patients randomized'. Not at all a genuine intention-to-treat analysis for the latter studies. |
| 22739992 | 2013 | Table 1 refers to 'Outcome analysed (no. of trials/no. of analysed patients)' and the reviewers explicitly mentioned that 'Whenever available we used results from intention-to-treat analyses (only 20% of the included studies)'. |
| 23658937 | 2013 | 'We calculated rates using the number of all randomized patients as the denominator to reflect a true intention-to-treat analysis'. In Table D-1 there is distinction between analyzed and randomized sample for each trial and there is information on how missing outcome data have been handled in each trial (Table E). |
| 23723742 | 2013 | 'Results from intention-to-treat analysis were preferred over results from completer analyses'. According to Table S2, there is a combination of intention-to-treat and available case analysis as reported in the trials. |
| 24671923 | 2014 | 'When possible, we used data from intention-to-treat (ITT) analyses from all randomly assigned participants'. According to the Risk of Bias assessment, it appears that a combination of ITT (not genuine most of the times) and per protocol analysis has been implemented in the selected NMA. |
| 24697518 | 2014 | 'Intention-to-treat analyses were based on the total number of randomly assigned participants, irrespective of how the original study investigators analysed the data, by assuming all drop-outs to be non-responders'. In legend of Table 1, it is explicitly indicated for which trials the randomized sample was not available but the analyzed was used, instead. |
| 25066766 | 2014 | '[…] and results from intention-to-treat (ITT) analyses that included all randomized patients took precedence over results from analyses that excluded patients'. According to Table 1, some trials offered intention-to-treat whereas others either available case analysis or not a clear analysis method. |
| **Implicit judgment of the actual method** | | |
| ***Available case analysis with or without LOCF*** | | |
| 20346263 | 2010 | By comparing number randomized in Table 6 with number analyzed in Appendix 9 the latter seems to be smaller. |
| 20600036 | 2010 | In Table 1 (ALT norm), number randomized is much larger than analyzed sample and hence, available case analysis without information on LOCF might have been employed. |
| 22142554 | 2011 | Studies that employed LOCF have been reported in the quality assessment as having done intention-to-treat (see, Appendix 4 and Appendix 3). This is not a genuine intention-to-treat. |
| 23014668 | 2012 | Even though not explicitly defined, Table 1 (Characteristics of included trials) displays the randomized sample, whereas Table 2 (raw outcome data for all outcomes) the analyzed sample (see, the totals in all outcomes) and the latter is smaller. |
| 23659562 | 2013 | By comparing the totals in the dropout outcome (Figure 2C) with the totals in the primary outcome (Figure 2A), the latter are smaller. |
| 24773456 | 2014 | In Table 1, the number analyzed is much smaller than the number analyzed (explicitly mentioned) in all by one trial. |
| 24965841 | 2014 | Supplementary Table 3 presents information both on number randomized and number analyzed and results are presented as available case analysis (without information on LOCF). In addition, percentage dropout can be estimated for each arm of every trial. |
| 24989022 | 2015 | Table 1 explicitly reports how each trial handled missing outcome data for every outcome. For all-cause mortality, modified intention-to-treat has been used in all trials. |
| 26315653 | 2015 | Explicit information is provided in Table 1: the number of included patients at follow-up is smaller than the number of patients at baseline in all studies. |
| 27323781 | 2016 | Through Table 1, number randomized is larger than the number eligible. |
| ***Intention-to-treat analysis with or without LOCF*** | | |
| 21333232 | 2011 | Number randomized equals number analyzed but there is no reference to LOCF. All included trials applied intention-to-treat analysis. |
| 22304415 | 2012 | Sample in total adverse events equals sample in dropouts per arm (Table 2 using sample in dropouts as proxy of the randomized sample). No information on how missing outcome data have been handled in each trial. |
| 25486075 | 2014 | Number randomized has been also analyzed, by comparing the Table of characteristics and the information on attrition bias with the analyzed outcome. |
| 26062437 | 2015 | It is stated that the number analyzed is the number randomized in all trials. |
| 26384035 | 2015 | By comparing the analyzed totals (Analysis 1, Comparison 1) with the randomized total in Risk of Bias table it is obvious that they applied genuine intention-to-treat analysis, but without information on the assumption about missing cases. |
| ***Combination of Imputation and Available case analysis*** | | |
| 21370258 | 2011 | By comparing Table 1 (number randomized) with Figure 1 (number analyzed), some trials might have applied intention-to-treat analysis (without information on the imputation) and others available case analysis. No information on how trials or the reviewers handled missing outcome data. |
| 21406529 | 2011 | In sensitivity analysis the reviewers kept only trials with completed outcomes and hence, there is a combination of intention-to-treat and available case analysis as reported in the trials. No information available on how missing outcome data have been handled in each trial. |
| 21729036 | 2011 | By comparing Figure 1 with Figure 2 and account for the information on intention-to-treat or per protocol analysis in Table S1, some trials did intention-to-treat (no further information) and other analyzed as available case analysis. |
| 21992870 | 2011 | It appears that the authors analyzed the data as reported by the trials and some trials may have done intention-to-treat, whereas others clearly did available case analysis (compare totals in Appendix with Figure 2). |
| 22044854 | 2011 | The reviewers analyzed the data as reported 'All studies considered non-completion as failure and reported data for the randomized patients who received at least 1 dose (ie, intent-to-treat exposed population), except for the study by Riddler et al, which ignored missing data'. Combination of (modified) intention-to-treat analysis and available case analysis. |
| 21910698 | 2012 | The reviewers did not mention how each trial handled missing outcome data or how they handled missing outcome data but by comparing Table 1 (characteristics of trials) with Table 2 (analyzed data) the sample size is equal or smaller in the latter for some trials. |
| 22284386 | 2012 | There is information on analyzed and randomized sample for each trial. There is also information on how MOD have been handled in each trial ('the majority […] analyzed data on an intention-to-treat basis (13/16)' and Table 1 and Table 2). |
| 22700784 | 2012 | 'We carried out […] on an intention to treat basis, according to PRISMA […]'. There is no explicit information on the analyzed and randomized sample for each trial but some trials that investigated rivaroxaban appear to have smaller analyzed sample (Figure 2) than that reported in the Table of characteristics (Table 1). |
| 22717535 | 2012 | Table 3 explicitly mentions how each trial handled missing outcome data. Reviewers might have analyzed the outcomes as reported in the trials. |
| 23150473 | 2012 | Table 1 reports which trials employed intention-to-treat or available case analysis but reviewers don’t report how they planned to handle missing outcome data. |
| 22703832 | 2013 | There is no distinction between analyzed and randomized sample for each trial but there is information on how each trial addressed missing outcome data. In addition, reviewers don’t report how they planned to handle missing outcome data. |
| 22776828 | 2013 | Table 1 states intention-to-treat analysis, but total samples in Figure 2 (primary outcome) are smaller than those in Figure 3 (dropout outcome) for some trials. |
| 25263803 | 2015 | 'The denominator used in all trials was based on a modified intention-to-treat (ITT) analysis […]'. In Table 1 many studies provide data both as intention-to-treat and as modified intention-to-treat and others as intention-to-treat only. |
| **Unclear judgment of the actual method** | | |
| 19144173 | 2009 | There is no information available on the analyzed and randomized sample for each trial as well as no information on how missing outcome data have been handled in each trial. The reviewers don’t report how they planned to handle missing outcome data. |
| 19173737 | 2009 | There is no information available on the analyzed and randomized sample for each trial in order to make sure that are included trials truly did an intention-to-treat analysis or a combination of intention-to-treat and available case analysis. |
| 19558609 | 2009 | It is not mentioned whether the analyzed sample is actually the randomized sample in order to judge whether a genuine intention-to-treat analysis has been applied. |
| 19954682 | 2009 | 'Analyses were carried out for the intention to treat (ITT)'. Results are provided narratively without tabulation of the study results in order to understand how many patients were analyzed out of the total randomized. |
| 20418173 | 2010 | Table 1 reports the number of completers out of those randomized and no information is provided on how missing outcome data were addressed on the primary outcome (i.e. as intention-to-treat or available case analysis). |
| 20934984 | 2011 | There is no information available on the analyzed sample but information on the randomized sample for each trial only. There is no explicit information on which trials used available case or intention-to-treat analysis. |
| 21224324 | 2011 | The reviewers report that they employed intention-to-treat analysis. Information on randomized sample is provided but there is no information available on analyzed sample to judge whether intention-to-treat analysis is genuine. |
| 21227948 | 2011 | They provide the number randomized and the number dropped (Table A1) but there is no information on the analyzed data. Judgements for a genuine claimed intention-to-treat analysis cannot be made. |
| 21868065 | 2011 | There is information on completers but nowhere is stated the analyzed sample as there is no raw data. The reviewers provide directly the NMA results only. |
| 22292469 | 2012 | Reviewers reported that 62% of the trials applied intention-to-treat analysis but it is not specified in the Table of characteristics which ones exactly. There is no information available on randomized and analyzed sample and reviewers don't report how they handled missing outcome data. |
