# Supplementary material for: A systematic survey shows that reporting and handling of missing outcome data in networks of interventions is poor
Source: BMC Med Res Methodol. 2018 Oct 24;18:115. doi: 10.1186/s12874-018-0576-9 (PMC6201503; doi:10.1186/s12874-018-0576-9)
Supplement: Supplementary file 7 — Appendix G. Explanations to support the strategy applied in primary and sensitivity analysis. (DOCX 22 kb) [file 12874_2018_576_MOESM7_ESM.docx]

**Appendix G. Explanations to support the strategy applied in primary and sensitivity analysis**

| **PMID** | **Year** | **Quotations** |
| --- | --- | --- |
| 19185342 | 2009 | "To carry out a clinically sound analysis, we used a conservative approach and imputed outcomes for the missing participants assuming that they did not respond to treatment". |
| 19954682 | 2009 | "Analyses were carried out for both the ITT (intention to treat; representative of the entire population recruited in the trials) and the ITTI (intention to treat, confirmed, influenza positive) populations whenever possible". |
| 21040531 | 2010 | "However, concerns exist regarding whether it is appropriate to use LOCF in analyses involving progressive conditions or in situations where it may not be possible to determine whether missing data are non-random. Specifically for epilepsy, LOCF analysis yields seizure-free rates that are higher than the true clinical situation. Therefore, we conducted two sensitivity analyses [...]". |
| 22516128 | 2012 | "To get an impression of the effect of withdrawals, we performed two types of (sensitivity) analysis: one in which patients lost to follow-up were considered cures and one in which they were considered failures". |
| 23102749 | 2012 | "To adhere to review guidelines and clinical opinion […]". |
| 23810019 | 2013 | "Dichotomous outcomes will be analysed on an intention-to-treat (ITT) basis: drop-outs will always be included in this analysis. The rule is important for the outcome use of antiparkinson medication at least once. When data on drop-outs are carried forward and included in the evaluation (Last Observation Carried Forward, LOCF), they will be analysed according to the primary studies; when dropouts are excluded from any assessment in the primary studies, we will assume that the dropouts did not receive antiparkinson medication, because it is likely that another assumption (all dropouts have received antiparkinson medication) would overestimate the percentage of people with movement disorders which is relatively low under treatment with the newer antipsychotic drugs". (Protocol in Supplementary Material) |
| 24736585 | 2014 | "We used a conservative approach [alike Cipriani et al on antidepressants] and imputed outcomes for the missing participants, assuming that they did not respond to treatment". |
| 25066766 | 2014 | "[…] ITT analyses have been shown to results in conservative effect estimates […]". |
| 25249164 | 2014 | "To accurately compare all treatments, it was considered that working on OC (observe cases) data was more adequate in the context of active-to-active indirect comparisons in MDD. Nevertheless, in the absence of OC summary statistics, LOCF summary statistics were considered". |
| 25435923 | 2014 | "The information about the endpoint achievement was aimed to reflect the intention-to-treat population. However, there were some occasional post-randomization exclusions in some trials, and so the clinical material actually adopted the so-called modified intention-to-treat population". |
| 25263803 | 2015 | "(Modified ITT) was preferred over true ITT analysis (wherein all dropouts are assumed to be treatment failures) due to high loss to follow-up in some of the trials". |
| 27876667 | 2017 | "To perform a clinically sound analysis, […]". |
